# Postural adjustments and perceptual responses of Nordic running: concurrent effects of poles and irregular terrain

**DOI:** 10.1007/s00421-023-05397-9

**Published:** 2024-01-17

**Authors:** André Ivaniski-Mello, Diana Maria Cubillos-Arcila, Stefano Dell’Anna, Lucas de Liz Alves, Flávia Gomes Martinez, Cosme Franklim Buzzachera, Jonas Alex Morales Saute, Leonardo Alexandre Peyré-Tartaruga

**Affiliations:** 1 LaBiodin Biodynamics Laboratory, School of Physical Education, Physiotherapy and Dance, Universidade Federal do Rio Grande do Sul, Felizardo Street, 750, Porto Alegre, 90690-200 Brazil; 2 Graduate Program in Medicine, Medical Sciences, Universidade Federal do Rio Grande do Sul, Porto Alegre, Brazil; 3 Department of Public Health, Experimental Medicine and Forensic Sciences, University of Pavia, Pavia, Italy

**Keywords:** Locomotion, Poles, Bilateral coordination, Spatiotemporal, Motor control, Pleasure

## Abstract

**Purpose:**

In the natural environment, humans must continuously negotiate irregular and unpredictable terrain. Recently, the poles have been extensively used during trial running events. However, we know little about how humans adjust posture and bilateral coordination to use poles in irregular terrain. Here, we compared kinematics, bilateral coordination and perceptual responses between regular (compact dust) and irregular terrain (medium-length grass) during running at preferred speed with and without poles.

**Methods:**

In this transversal observational study, thirteen young healthy adults (8 men; mean ± SD; age 29.1 ± 8.0 years, body mass 76.8 ± 11.4 kg; height 1.75 ± 0.08 m) were evaluated during running at a self-selected comfortable speed with and without poles on regular and irregular terrains.

**Results:**

Our results show that, despite more flexed pattern on lower-limb joints at irregular terrain, the usage of poles was not enough to re-stabilize the bilateral coordination. Also, the perceived exertion was impaired adding poles to running, probably due to more complex movement pattern using poles in comparison to free running, and the invariance in the bilateral coordination.

**Conclusion:**

Besides the invariability of usage poles on bilateral coordination and lower-limb kinematics, the runners seem to prioritize postural stability over lower limb stiffness when running in medium-length grass given the larger range of ankle and knee motion observed in irregular terrain. Further investigations at rougher/hilly terrains will likely provide additional insights into the neuromotor control strategies used to maintain the stability and on perceptual responses using poles during running.

**Supplementary Information:**

The online version contains supplementary material available at 10.1007/s00421-023-05397-9.

## Introduction

Humans are routinely exposed to uneven terrain, where unexpected, variably compliant surfaces compromise their ability to locomote efficiently, with negative consequences on their mobility and daily life activities (Kent et al. 2019). Walking and running over irregular terrain, for example, presents considerable control challenges compared to flat, smooth terrain and limits anticipation of task demands. Challenging, irregular terrain can also disturb balance during locomotion and increase the likelihood of falls (Blair et al. 2018). Whether walking or running over irregular terrain might also increase the risk for non-fall-related injuries is still uncertain and remains to be elucidated. It is well established, however, that training on such challenging surfaces is particularly effective since it stimulates neuromuscular adjustments to counteract the unanticipated perturbations induced continually (Blair et al. 2018).

In recent years, there has been considerable interest in the utility of running on an unpredictable, irregular surface as a strategy for improving the individual ability to locomote efficiently. The stride cycle of running is divided into contact and aerial phases, and one of the main differences between walking and running is the absence of double support in the latter. During the contact phase of running, the lower limb applies force on the ground, accelerating the body. The aerial phase initiates with the take-off of the foot, and then the body has no more contact with the ground and is subject only to the effects of gravitational and air friction forces (Cavagna et al. 2008). Running is influenced by the interaction of mechanical and physiological components (di Prampero 2003; Joyner and Coyle 2008; Tartaruga et al. 2012), such as terrain characteristics. Prior reports have confirmed that surface regularity can affect running biomechanics (Müller et al. 2010; Müller and Blickhan 2010; Hébert-Losier et al. 2015), where a locomotor pattern characterized by a more flexed lower limb stance and a shorter stride length emerge (Groucho running) (McMahon et al. 1987). A more flexed lower limb reduces vertical body sway, leading to a more stable movement (Peyré-Tartaruga et al. 2021). In fact, Hébert-Losier et al. (Hébert-Losier et al. 2015) reported increased hip and knee flexion during amateur running at 3.8 m/s on irregular surfaces (compact dust on cement vs. grass), which could be a compensatory strategy to improve postural stability. Running in a softer terrain, otherwise, can cause an increase in stiffness, resulting in a more extended position of the lower limb joints (Ferris et al. 1998). Terrain characteristics, therefore, will lead to a more-flexed or -extended pattern from lower limb joints during running on an irregular surface, depending on whether the motor control adjustment favors postural stability or lower limb stiffness, respectively.

Running on an irregular terrain not only implies greater challenges for movement control but also for visual and somatosensory activations. Hence, running with poles has recently been introduced in trail running and mountain (ultra)marathon events to minimize such disturbances on motor, visual, and somatosensory interactions caused by an unpredictable, irregular surface. The use of poles during walking elicits pronounced effects on dynamic stability, particularly mediolateral stability, and trunk coordination (Peyré-Tartaruga et al. 2022), and promotes long-term benefits in healthy adults and diseased patients, such as those with Parkinson's disease (Monteiro et al. 2017). The benefits of using poles during running, otherwise, are still poorly understood and largely understudied in the field of biomechanics. For example, there are currently no published reports on the effects of running with poles on spatiotemporal, lower limb joint kinematics, and coordination responses. It is well known, however, that locomotion with poles is a motor dual-task condition (Yogev-Seligmann et al. 2008) where the movement is coordinated with the control of the poles' position in space. It might indicate that running with poles decreases bilateral coordination stability, as the runners should synchronize the lower limbs' movement with the correct positioning of the poles on the floor. The use of poles, however, might contribute to the stabilization during running on hilly and irregular terrains.

The purpose of this study was to investigate the effects of running with poles in both regular (compact dust) and irregular (medium-length grass) surfaces on self-selected comfortable speed, stride length and time, angular parameters of the lower limb joints, bilateral coordination, and perceptual responses in healthy adults. Our first hypothesis was that running in irregular terrain compared to regular terrain would have a more flexed pattern in the lower limb joints, with a reduced speed and diminished stride length and time. We also expected that the use of poles in irregular terrain would attenuate the probable impairments in the bilateral coordination, particularly in the irregular terrain.

## Methods

### Design and participants

This is a transversal observational study reported accordingly to STROBE checklist (von Elm et al. 2008) (Supplementary Material 1) and it was approved by the by the Ethics Committee of Universidade Federal do Rio Grande do Sul (No. 1.894.356) and conducted in accordance with the Declaration of Helsinki.

Thirteen young, apparently healthy adults (8 men; mean ± SD; age 29.1 ± 8.0 years, body mass 76.8 ± 11.4 kg; height 1.75 ± 0.08 m) were evaluated. The inclusion criteria were: no musculoskeletal problems (muscle, tendon, joint or bone pain or injury); no neurological or cardiopulmonary disorders; physically active; and fully familiar with using poles during running (minimum of 6 months of experience in running with poles) and the testing procedures. The exclusion criterion was the inability of performing the running tests. The sample size was calculated (G*Power, v.3.1.9.7, University of Kiel, Germany) using the effect size of the running speed on regular and irregular terrain from Hébert-Losier et al. (Hébert-Losier et al. 2015), and we found a sample size needed of 13 participants (*α* = 0.05, *β* = 0.95). Before beginning the tests, all participants read and signed a free informed consent form.

### Data collection and analysis

All participants attended one preliminary session and one experimental session with repeated measures involving running on two surfaces. During the preliminary session, they underwent screening, anthropometric measurements, and familiarization with the testing procedures. During the experimental session, otherwise, all participants were randomly assigned, in a counterbalanced fashion, to run at a self-selected, comfortable speed with and without poles on regular and irregular terrains. Hence, each participant ran under four experimental conditions: free running on regular (FR) and irregular terrain (FI), and Nordic running with poles on regular (NR) and irregular terrain (NI). The regular terrain comprised a road surface, which was firm, deprived of obstacles, and made of compact dirt resting over cement. The irregular terrain, in turn, included a softer path mainly constituted of medium-length grass with a couple of tree branches and logs. Both surfaces were flat, dry, and delimited throughout the study.

All participants were instructed to arrive rested and fully hydrated and refrain from alcohol and caffeine consumption for at least 24-h prior to each experimental visit. They were also asked to abstain from strenuous exercise for at least 48-h before each visit to the laboratory. Preliminary and experimental sessions were performed at the same time of the day to avoid circadian variance and under similar weather conditions with no precipitation.

During the experimental session, each participant ran at a comfortable self-selected speed in a 4 × 30 m straight path on each surface condition. The intermediary 20 m was considered for data analysis to discard both the acceleration and deceleration phases. Each running trial was repeated after 2-min of passive recovery. In both Nordic running conditions (NR and NI), all participants were instructed to adopt the diagonal technique recommended by the International Nordic Walking Federation (Peyré-Tartaruga et al. 2022). The Nordic poles' (mass 155 g each, XTR, Gabel srl., Rosà, Italy) length was defined by multiplying the participants' height by 0.68 with a tolerance of 0.025 m (INWA 2015; Peyré-Tartaruga et al. 2022).

Whole body motion was monitored during running using seven inertial measurement units (IMU) (Ultium Motion, Noraxon Inc., Scottsdale, USA) with a sampling frequency of 200 Hz. The IMU sensors were attached with elastic straps on the right and left lower limbs (foot, shank, and thigh) and on the sacrum. The sensor locations followed the instructions of the MR3 software (Noraxon Inc., Scottsdale, USA) and were calibrated in a standing position before the dynamic data collection. The IMU raw data was processed with a bult-in function in MR3 software, exporting the spatiotemporal and angular data. For more details on IMU Ultium Motion system data collection and processing, please refer to (Bartoszek et al. 2022). Although the study of joint angles with the IMU system is not interchangeable with the gold standard optoelectronic measurement system, the use of IMU still can provide important information about intra-individual changes of joint angles (Bartoszek et al. 2022). In addition, we decided to evaluate the lower limb joints angles only in the sagittal plane (flexion–extension), where a strong correlation value (*r*) between IMU and the optoelectronic system was found (hip: 1.0; knee: 0.99; ankle: 0.98) (Bartoszek et al. 2022).

The right lower limb was considered to analyze spatiotemporal (stride length, stride time, percentage of contact and aerial phases) and angular (hip, knee, and ankle flexion–extension angles) parameters. The range of joint motion (ROM) during the full stride cycle and during the contact and aerial phases were also analyzed.

The bilateral coordination was determined by the phase coordination index (PCI) (Plotnik et al. 2007; Correale et al. 2022) using both step time and stride time. Bilateral accuracy is the left–right stepping time difference, while bilateral variability is the variation of the accuracy from many sequential strides. First, the Phi value (in degrees) was calculated (Eq. 1) for each gait cycle *i*

1 $$Phi(i)=\frac{\text{Step time}(L{L}_{short-swing})}{\text{Stride time}(L{L}_{long-swing})}*360^\circ$$

Then, the accuracy (in %) was calculated (Eq. 2)

2 $$Accuracy=\frac{\left|Phi-180\right|}{180}*100^\circ$$

Variability (in %) with Eq. 3

3 $$Variability=\frac{Ph{i}_{SD}}{Ph{i}_{Mean}}*100$$

Finally, PCI (in %) was calculated by Eq. 4

4 $$PCI=Accuracy+Variability$$

Exertional and affective responses were assessed at the end of each running trial. All participants rated “how” and “what” they felt at these moments (da Silva et al. 2011). Ratings of perceived exertion (RPE) for the overall body were determined by the 0–10 Borg RPE scale (Borg 1990). All participants were previously anchored to the scale using memory-anchoring procedures (da Silva et al. 2011). Affective valence, otherwise, was measured using the Feeling Scale (Hardy and Rejeski 1989), an 11-point single-item measure ranging from “very bad” (− 5) to “very good” (+ 5). Standard definitions of perceived exertion and affective valence and separate instructional sets for both scales were read to the participants before the tests (Vandoni et al. 2016).

### Statistical analysis

Normality of distribution for all datasets was assessed with the Shapiro–Wilk test. Unless otherwise informed, data are shown as means and standard deviation (SD). Generalized estimating equations (GEE) were used to examine the effects of running mode (free and Nordic) and surface (regular vs. irregular), and their interaction, on the selected dependent variables. As the GEE method can model response variable from any exponential family distribution, the normality of the data was not tested (Nikita 2014). However, the linear and gamma distributions were tested for each dependent variable, and the distribution with best-fit was chosen for further analysis. Missing data cases were excluded from analysis. These statistical analyses were performed in SPSS software (v. 22, Statistical Package for Social Sciences, IBM, USA). The smallest worthwhile change (Hopkins 2004) was calculated as 20% of the SD from the free running on regular terrain considered as the baseline condition.

In addition to the traditional scalar analysis of the angular responses by ROM, a statistical parametric mapping (SPM) was used to analyze the continuous topological response of the lower limb joints during the entire running stride cycle (Pataky 2010). The SPM was conducted with a two-way repeated measures analysis of variance (ANOVA) and post hoc with Bonferroni correction (SMP1d v. 0.4, https://www.spm1d.org) (Pataky 2012) was employed to compare the continuous curves of joint's angular positions along the stride cycle among the experimental trials. The significance level adopted was *α* = 0.05.

## Results

A total of 3052 strides (1526 from each foot) were analyzed. The dataset with the individual data is disponible in Figshare (DOI: 10.6084/m9.figshare.22122782). The complete statistical analyses results are disponible in Supplementary Material 2.

### Spatiotemporal and coordination

**Fig. 1:**
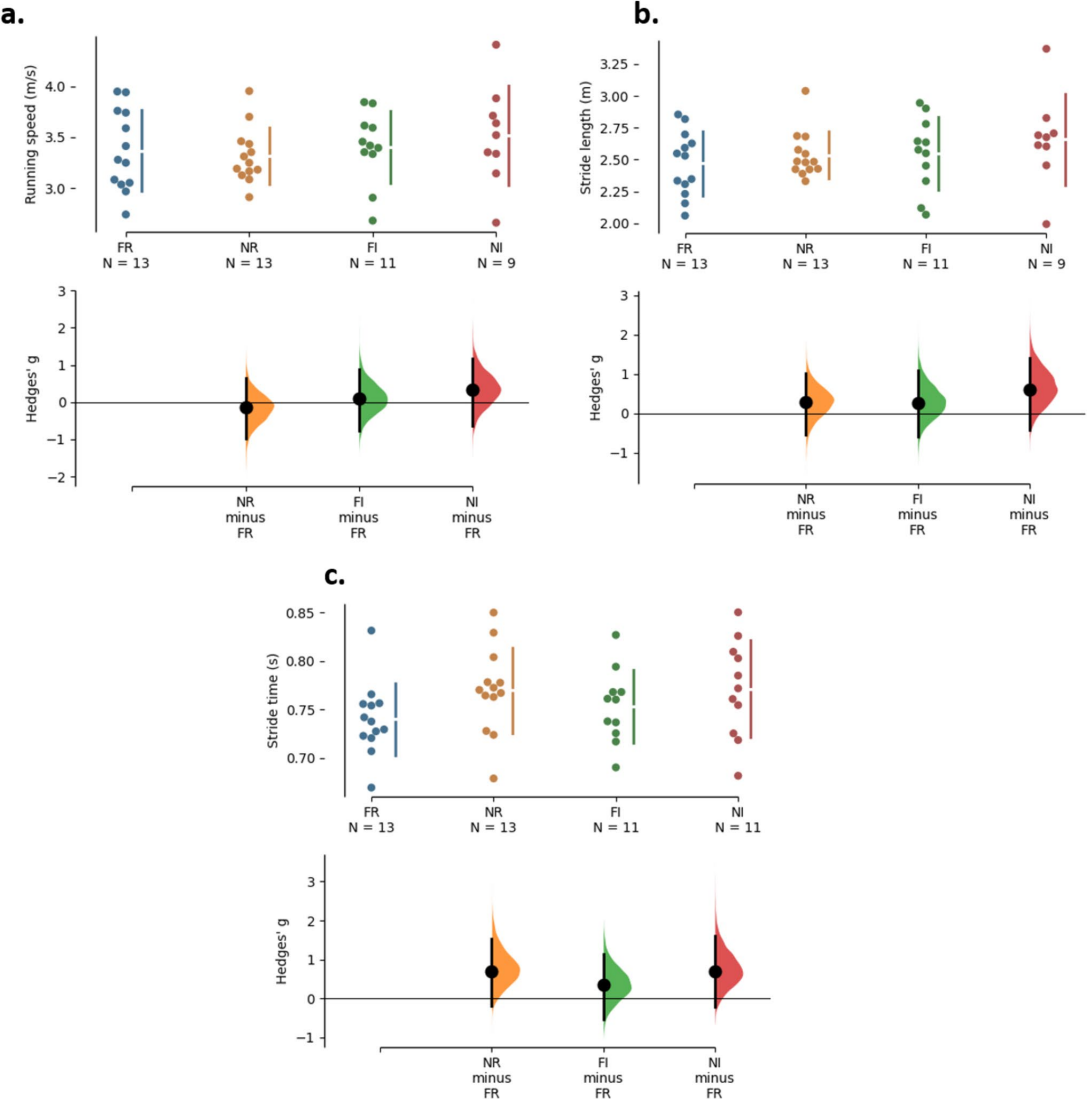
Individual response of running speed (**a**), stride length (**b)**, and stride time in (**c**) of each participant during running at four conditions: free and Nordic running in regular terrain (FR and NR), and free and Nordic running in irregular terrain (FI and NI). The comparison of the response of each participant is given between free vs. Nordic running in the superior panel, and between regular vs. irregular terrain in the lower panel. Below each plot there are the respective graphical representation of the paired Hedges’ *g* effect size (mean and 95% confidence interval). The effect size was calculated in relation to the FR condition

Figure 1 illustrates the individual response of the spatiotemporal parameters of the studied population in each experimental condition. The mean and SD values of these parameters are shown in Table 1. Running speed, stride length, and relative time of contact and aerial phases were affected neither by the running mode (*p* = 0.11 to 0.69) nor by terrain (*p* = 0.14 to 0.33) and their interaction (*p* = 0.22 to 0.60). In contrast, stride time was longer in Nordic running compared to free running (*p* < 0.001), but similar on both surfaces (*p* = 0.45), and without significant interaction between factors (*p* = 0.09).

**Table 1:** Mean and standard deviation of the spatiotemporal variables and scales during running in four conditions: free and Nordic running in regular terrain (FR and NR), and free and Nordic running in irregular terrain (FI and NI)

Variable	FR	NR	FI	NI	Mode	Terrain	Mode*Terrain
Speed (m/s)	3.37 (0.40)	3.32 (0.27)	3.40 (0.35)	3.51 (0.49)	0.66	0.33	0.22
Stride length (m)	2.47 (0.25)	2.54 (0.18)	2.54 (0.29)	2.66 (0.36)	0.11	0.24	0.60
Stride time (s)	0.74 (0.04)	0.77 (0.04)	0.75 (0.04)	0.77 (0.05)	** < 0.001**	0.45	0.09
Contact phase (%)	32.4 (2.4)	32.4 (3.2)	32.0 (3.1)	31.6 (2.9)	0.68	0.14	0.52
Aerial phase (%)	67.6 (2.4)	67.6 (3.2)	68.0 (3.1)	68.4 (2.9)	0.69	0.14	0.54
RPE (0–10)	1.4 (0.1)	1.7 (0.9)	1.8 (0.8)	2.0 (0.8)	**0.02**	0.07	0.28
Feeling (− 5 to + 5)	+ 4.0 (1.3)	+ 3.8 (1.3)	+ 3.1 (0.4)	+ 3.0 (0.6)	0.34	**0.01**	0.90

The p values are presented for the comparisons between conditions for the factor mode of running (free and Nordic running), terrain (regular and irregular), and their interaction (mode*terrain). The statistically significant values of *p* are bolded. The significance level used was *α* = 0.05. *RPE* rating of perceived exertion

The mean and SD values of the bilateral coordination are shown in Table 2. No significant effects of running mode (*p* = 0.10 to 0.30), terrain (*p* = 0.07 to 0.34), and their interaction (*p* = 0.173 to 0.39), were found.

**Table 2:** Mean and standard deviation of the bilateral coordination measured by phase coordinative index (PCI), accuracy and variability during running in four conditions: free and Nordic running in regular terrain (FR and NR), and free and Nordic running in irregular terrain (FI and NI)

Variable (%)	FR	NR	FI	NI	Mode	Terrain	Mode*Terrain
PCI	4.42 (0.37)	4.77 (0.37)	3.62 (0.40)	4.44 (0.40)	0.10	0.07	0.18
Accuracy	1.35 (0.21)	1.46 (0.21)	0.88 (0.23)	1.17 (0.23)	0.30	0.09	0.39
Variability	3.07 (0.23)	3.31 (0.23)	2.74 (0.25)	3.27 (0.25)	0.11	0.34	0.17

The *p* values are presented for the comparisons between conditions for the factor mode of running (free and Nordic running), terrain (regular and irregular), and their interaction (mode*terrain). The significance level used was *α* = 0.05

### Angular

**Fig. 2:**
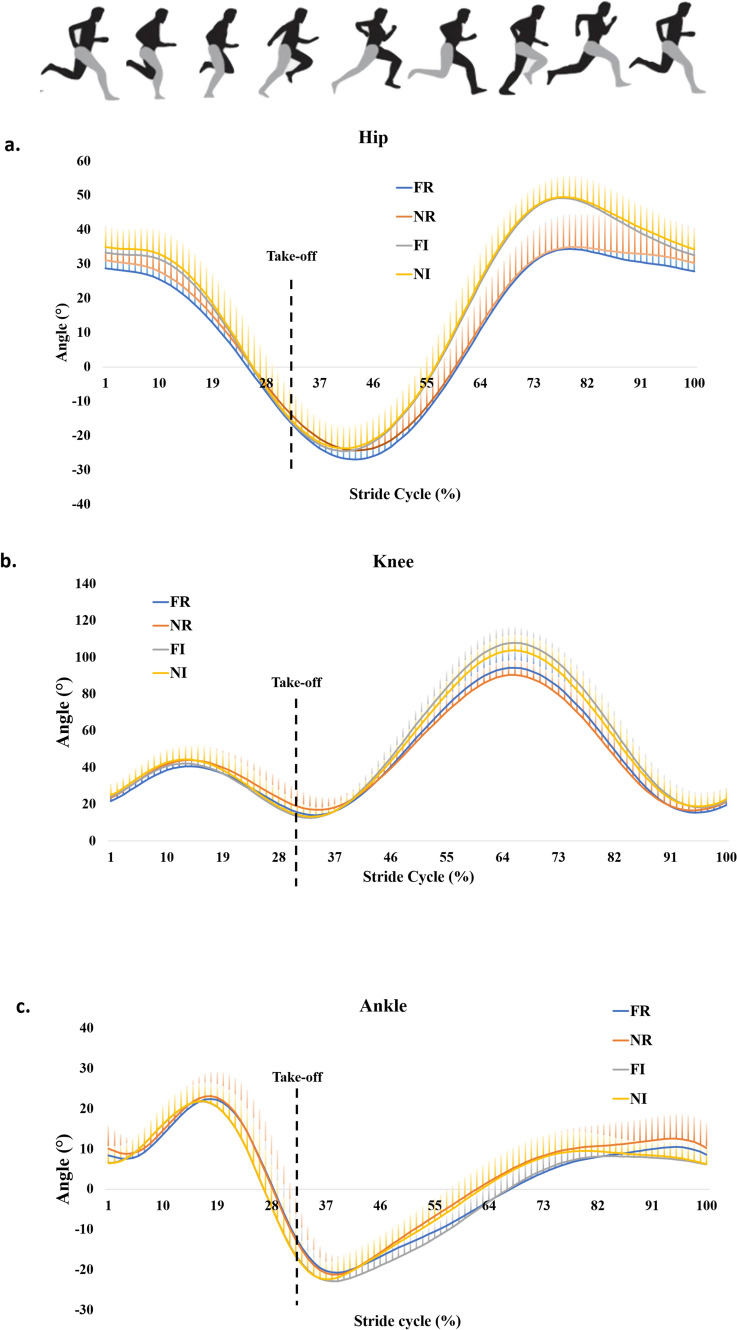
Hip (**a**), knee (**b**) and ankle (**c**) joints’ angle (mean and SD) in the sagittal plane during stride cycle of running at four conditions: free and Nordic running in regular terrain (FR and NR), and free and Nordic running in irregular terrain (FI and NI). The horizontal axis is normalized by the stride time (0–100%). The take-off event is indicated by the vertical dashed line

**Fig. 3:**
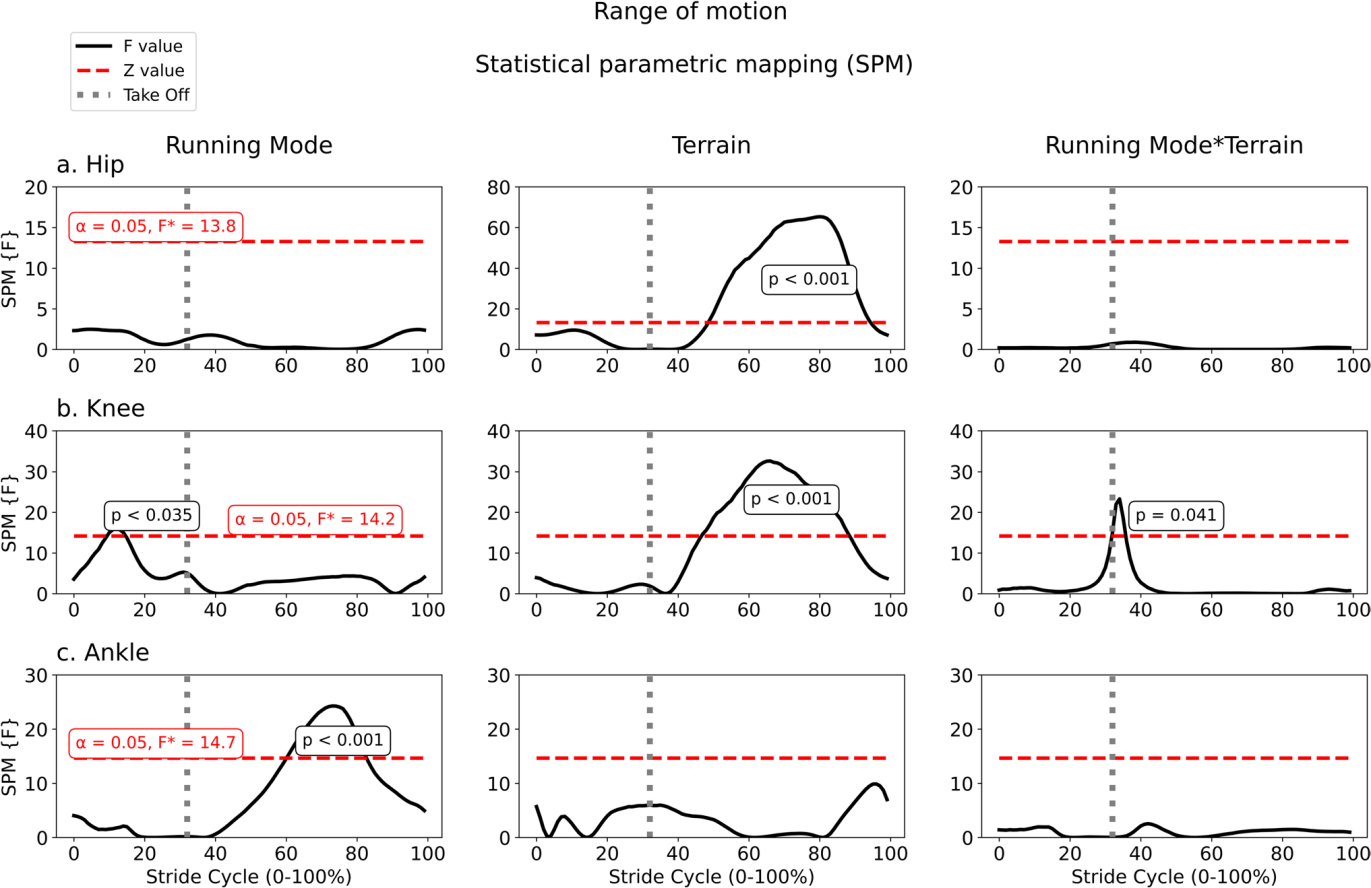
Statistical parametric mapping (SPM) using two-way ANOVA with repeated measures to compare the angular position of hip (**a**), knee (**b**), and ankle (**c**) flexion–extension during running at four conditions: free and Nordic running in regular terrain, and free and Nordic running in irregular terrain. Each row is a joint and each column is a factor. The take-off event is indicated by the vertical dashed line. The critical *F* value and threshold alfa for each analysis are written in red, and they are equal factor within joint. The *p*-values from the suprathreshold cluster periods of each curve are also indicated

Figure 2 depicts the angular position curves in the sagittal plane of each joint along the stride cycle for each running condition, and Fig. 3 contains the SPM results for the main effects of running mode, terrain and their interaction on the angular position each joint along the stride cycle. The running mode affected the knee (contact phase) and ankle (aerial phase), and the knee and ankle were more flexed position during Nordic running. While for the terrain factor, the hip and knee were in a more flexed angles during the aerial phase. Therefore, the running mode seems to affect more distal joints of the lower limb, while the terrain appears to influence more proximal joints.

**Fig. 4:**
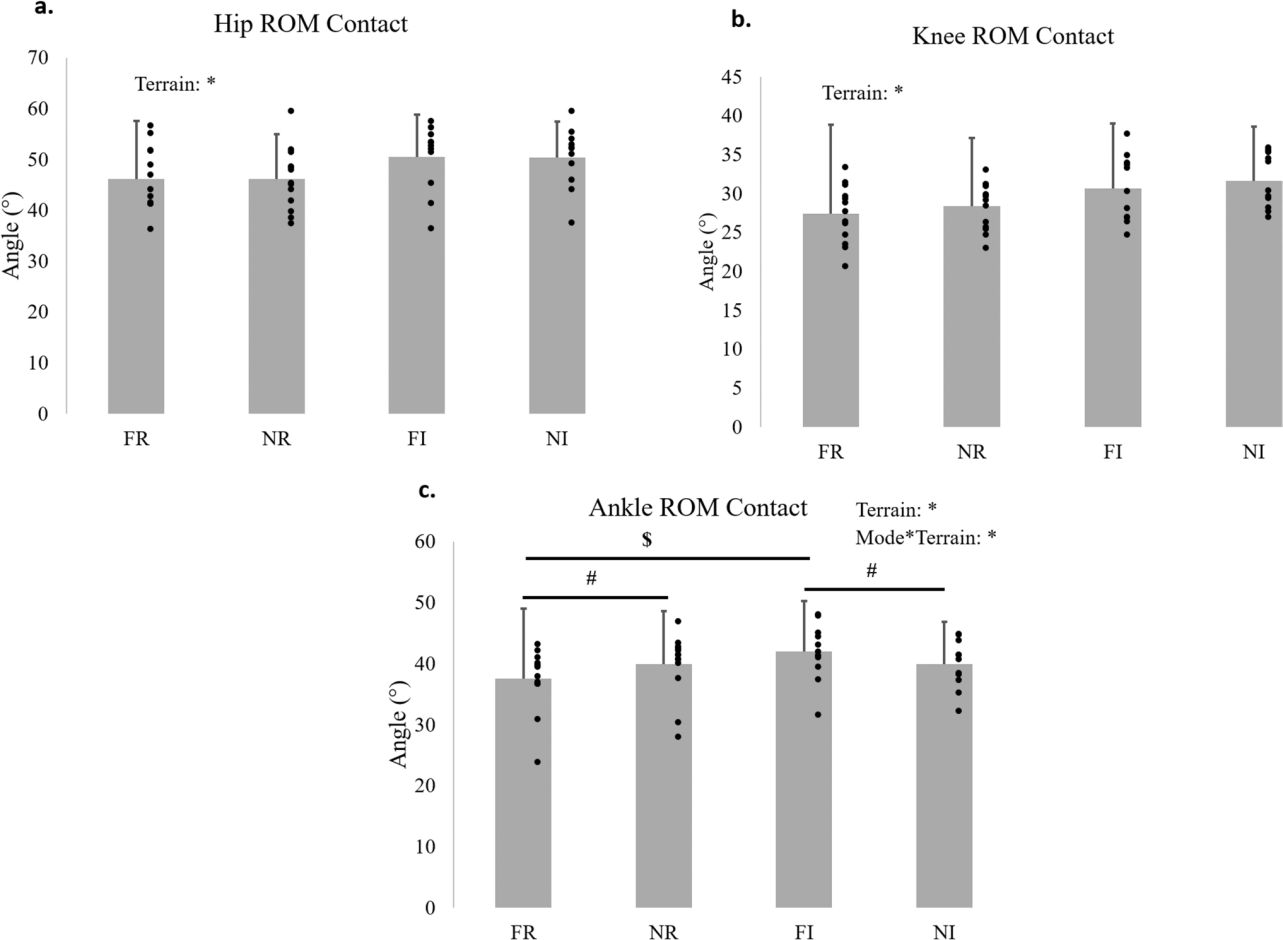
Total range of motion (ROM) in degrees (°) of hip (**a**), knee (**b**), and ankle (**c**) joints during contact phase of running at four conditions: free and Nordic running in regular terrain (FR and NR), and free and Nordic running in irregular terrain (FI and NI). The bars represent the mean and standard deviation values. The dots are the individual values of each subject. The statistically significant effects for terrain and the interaction of mode of running*terrain are indicated by asterisk. If the interaction was significant: the ^#^ symbol represents difference between mode of running for the same terrain, and the ^$^ symbol represents difference between terrain for the same mode of running

**Fig. 5:**
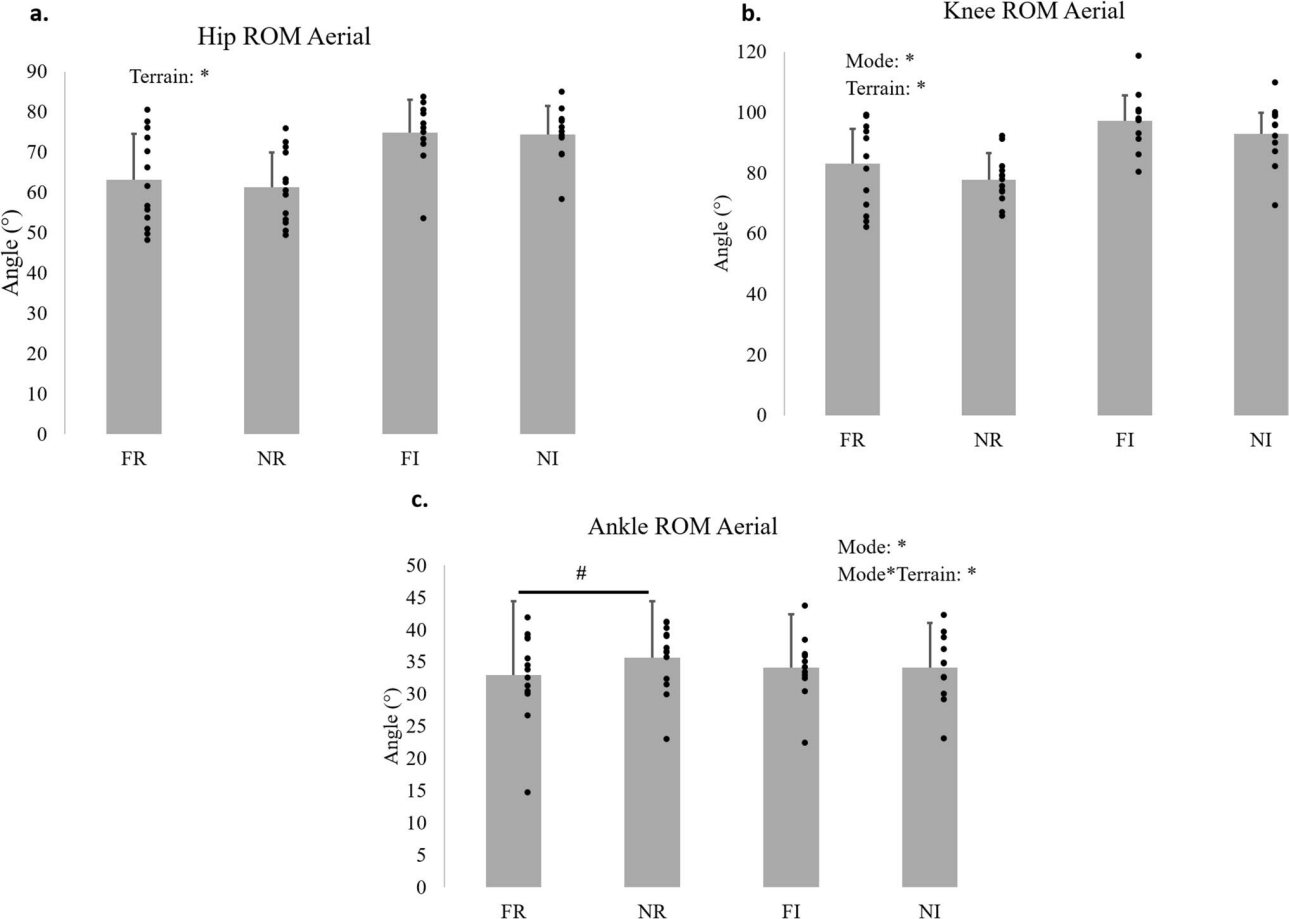
Total range of motion (ROM) in degrees (°) of hip (**a**), knee (**b**), and ankle (**c**) joints during aerial phase of running at four conditions: free and Nordic running in regular terrain (FR and NR), and free and Nordic running in irregular terrain (FI and NI). The bars represent the mean and standard deviation values. The dots are the individual values of each subject. The statistically significant effects for mode of running, terrain and the interaction of mode of running*terrain are indicated by asterisk. If the interaction was significant: the ^#^ symbol represents difference between mode of running for the same terrain, and the ^$^ symbol represents difference between terrain for the same mode of running

Figures 4 and 5 illustrates the angular position curves in the sagittal plane of each joint along the stride cycle of the studied population in each experimental condition. Table 3 shows for each joint the mean and SD values of the ROM in each phase of the stride cycle and the results of the statistical comparisons between mode of running, terrain and their interaction.

**Table 3:** Mean and standard deviation of the total range of motion (in degrees) in sagittal plane from hip, knee, and ankle joints during running in four conditions: free and Nordic running in regular terrain (FR and NR), and free and Nordic running in irregular terrain (FI and NI)

Gait phase	Joint	FR	NR	FI	NI	Mode	Terrain	Mode*Terrain
Contact	Hip	46.2 (6.2)	46.2 (6.1)	50.5 (6.6)	50.4 (6.0)	0.99	**0.001**	0.96
	Knee	27.4 (3.7)	28.4 (3.0)	30.6 (4.3)	31.6 (3.5)	**0.03**	** < 0.001**	0.89
	Ankle	37.6 (5.2)^#^, ^$^	39.9 (5.2)^#^	42.0 (4.7)^#^, ^$^	39.9 (4.0)^#^	0.81	**0.02**	** < 0.001**
Aerial	Hip	63.2 (11.5)	61.3 (9.0)	74.8 (8.3)	74.4 (7.0)	0.46	** < 0.001**	0.44
	Knee	83.1 (14.3)	77.8 (7.9)	97.2 (10.1)	92.8 (10.7)	**0.01**	** < 0.001**	0.54
	Ankle	33.0 (7.0)^#^	35.7 (5.2)^#^	34.1 (5.2)	34.1 (5.4)	**0.04**	0.82	**0.03**

The *p* values are presented for the comparisons between conditions for the factor mode of running (free and Nordic running), terrain (regular and irregular), and their interaction (mode*terrain). If the interaction was significant: the ^#^ symbol represents difference between mode of running for the same terrain, and the ^$^ symbol represents difference between terrain for the same mode of running. The statistically significant values of *p* are bolded. The significance level used was *α* = 0.05

Only the ROM of the ankle joint had statistically significant interaction for mode*terrain in the contact (*p* < 0.001) and aerial phases (*p* = 0.03), therefore, the comparisons among the running conditions for the ankle joint will be described separately from the hip and knee. In the contact phase the use of poles increased the ankle ROM on regular terrain (*p* = 0.003), but decreased the ankle ROM on the irregular terrain (*p* = 0.03). And during the contact phase on irregular terrain compared to regular terrain, the ankle ROM increased only in free running (*p* < 0.001), while was similar between terrains for Nordic running (*p* = 1.00). While for the aerial phase, the ankle had greater ROM in Nordic running compared to free only on the regular terrain (*p* < 0.001), and the modes of running were similar on the irregular terrain (*p* = 0.98).

The hip ROM was not affected by running mode in contact (*p* = 0.99) and aerial (*p* = 0.46) phases. While the knee ROM in Nordic running in comparison to free running was higher during contact phase (*p* = 0.03) and lower in aerial phase (*p* = 0.01). And running on irregular terrain increased the ROM of the hip and knee in contact (*p* = 0.001 and *p* < 0.001, respectively) and aerial (*p* < 0.001 for both joints) phases. All the statistically significant differences found for the joints’ ROM were also greater than the calculated smallest worthwhile change.

### Perceptual responses

Exertional and affective responses at the end of each running trial are shown in Table 1. Compared to free running (FR and FI), overall body RPE was more strenuous in Nordic running with poles (NR and NI) (*p* = 0.02). Affective valence, in turn, was not affected by running mode (*p* = 0.34). Interestingly, affective valence was more positive during running on regular surface (FR and NR) in comparison with running on irregular surface (FI and NI) (*p* = 0.004), while overall body RPE was similar between the two terrain conditions (*p* = 0.07). There were no significative interaction effects for both exertional and affective responses (*p* = 0.28 to 0.90).

## Discussion

The aim of this study was to compare spatiotemporal, angular, coordination, and perceptual responses of running between regular (compact dust) and irregular (medium-length grass) terrains and between Nordic and free running. Our first hypothesis was partially confirmed as the individuals ran using a more flexed pattern of movement from lower limbs in the irregular terrain, but with similar self-selected speed and stride length. Also, the bilateral coordination was unaffected by the poles, even on irregular terrain. In summary, our work shows that during Nordic running compared to free running, the participants favored stability over stiffness considering the higher knee ROM during contact phase. This finding is interesting because it reinforces the role of the knee extensor muscles as a shock absorber and stabilizer, particularly in the first half of the contact phase of the step. The alteration in the knee movement is accompanied by the maintenance of the hip movement pattern, indicating a postural adjustment mediated by the knee in this condition of closed-chain intersegmental coordination, while they had similar affection despite the greater exertional response in Nordic running. Also, during running on irregular terrain compared to regular terrain the participants seemed to favor stability with greater ROM, and they indicated similar exertional response but with lower affective valence in irregular terrain.

The use of poles during self-selected speed running led to a more flexed knee technique and an increase in the feeling of pleasure, although bilateral coordination, ratings of perceived exertion, and spatiotemporal mechanics remain similar between the different terrains and use of poles. An interesting question raised in the present study was about the possible change in running technique due to the concomitant effects of more compliant and more uneven terrain. This study clearly showed that individuals adjusted their running technique to a more flexed posture. These results are in line with previous findings showing a change to a more secure technique, possibly impacting negatively on stiffness, given the relationship between knee angle at contact and leg stiffness and vertical ground reaction force (Lafortune et al. 1996; Günther and Blickhan 2002; Mesquita et al. 2023) observed previously. That is, the knee ROM during contact phase of running seems to be related to the modulation of the lower limb stiffness, supporting our interpretation that the runners of the presented study adopted a more stable and less stiff posture by increasing the knee ROM. Interestingly the perception of pleasure was reduced independent of the perceived effort, demonstrating that neural pathways related to the function of the prefrontal cortex, also called the reward system of the brain, seem to specifically affect the feeling of pleasure or satisfaction. This finding may be a future candidate to deepen the understanding on the role of affective (and coordination) responses affecting the exercise tolerance (Tempest and Parfitt 2016). One possible explanation for this phenomenon is the timing of the experiment. Possibly longer test allowing suitable adjustments of oxidative metabolism, may bring different results in the perception of effort.

### Spatiotemporal and angular parameters

While the running speed and stride length were unaffected by running type and terrain condition, the stride time was longer during Nordic running compared to free running. The longer stride time during Nordic running at self-selected running speed may be associated with a reduction in lower limb stiffness (Farley and Gonzalez 1996) caused by support of the poles on the ground concomitantly to legs. The greater knee ROM during contact phase on Nordic running also indicate that the poles enabled a reduced lower limb stiffness (Mcmahon et al. 1987).

Despite the increased support provided by the poles, the running speed was similar in free and Nordic running, even on the irregular terrain. This could be a biomechanical strategy related to the self-selected speed condition, where the assistance to dynamic stability provided by the poles may not have been as important during healthy adult running. Contrary to what happens in walking, where the self-selected speed is increased using poles (Monteiro et al. 2017), healthy young adults choose a similar self-selected running speed with a higher perceived exertion but unaltered bilateral coordination using poles.

The terrain type had no effect on the spatiotemporal responses in the current study, which could be attributed to the unevenness level of the medium length grass terrain. Perhaps greater adjustments of the spatiotemporal running responses could have been observed if a more uneven terrain was used, as longer grass in (Hébert-Losier et al. 2015). Hébert-Losier et al. compared amateur running at 3.8 m/s on three types of terrain (compact dust on cement, medium-length grass, and long-length grass), and they found that the stride length decreased only on the longest grass surface (Hébert-Losier et al. 2015).

During the running in irregular terrain, the possible adjustments on the lower-limb kinematics were either (i) the runners would increase the lower limb ROM to adopt a more flexed posture (Mcmahon et al. 1987) in favor of the postural stability (Hébert-Losier et al. 2015) to reduce the vertical body saw (Peyré-Tartaruga et al. 2021) and raise the step height (Müller and Blickhan 2010), or (ii) that they would reduce the lower limb ROM in order to position the joints in a more extended position to increase the lower limb stiffness, compensating the for the reduced stiffness of the grass (Ferris et al. 1998). We observed the first strategy, in which amateur runners increased hip and knee sagittal ROM in irregular terrain while running at their own pace, indicating that the postural stability task was prioritized over lower limb stiffness.

The use of poles had distinct effects on the ankle joint on each terrain, as the use of poles increased the ankle ROM on the regular terrain while decreased it on the irregular terrain. These findings suggest that the use of poles on the regular terrain induced a greater angular excursion of the ankle which could be associated to a higher work production by this distal joint. On the other side, the use of poles on the irregular terrain could have had a protective effect for the ankle joint considering the reduction of its angular movement while using poles for running.

### Coordination

We did not observe any modifications of the bilateral coordination and dynamic stability during running at compact dust and medium-length grass with and without Nordic poles. We expected that with the usage of poles the bilateral coordination would worsen, because of the increased motor task difficulty due to the need to synchronize the poles’ displacement with the lower limbs’ movements. However, we did not find differences for the bilateral coordination, accuracy and stability during running with and without Nordic poles, and in regular and irregular terrain. Our results suggest that the motor programs of running (Cappellini et al. 2006) are robust enough to maintain unaffected the bilateral coordination of a motor dual-task during the acute use of Nordic poles by amateur runners. Future studies can verify the longitudinal effects of the Nordic poles on coordination investigating the muscular activation—with, e.g., principal component analysis.

### Perceptual responses

The Nordic running elicited greater exertional values but the affective valence was similar compared to free running, suggesting that the Nordic running had higher physiological stress with equal positive affection than free running. The increased exertional perception during Nordic running compared to free running could have been associated to the biomechanical pattern adopted, as the participants ran with more flexed lower limbs in Nordic running, and, presumably, augmented their metabolic demand. The running on irregular terrain also had greater flexion of the lower limbs compared to running on regular terrain, however, the exertion perception was similar in both terrains. This could have been due to another non-controlled factor, considering that the affective valence was lower during running on irregular terrain. Therefore, as previously observed between Nordic and free walking (Figard-Fabre et al. 2010; Pellegrini et al. 2015, 2017; Peyré-Tartaruga et al. 2022), our results indicate that running with poles by healthy adults seems a good choice to augment the physiological stress at unaltered pleasure feeling.

### Limitations

The irregular terrain where the participants ran was a soccer field with tall grass. One interesting question concerns the level of irregularity on terrain affecting the perceptual and coordination responses. It may contribute to understand the decisions on utilization or not of poles during training and races. This study is one of the first attempt to identify the complex interactions between terrain constraints and usage of poles to better comprehend the potential of this assistive device for trial and mountain runners.

## Conclusions

The running speed, stride length and bilateral coordination were unaffected by the terrain type or the use of Nordic poles. The larger flexion of the proximal joints of hip and knee during running on irregular terrain suggests that the runners have gave priority on postural stability over lower limb stiffness in these conditions. Also, these biomechanics adjustments of the lower limb during running seems to be associated with the perceptual responses of exertion and affection valence.

### Supplementary Information

Below is the link to the electronic supplementary material.

Supplementary file1 (PDF 606 KB)

Supplementary file2 (DOC 81 KB)

## Data Availability

The datasets generated and analyzed during the current study are available in the Figshare repository (DOI: 10.6084/m9.figshare.22122782).

## Abbreviations

ANOVA: Analysis of variance
FI: Free running on irregular terrain
FR: Free running on regular terrain
GEE: Generalized estimating equations
IMU: Inertial measurement unit
NI: Nordic running with poles on irregular terrain
NR: Nordic running with poles on regular terrain
PCI: Phase coordination index
ROM: Range of joint motion
RPE: Ratings of perceived exertion
SD: Standard deviation
SPM: Statistical parametric mapping
