# Supplementary material for: Postural adjustments and perceptual responses of Nordic running: concurrent effects of poles and irregular terrain
Source: Eur J Appl Physiol. 2024 Jan 17;124(6):1733–45. doi: 10.1007/s00421-023-05397-9 (PMC11129971; doi:10.1007/s00421-023-05397-9)
Supplement: Supplementary file 1 — Supplementary file1 (PDF 606 KB) [file 421_2023_5397_MOESM1_ESM.pdf]

STATISTICAL ANALYSIS.
IVANISKI-MELLO ET AL.
POSTURAL ADJUSTMENTS AND PERCEPTUAL RESPONSES OF NORDIC RUNNING:
CONCURRENT EFFECTS OF POLES AND IRREGULAR TERRAIN

SPATIOTEMPORAL VARIABLES

```
GENLIN Velocity BY Terrain Running (ORDER=ASCENDING)
  /MODEL Terrain Running Terrain*Running INTERCEPT=YES
 DISTRIBUTION=GAMMA LINK=LOG
  /CRITERIA SCALE=MLE PCONVERGE=1E-006(ABSOLUTE) SINGULAR=1E-012 ANALYSISTYPE=3(WALD) CILEVEL=95
    LIKELIHOOD=FULL
  /EMMEANS TABLES=Terrain SCALE=ORIGINAL COMPARE=Terrain CONTRAST=PAIRWISE PADJUST=BONFERRONI
  /EMMEANS TABLES=Running SCALE=ORIGINAL COMPARE=Running CONTRAST=PAIRWISE PADJUST=BONFERRONI
  /EMMEANS TABLES=Terrain*Running SCALE=ORIGINAL COMPARE=Running*Terrain CONTRAST=PAIRWISE
    PADJUST=BONFERRONI
  /REPEATED SUBJECT=Subject SORT=YES CORRTYPE=INDEPENDENT ADJUSTCORR=YES COVB=ROBUST
  /MISSING CLASSMISSING=EXCLUDE
  /PRINT CPS DESCRIPTIVES MODELINFO FIT SUMMARY SOLUTION.
```

Generalized Linear Models

Notes

Output Created		30-OCT-2023 12:18:02
Comments		
Input	Data	C:\Users\andre\Documents\Andre\Pesquisa\Artigos para Publicar\Nordic Running\Data\Statistics\SPSS\Spatiotemporal.sav
	Active Dataset	DataSet1
	Filter	<none>
	Weight	<none>
	Split File	<none>
	N of Rows in Working Data File	49
Missing Value Handling	Definition of Missing	User-defined missing values for factor, subject and within-subject variables are treated as missing.
	Cases Used	Statistics are based on cases with valid data for all variables in the model.
Weight Handling		not applicable

Notes

Syntax		GENLIN Velocity BY Terrain Running (ORDER=ASCENDING)<br>/MODEL Terrain Running Terrain*Running INTERCEPT=YES<br>DISTRIBUTION=GAMMA LINK=LOG<br>/CRITERIA SCALE=MLE PCONVERGE=1E-006 (ABSOLUTE) SINGULAR=1E-012 ANALYSISTYPE=3 (WALD) CILEVEL=95<br>LIKELIHOOD=FULL<br>/EMMEANS TABLES=Terrain SCALE=ORIGINAL COMPARE=Terrain CONTRAST=PAIRWISE PADJUST=BONFERRONI<br>/EMMEANS TABLES=Running SCALE=ORIGINAL COMPARE=Running CONTRAST=PAIRWISE PADJUST=BONFERRONI<br>/EMMEANS TABLES=Terrain*Running SCALE=ORIGINAL COMPARE=Running*Terrain CONTRAST=PAIRWISE<br><br>PADJUST=BONFERRONI<br>/REPEATED SUBJECT=Subject SORT=YES CORRTYPE=INDEPENDENT ADJUSTCORR=YES COVB=ROBUST<br>/MISSING CLASSMISSING=EXCLUDE<br>/PRINT CPS DESCRIPTIVES MODELINFO FIT SUMMARY SOLUTION.
Resources	Processor Time	00:00:00.06
	Elapsed Time	00:00:00.07

Model Information

Dependent Variable		Velocity
Probability Distribution		Gamma
Link Function		Log
Subject Effect	1	Subject
Working Correlation Matrix Structure		Independent

Case Processing Summary

	N	Percent
Included	46	93.9%
Excluded	3	6.1%
Total	49	100.0%

Correlated Data Summary

Number of Levels	Subject Effect	Subject	13
Number of Subjects			13
Number of Measurements per Subject	Minimum		2
	Maximum		4
Correlation Matrix Dimension			4

Categorical Variable Information

			N	Percent
Factor	Terrain	Regular	26	56.5%
		Irregular	20	43.5%
		Total	46	100.0%
	Running	Free	24	52.2%
		Nordic	22	47.8%
		Total	46	100.0%

Continuous Variable Information

		N	Minimum	Maximum	Mean	Std. Deviation
Dependent Variable	Velocity	46	2.64	4.43	3.3713	.36699

Goodness of Fit[a]

	Value
Quasi Likelihood under Independence Model Criterion (QIC)[b]	8.777
Corrected Quasi Likelihood under Independence Model Criterion (QICC)[b]	8.512

Dependent Variable: Velocity
Model: (Intercept), Terrain, Running, Terrain * Running[a]

a. Information criteria are in smaller-is-better form.

b. Computed using the full log quasi-likelihood function.

Tests of Model Effects

Source	Type III		
	Wald Chi-Square	df	Sig.
(Intercept)	2843.469	1	.000
Terrain	.964	1	.326
Running	.197	1	.658
Terrain * Running	1.517	1	.218

Dependent Variable: Velocity
Model: (Intercept), Terrain, Running, Terrain * Running

Parameter Estimates

Parameter	B	Std. Error	95% Wald Confidence Interval Lower	95% Wald Confidence Interval Upper	Hypothesis Test Wald Chi-Square
(Intercept)	1.253	.0445	1.165	1.340	793.885
[Terrain=1.00]	-.058	.0350	-.127	.010	2.763
[Terrain=2.00]	0[a]	.	.	.	.
[Running=1.00]	-.035	.0358	-.105	.035	.939
[Running=2.00]	0[a]	.	.	.	.
[Terrain=1.00] * [Running=1.00]	.048	.0387	-.028	.124	1.517
[Terrain=1.00] * [Running=2.00]	0[a]	.	.	.	.
[Terrain=2.00] * [Running=1.00]	0[a]	.	.	.	.
[Terrain=2.00] * [Running=2.00]	0[a]	.	.	.	.
(Scale)	.012				

Parameter Estimates

Parameter	Hypothesis Test df	Hypothesis Test Sig.
(Intercept)	1	.000
[Terrain=1.00]	1	.096
[Terrain=2.00]	.	.
[Running=1.00]	1	.333
[Running=2.00]	.	.
[Terrain=1.00] * [Running=1.00]	1	.218
[Terrain=1.00] * [Running=2.00]	.	.
[Terrain=2.00] * [Running=1.00]	.	.
[Terrain=2.00] * [Running=2.00]	.	.
(Scale)		

Dependent Variable: Velocity
Model: (Intercept), Terrain, Running, Terrain * Running

a. Set to zero because this parameter is redundant.

Estimated Marginal Means 1: Terrain

Estimates

Terrain	Mean	Std. Error	95% Wald Confidence Interval	
			Lower	Upper
Regular	3.3231	.07734	3.1750	3.4782
Irregular	3.4392	.11476	3.2215	3.6717

Pairwise Comparisons

(I) Terrain	(J) Terrain	Mean Difference (I-J)	Std. Error	df	Bonferroni Sig.	95% Wald Confidence ... Lower
Regular	Irregular	-.1161	.11922	1	.330	-.3498
Irregular	Regular	.1161	.11922	1	.330	-.1176

Pairwise Comparisons

(I) Terrain	(J) Terrain	95% Wald Confidence ... Upper
Regular	Irregular	.1176
Irregular	Regular	.3498

Pairwise comparisons of estimated marginal means based on the original scale of dependent variable Velocity

Overall Test Results

Wald Chi-Square	df	Sig.
.948	1	.330

The Wald chi-square tests the effect of Terrain. This test is based on the linearly independent pairwise comparisons among the estimated marginal means.

Estimated Marginal Means 2: Running

Estimates

Running	Mean	Std. Error	95% Wald Confidence Interval	
			Lower	Upper
Free	3.3625	.06909	3.2297	3.5006
Nordic	3.3990	.10304	3.2030	3.6071

Pairwise Comparisons

(I) Running	(J) Running	Mean Difference (I-J)	Std. Error	df	Bonferroni Sig.	95% Wald Confidence ... Lower
Free	Nordic	-.0366	.08283	1	.659	-.1989
Nordic	Free	.0366	.08283	1	.659	-.1258

Pairwise Comparisons

(I) Running	(J) Running	95% Wald Confidence ... Upper
Free	Nordic	.1258
Nordic	Free	.1989

Pairwise comparisons of estimated marginal means based on the original scale of dependent variable Velocity

Overall Test Results

Wald Chi-Square	df	Sig.
.195	1	.659

The Wald chi-square tests the effect of Running. This test is based on the linearly independent pairwise comparisons among the estimated marginal means.

Estimated Marginal Means 3: Terrain* Running

Estimates

Terrain	Running	Mean	Std. Error	95% Wald Confidence Interval	
				Lower	Upper
Regular	Free	3.3448	.10251	3.1498	3.5519
	Nordic	3.3016	.07188	3.1637	3.4455
Irregular	Free	3.3802	.10085	3.1882	3.5837
	Nordic	3.4993	.15556	3.2073	3.8179

Pairwise Comparisons

(I) Running*Terrain	(J) Running*Terrain	Mean Difference (I-J)	Std. Error	df
[Running=1.00]*[Terrain=1.00]	[Running=1.00]*[Terrain=2.00]	-.0353	.14924	1
	[Running=2.00]*[Terrain=1.00]	.0433	.08577	1
	[Running=2.00]*[Terrain=2.00]	-.1545	.17442	1
[Running=1.00]*[Terrain=2.00]	[Running=1.00]*[Terrain=1.00]	.0353	.14924	1
	[Running=2.00]*[Terrain=1.00]	.0786	.11063	1
	[Running=2.00]*[Terrain=2.00]	-.1192	.12487	1
[Running=2.00]*[Terrain=1.00]	[Running=1.00]*[Terrain=1.00]	-.0433	.08577	1
	[Running=1.00]*[Terrain=2.00]	-.0786	.11063	1
	[Running=2.00]*[Terrain=2.00]	-.1978	.12332	1
[Running=2.00]*[Terrain=2.00]	[Running=1.00]*[Terrain=1.00]	.1545	.17442	1
	[Running=1.00]*[Terrain=2.00]	.1192	.12487	1
	[Running=2.00]*[Terrain=1.00]	.1978	.12332	1

Pairwise Comparisons

(I) Running*Terrain	(J) Running*Terrain	Bonferroni Sig.	95% Wald Confidence ... Lower
[Running=1.00]*[Terrain=1.00]	[Running=1.00]*[Terrain=2.00]	1.000	-.4291
	[Running=2.00]*[Terrain=1.00]	1.000	-.1830
	[Running=2.00]*[Terrain=2.00]	1.000	-.6147
[Running=1.00]*[Terrain=2.00]	[Running=1.00]*[Terrain=1.00]	1.000	-.3584
	[Running=2.00]*[Terrain=1.00]	1.000	-.2133
	[Running=2.00]*[Terrain=2.00]	1.000	-.4486
[Running=2.00]*[Terrain=1.00]	[Running=1.00]*[Terrain=1.00]	1.000	-.2696
	[Running=1.00]*[Terrain=2.00]	1.000	-.3705
	[Running=2.00]*[Terrain=2.00]	.653	-.5231
[Running=2.00]*[Terrain=2.00]	[Running=1.00]*[Terrain=1.00]	1.000	-.3057
	[Running=1.00]*[Terrain=2.00]	1.000	-.2103
	[Running=2.00]*[Terrain=1.00]	.653	-.1276

Pairwise Comparisons

(I) Running*Terrain	(J) Running*Terrain	95% Wald Confidence ... Upper
[Running=1.00]*[Terrain=1.00]	[Running=1.00]*[Terrain=2.00]	.3584
	[Running=2.00]*[Terrain=1.00]	.2696
	[Running=2.00]*[Terrain=2.00]	.3057
[Running=1.00]*[Terrain=2.00]	[Running=1.00]*[Terrain=1.00]	.4291
	[Running=2.00]*[Terrain=1.00]	.3705
	[Running=2.00]*[Terrain=2.00]	.2103
[Running=2.00]*[Terrain=1.00]	[Running=1.00]*[Terrain=1.00]	.1830
	[Running=1.00]*[Terrain=2.00]	.2133
	[Running=2.00]*[Terrain=2.00]	.1276
[Running=2.00]*[Terrain=2.00]	[Running=1.00]*[Terrain=1.00]	.6147
	[Running=1.00]*[Terrain=2.00]	.4486
	[Running=2.00]*[Terrain=1.00]	.5231

Pairwise comparisons of estimated marginal means based on the original scale of dependent variable Velocity

Overall Test Results

Wald Chi-Square	df	Sig.
3.977	3	.264

The Wald chi-square tests the effect of Running*Terrain. This test is based on the linearly independent pairwise comparisons among the estimated marginal means.

```
GENLIN Stride_time BY Terrain Running (ORDER=ASCENDING)
 /MODEL Terrain Running Terrain*Running INTERCEPT=YES
DISTRIBUTION=NORMAL LINK=IDENTITY
```

```
  /CRITERIA SCALE=MLE PCONVERGE=1E-006(ABSOLUTE) SINGULAR=1E-012 ANALYSISTY
PE=3(WALD) CILEVEL=95
    LIKELIHOOD=FULL
  /EMMEANS TABLES=Terrain SCALE=ORIGINAL COMPARE=Terrain CONTRAST=PAIRWISE
PADJUST=BONFERRONI
  /EMMEANS TABLES=Running SCALE=ORIGINAL COMPARE=Running CONTRAST=PAIRWISE
PADJUST=BONFERRONI
  /EMMEANS TABLES=Terrain*Running SCALE=ORIGINAL COMPARE=Running*Terrain CO
NTRAST=PAIRWISE
    PADJUST=BONFERRONI
  /REPEATED SUBJECT=Subject SORT=YES CORRTYPE=INDEPENDENT ADJUSTCORR=YES CO
VB=ROBUST
  /MISSING CLASSMISSING=EXCLUDE
  /PRINT CPS DESCRIPTIVES MODELINFO FIT SUMMARY SOLUTION.
```

Generalized Linear Models

Notes

Output Created		30-OCT-2023 12:20:46
Comments		
Input	Data	C:\Users\andre\Documents\Andre\Pesquisa\Artigos para Publicar\Nordic Running\Data\Statistics\SPSS\Spatiotemporal.sav
	Active Dataset	DataSet1
	Filter	<none>
	Weight	<none>
	Split File	<none>
	N of Rows in Working Data File	49
Missing Value Handling	Definition of Missing	User-defined missing values for factor, subject and within-subject variables are treated as missing.
	Cases Used	Statistics are based on cases with valid data for all variables in the model.
Weight Handling		not applicable

Notes

Syntax		GENLIN Stride_time BY Terrain Running (ORDER=ASCENDING)<br>/MODEL Terrain Running Terrain*Running INTERCEPT=YES<br><br>DISTRIBUTION=NORMAL LINK=IDENTITY<br>/CRITERIA SCALE=MLE PCONVERGE=1E-006 (ABSOLUTE) SINGULAR=1E-012 ANALYSISTYPE=3 (WALD) CILEVEL=95<br>LIKELIHOOD=FULL<br>/EMMEANS TABLES=Terrain SCALE=ORIGINAL COMPARE=Terrain CONTRAST=PAIRWISE PADJUST=BONFERRONI<br>/EMMEANS TABLES=Running SCALE=ORIGINAL COMPARE=Running CONTRAST=PAIRWISE PADJUST=BONFERRONI<br>/EMMEANS TABLES=Terrain*Running SCALE=ORIGINAL COMPARE=Running*Terrain CONTRAST=PAIRWISE<br><br>PADJUST=BONFERRONI<br>/REPEATED SUBJECT=Subject SORT=YES CORRTYPE=INDEPENDENT ADJUSTCORR=YES COVB=ROBUST<br>/MISSING CLASSMISSING=EXCLUDE<br>/PRINT CPS DESCRIPTIVES MODELINFO FIT SUMMARY SOLUTION.
Resources	Processor Time	00:00:00.06
	Elapsed Time	00:00:00.07

Model Information

Dependent Variable		Stride_time
Probability Distribution		Normal
Link Function		Identity
Subject Effect	1	Subject
Working Correlation Matrix Structure		Independent

Case Processing Summary

	N	Percent
Included	48	98.0%
Excluded	1	2.0%
Total	49	100.0%

Correlated Data Summary

Number of Levels	Subject Effect	Subject	13
Number of Subjects			13
Number of Measurements per Subject	Minimum		2
	Maximum		4
Correlation Matrix Dimension			4

Categorical Variable Information

			N	Percent
Factor	Terrain	Regular	26	54.2%
		Irregular	22	45.8%
		Total	48	100.0%
	Running	Free	24	50.0%
		Nordic	24	50.0%
		Total	48	100.0%

Continuous Variable Information

		N	Minimum	Maximum	Mean	Std. Deviation
Dependent Variable	Stride_time	48	.67	.85	.7582	.04314

Goodness of Fit[a]

	Value
Quasi Likelihood under Independence Model Criterion (QIC)[b]	8.128
Corrected Quasi Likelihood under Independence Model Criterion (QICC)[b]	8.079

Dependent Variable: Stride_time
Model: (Intercept), Terrain, Running, Terrain * Running[a]

a. Information criteria are in smaller-is-better form.

b. Computed using the full log quasi-likelihood function.

Tests of Model Effects

Source	Type III		
	Wald Chi-Square	df	Sig.
(Intercept)	5515.520	1	.000
Terrain	.569	1	.451
Running	16.942	1	.000
Terrain * Running	2.963	1	.085

Dependent Variable: Stride_time
Model: (Intercept), Terrain, Running, Terrain * Running

Parameter Estimates

Parameter	B	Std. Error	95% Wald Confidence Interval		Hypothesis Test
			Lower	Upper	Wald Chi-Square
(Intercept)	.771	.0144	.743	.800	2872.667
[Terrain=1.00]	-.002	.0123	-.026	.022	.020
[Terrain=2.00]	0[a]	.	.	.	.
[Running=1.00]	-.018	.0074	-.033	-.004	5.977
[Running=2.00]	0[a]	.	.	.	.
[Terrain=1.00] * [Running=1.00]	-.012	.0067	-.025	.002	2.963
[Terrain=1.00] * [Running=2.00]	0[a]	.	.	.	.
[Terrain=2.00] * [Running=1.00]	0[a]	.	.	.	.
[Terrain=2.00] * [Running=2.00]	0[a]	.	.	.	.
(Scale)	.002				

Parameter Estimates

Parameter	Hypothesis Test	
	df	Sig.
(Intercept)	1	.000
[Terrain=1.00]	1	.888
[Terrain=2.00]	.	.
[Running=1.00]	1	.014
[Running=2.00]	.	.
[Terrain=1.00] * [Running=1.00]	1	.085
[Terrain=1.00] * [Running=2.00]	.	.
[Terrain=2.00] * [Running=1.00]	.	.
[Terrain=2.00] * [Running=2.00]	.	.
(Scale)		

Dependent Variable: Stride_time
Model: (Intercept), Terrain, Running, Terrain * Running

a. Set to zero because this parameter is redundant.

Estimated Marginal Means 1: Terrain

Estimates

Terrain	Mean	Std. Error	95% Wald Confidence Interval	
			Lower	Upper
Regular	.7548	.01048	.7342	.7753
Irregular	.7623	.01217	.7384	.7861

Pairwise Comparisons

(I) Terrain	(J) Terrain	Mean Difference (I-J)	Std. Error	df	Bonferroni Sig.	95% Wald Confidence ... Lower
Regular	Irregular	-.0075	.00993	1	.451	-.0270
Irregular	Regular	.0075	.00993	1	.451	-.0120

Pairwise Comparisons

(I) Terrain	(J) Terrain	95% Wald Confidence ... Upper
Regular	Irregular	.0120
Irregular	Regular	.0270

Pairwise comparisons of estimated marginal means based on the original scale of dependent variable Stride_time

Overall Test Results

Wald Chi-Square	df	Sig.
.569	1	.451

The Wald chi-square tests the effect of Terrain. This test is based on the linearly independent pairwise comparisons among the estimated marginal means.

Estimated Marginal Means 2: Running

Estimates

Running	Mean	Std. Error	95% Wald Confidence Interval	
			Lower	Upper
Free	.7466	.00951	.7279	.7652
Nordic	.7705	.01162	.7477	.7933

Pairwise Comparisons

(I) Running	(J) Running	Mean Difference (I-J)	Std. Error	df	Bonferroni Sig.	95% Wald Confidence ... Lower
Free	Nordic	-.0239[a]	.00582	1	.000	-.0353
Nordic	Free	.0239[a]	.00582	1	.000	.0125

Pairwise Comparisons

(I) Running	(J) Running	95% Wald Confidence ... Upper
Free	Nordic	-.0125
Nordic	Free	.0353

Pairwise comparisons of estimated marginal means based on the original scale of dependent variable Stride_time

a. The mean difference is significant at the .05 level.

Overall Test Results

Wald Chi-Square	df	Sig.
16.942	1	.000

The Wald chi-square tests the effect of Running. This test is based on the linearly independent pairwise comparisons among the estimated marginal means.

Estimated Marginal Means 3: Terrain* Running

Estimates

Terrain	Running	Mean	Std. Error	95% Wald Confidence Interval Lower	95% Wald Confidence Interval Upper
Regular	Free	.7399	.00993	.7205	.7594
	Nordic	.7696	.01176	.7466	.7927
Irregular	Free	.7532	.01081	.7320	.7744
	Nordic	.7714	.01439	.7432	.7996

Pairwise Comparisons

(I) Running*Terrain	(J) Running*Terrain	Mean Difference (I-J)	Std. Error	df
[Running=1.00]*[Terrain=1.00]	[Running=1.00]*[Terrain=2.00]	-.0133	.00832	1
	[Running=2.00]*[Terrain=1.00]	-.0297[a]	.00590	1
	[Running=2.00]*[Terrain=2.00]	-.0314[a]	.01133	1
[Running=1.00]*[Terrain=2.00]	[Running=1.00]*[Terrain=1.00]	.0133	.00832	1
	[Running=2.00]*[Terrain=1.00]	-.0165	.01169	1
	[Running=2.00]*[Terrain=2.00]	-.0182	.00744	1
[Running=2.00]*[Terrain=1.00]	[Running=1.00]*[Terrain=1.00]	.0297[a]	.00590	1
	[Running=1.00]*[Terrain=2.00]	.0165	.01169	1
	[Running=2.00]*[Terrain=2.00]	-.0017	.01227	1
[Running=2.00]*[Terrain=2.00]	[Running=1.00]*[Terrain=1.00]	.0314[a]	.01133	1
	[Running=1.00]*[Terrain=2.00]	.0182	.00744	1
	[Running=2.00]*[Terrain=1.00]	.0017	.01227	1

Pairwise Comparisons

(I) Running*Terrain	(J) Running*Terrain	Bonferroni Sig.	95% Wald Confidence ...
			Lower
[Running=1.00]*[Terrain=1.00]	[Running=1.00]*[Terrain=2.00]	.666	-.0352
	[Running=2.00]*[Terrain=1.00]	.000	-.0453
	[Running=2.00]*[Terrain=2.00]	.033	-.0613
[Running=1.00]*[Terrain=2.00]	[Running=1.00]*[Terrain=1.00]	.666	-.0087
	[Running=2.00]*[Terrain=1.00]	.955	-.0473
	[Running=2.00]*[Terrain=2.00]	.087	-.0378
[Running=2.00]*[Terrain=1.00]	[Running=1.00]*[Terrain=1.00]	.000	.0141
	[Running=1.00]*[Terrain=2.00]	.955	-.0144
	[Running=2.00]*[Terrain=2.00]	1.000	-.0341
[Running=2.00]*[Terrain=2.00]	[Running=1.00]*[Terrain=1.00]	.033	.0016
	[Running=1.00]*[Terrain=2.00]	.087	-.0014
	[Running=2.00]*[Terrain=1.00]	1.000	-.0306

Pairwise Comparisons

(I) Running*Terrain	(J) Running*Terrain	95% Wald Confidence ... Upper
[Running=1.00]*[Terrain=1.00]	[Running=1.00]*[Terrain=2.00]	.0087
	[Running=2.00]*[Terrain=1.00]	-.0141
	[Running=2.00]*[Terrain=2.00]	-.0016
[Running=1.00]*[Terrain=2.00]	[Running=1.00]*[Terrain=1.00]	.0352
	[Running=2.00]*[Terrain=1.00]	.0144
	[Running=2.00]*[Terrain=2.00]	.0014
[Running=2.00]*[Terrain=1.00]	[Running=1.00]*[Terrain=1.00]	.0453
	[Running=1.00]*[Terrain=2.00]	.0473
	[Running=2.00]*[Terrain=2.00]	.0306
[Running=2.00]*[Terrain=2.00]	[Running=1.00]*[Terrain=1.00]	.0613
	[Running=1.00]*[Terrain=2.00]	.0378
	[Running=2.00]*[Terrain=1.00]	.0341

Pairwise comparisons of estimated marginal means based on the original scale of dependent variable Stride_time

a. The mean difference is significant at the .05 level.

Overall Test Results

Wald Chi-Square	df	Sig.
38.399	3	.000

The Wald chi-square tests the effect of Running*Terrain. This test is based on the linearly independent pairwise comparisons among the estimated marginal means.

```
GENLIN Stride_length BY Terrain Running (ORDER=ASCENDING)
 /MODEL Terrain Running Terrain*Running INTERCEPT=YES
```

```
 DISTRIBUTION=GAMMA LINK=LOG
  /CRITERIA SCALE=MLE PCONVERGE=1E-006(ABSOLUTE) SINGULAR=1E-012 ANALYSISTY
PE=3(WALD) CILEVEL=95
    LIKELIHOOD=FULL
  /EMMEANS TABLES=Terrain SCALE=ORIGINAL COMPARE=Terrain CONTRAST=PAIRWISE
PADJUST=BONFERRONI
  /EMMEANS TABLES=Running SCALE=ORIGINAL COMPARE=Running CONTRAST=PAIRWISE
PADJUST=BONFERRONI
  /EMMEANS TABLES=Terrain*Running SCALE=ORIGINAL COMPARE=Running*Terrain CO
NTRAST=PAIRWISE
    PADJUST=BONFERRONI
  /REPEATED SUBJECT=Subject SORT=YES CORRTYPE=INDEPENDENT ADJUSTCORR=YES CO
VB=ROBUST
  /MISSING CLASSMISSING=EXCLUDE
  /PRINT CPS DESCRIPTIVES MODELINFO FIT SUMMARY SOLUTION.
```

Generalized Linear Models

Notes

Output Created		30-OCT-2023 12:22:10
Comments		
Input	Data	C: \Users\andre\Documents\Andre\Pesquisa\Artigos para Publicar\Nordic Running\Data\Statistics\SPSS\Spatiotemporal.sav
	Active Dataset	DataSet1
	Filter	<none>
	Weight	<none>
	Split File	<none>
	N of Rows in Working Data File	49
Missing Value Handling	Definition of Missing	User-defined missing values for factor, subject and within-subject variables are treated as missing.
	Cases Used	Statistics are based on cases with valid data for all variables in the model.
Weight Handling		not applicable

Notes

Syntax		GENLIN Stride_length BY Terrain Running (ORDER=ASCENDING)<br>/MODEL Terrain Running Terrain*Running INTERCEPT=YES<br>DISTRIBUTION=GAMMA LINK=LOG<br>/CRITERIA SCALE=MLE PCONVERGE=1E-006 (ABSOLUTE) SINGULAR=1E-012 ANALYSISTYPE=3 (WALD) CILEVEL=95<br>LIKELIHOOD=FULL<br>/EMMEANS TABLES=Terrain SCALE=ORIGINAL COMPARE=Terrain CONTRAST=PAIRWISE PADJUST=BONFERRONI<br>/EMMEANS TABLES=Running SCALE=ORIGINAL COMPARE=Running CONTRAST=PAIRWISE PADJUST=BONFERRONI<br>/EMMEANS TABLES=Terrain*Running SCALE=ORIGINAL COMPARE=Running*Terrain CONTRAST=PAIRWISE<br><br>PADJUST=BONFERRONI<br>/REPEATED SUBJECT=Subject SORT=YES CORRTYPE=INDEPENDENT ADJUSTCORR=YES COVB=ROBUST<br>/MISSING CLASSMISSING=EXCLUDE<br>/PRINT CPS DESCRIPTIVES MODELINFO FIT SUMMARY SOLUTION.
Resources	Processor Time	00:00:00.05
	Elapsed Time	00:00:00.05

Model Information

Dependent Variable		Stride_length
Probability Distribution		Gamma
Link Function		Log
Subject Effect	1	Subject
Working Correlation Matrix Structure		Independent

Case Processing Summary

	N	Percent
Included	46	93.9%
Excluded	3	6.1%
Total	49	100.0%

Correlated Data Summary

Number of Levels	Subject Effect	Subject	13
Number of Subjects			13
Number of Measurements per Subject	Minimum		2
	Maximum		4
Correlation Matrix Dimension			4

Categorical Variable Information

			N	Percent
Factor	Terrain	Regular	26	56.5%
		Irregular	20	43.5%
		Total	46	100.0%
	Running	Free	24	52.2%
		Nordic	22	47.8%
		Total	46	100.0%

Continuous Variable Information

		N	Minimum	Maximum	Mean	Std. Deviation
Dependent Variable	Stride_length	46	1.99	3.37	2.5430	.26680

Goodness of Fit[a]

	Value
Quasi Likelihood under Independence Model Criterion (QIC)[b]	8.825
Corrected Quasi Likelihood under Independence Model Criterion (QICC)[b]	8.464

Dependent Variable: Stride_length
Model: (Intercept), Terrain, Running, Terrain * Running[a]

a. Information criteria are in smaller-is-better form.

b. Computed using the full log quasi-likelihood function.

Tests of Model Effects

	Type III		
Source	Wald Chi-Square	df	Sig.
(Intercept)	1678.782	1	.000
Terrain	1.362	1	.243
Running	2.600	1	.107
Terrain * Running	.269	1	.604

Dependent Variable: Stride_length
Model: (Intercept), Terrain, Running, Terrain * Running

Parameter Estimates

Parameter	B	Std. Error	95% Wald Confidence Interval Lower	Upper	Hypothesis Test Wald Chi-Square
(Intercept)	.978	.0423	.895	1.061	533.571
[Terrain=1.00]	-.047	.0320	-.110	.015	2.201
[Terrain=2.00]	0[a]	.	.	.	.
[Running=1.00]	-.044	.0290	-.101	.013	2.266
[Running=2.00]	0[a]	.	.	.	.
[Terrain=1.00] * [Running=1.00]	.017	.0330	-.048	.082	.269
[Terrain=1.00] * [Running=2.00]	0[a]	.	.	.	.
[Terrain=2.00] * [Running=1.00]	0[a]	.	.	.	.
[Terrain=2.00] * [Running=2.00]	0[a]	.	.	.	.
(Scale)	.011				

Parameter Estimates

Parameter	Hypothesis Test df	Sig.
(Intercept)	1	.000
[Terrain=1.00]	1	.138
[Terrain=2.00]	.	.
[Running=1.00]	1	.132
[Running=2.00]	.	.
[Terrain=1.00] * [Running=1.00]	1	.604
[Terrain=1.00] * [Running=2.00]	.	.
[Terrain=2.00] * [Running=1.00]	.	.
[Terrain=2.00] * [Running=2.00]	.	.
(Scale)		

Dependent Variable: Stride_length
Model: (Intercept), Terrain, Running, Terrain * Running

a. Set to zero because this parameter is redundant.

Estimated Marginal Means 1: Terrain

Estimates

Terrain	Mean	Std. Error	95% Wald Confidence Interval Lower	95% Wald Confidence Interval Upper
Regular	2.5019	.04943	2.4069	2.6007
Irregular	2.6012	.09051	2.4297	2.7848

Pairwise Comparisons

(I) Terrain	(J) Terrain	Mean Difference (I-J)	Std. Error	df	Bonferroni Sig.	95% Wald Confidence ... Lower
Regular	Irregular	-.0993	.08634	1	.250	-.2685
Irregular	Regular	.0993	.08634	1	.250	-.0699

Pairwise Comparisons

(I) Terrain	(J) Terrain	95% Wald Confidence ... Upper
Regular	Irregular	.0699
Irregular	Regular	.2685

Pairwise comparisons of estimated marginal means based on the original scale of dependent variable Stride_length

Overall Test Results

Wald Chi-Square	df	Sig.
1.323	1	.250

The Wald chi-square tests the effect of Terrain. This test is based on the linearly independent pairwise comparisons among the estimated marginal means.

Estimated Marginal Means 2: Running

Estimates

Running	Mean	Std. Error	95% Wald Confidence Interval Lower	95% Wald Confidence Interval Upper
Free	2.5066	.05346	2.4040	2.6136
Nordic	2.5963	.07469	2.4540	2.7469

Pairwise Comparisons

(I) Running	(J) Running	Mean Difference (I-J)	Std. Error	df	Bonferroni Sig.	95% Wald Confidence ... Lower
Free	Nordic	-.0897	.05641	1	.112	-.2002
Nordic	Free	.0897	.05641	1	.112	-.0209

Pairwise Comparisons

(I) Running	(J) Running	95% Wald Confidence ... Upper
Free	Nordic	.0209
Nordic	Free	.2002

Pairwise comparisons of estimated marginal means based on the original scale of dependent variable Stride_length

Overall Test Results

Wald Chi-Square	df	Sig.
2.526	1	.112

The Wald chi-square tests the effect of Running. This test is based on the linearly independent pairwise comparisons among the estimated marginal means.

Estimated Marginal Means 3: Terrain* Running

Estimates

Terrain	Running	Mean	Std. Error	95% Wald Confidence Interval Lower	95% Wald Confidence Interval Upper
Regular	Free	2.4689	.06677	2.3414	2.6032
	Nordic	2.5354	.04911	2.4409	2.6335
Irregular	Free	2.5450	.08249	2.3884	2.7119
	Nordic	2.6587	.11255	2.4470	2.8887

Pairwise Comparisons

(I) Running*Terrain	(J) Running*Terrain	Mean Difference (I-J)	Std. Error	df
[Running=1.00]*[Terrain=1.00]	[Running=1.00]*[Terrain=2.00]	-.0762	.10501	1
	[Running=2.00]*[Terrain=1.00]	-.0665	.06342	1
	[Running=2.00]*[Terrain=2.00]	-.1898	.12374	1
[Running=1.00]*[Terrain=2.00]	[Running=1.00]*[Terrain=1.00]	.0762	.10501	1
	[Running=2.00]*[Terrain=1.00]	.0096	.07747	1
	[Running=2.00]*[Terrain=2.00]	-.1137	.07706	1
[Running=2.00]*[Terrain=1.00]	[Running=1.00]*[Terrain=1.00]	.0665	.06342	1
	[Running=1.00]*[Terrain=2.00]	-.0096	.07747	1
	[Running=2.00]*[Terrain=2.00]	-.1233	.08587	1
[Running=2.00]*[Terrain=2.00]	[Running=1.00]*[Terrain=1.00]	.1898	.12374	1
	[Running=1.00]*[Terrain=2.00]	.1137	.07706	1
	[Running=2.00]*[Terrain=1.00]	.1233	.08587	1

Pairwise Comparisons

(I) Running*Terrain	(J) Running*Terrain	Bonferroni Sig.	95% Wald Confidence ... Lower
[Running=1.00]*[Terrain=1.00]	[Running=1.00]*[Terrain=2.00]	1.000	-.3532
	[Running=2.00]*[Terrain=1.00]	1.000	-.2339
	[Running=2.00]*[Terrain=2.00]	.750	-.5163
[Running=1.00]*[Terrain=2.00]	[Running=1.00]*[Terrain=1.00]	1.000	-.2009
	[Running=2.00]*[Terrain=1.00]	1.000	-.1948
	[Running=2.00]*[Terrain=2.00]	.841	-.3170
[Running=2.00]*[Terrain=1.00]	[Running=1.00]*[Terrain=1.00]	1.000	-.1008
	[Running=1.00]*[Terrain=2.00]	1.000	-.2140
	[Running=2.00]*[Terrain=2.00]	.907	-.3498
[Running=2.00]*[Terrain=2.00]	[Running=1.00]*[Terrain=1.00]	.750	-.1367
	[Running=1.00]*[Terrain=2.00]	.841	-.0896
	[Running=2.00]*[Terrain=1.00]	.907	-.1033

Pairwise Comparisons

(I) Running*Terrain	(J) Running*Terrain	95% Wald Confidence ... Upper
[Running=1.00]*[Terrain=1.00]	[Running=1.00]*[Terrain=2.00]	.2009
	[Running=2.00]*[Terrain=1.00]	.1008
	[Running=2.00]*[Terrain=2.00]	.1367
[Running=1.00]*[Terrain=2.00]	[Running=1.00]*[Terrain=1.00]	.3532
	[Running=2.00]*[Terrain=1.00]	.2140
	[Running=2.00]*[Terrain=2.00]	.0896
[Running=2.00]*[Terrain=1.00]	[Running=1.00]*[Terrain=1.00]	.2339
	[Running=1.00]*[Terrain=2.00]	.1948
	[Running=2.00]*[Terrain=2.00]	.1033
[Running=2.00]*[Terrain=2.00]	[Running=1.00]*[Terrain=1.00]	.5163
	[Running=1.00]*[Terrain=2.00]	.3170
	[Running=2.00]*[Terrain=1.00]	.3498

Pairwise comparisons of estimated marginal means based on the original scale of dependent variable Stride_length

Overall Test Results

Wald Chi-Square	df	Sig.
2.951	3	.399

The Wald chi-square tests the effect of Running*Terrain. This test is based on the linearly independent pairwise comparisons among the estimated marginal means.

```
GENLIN Stance_phase_Right BY Terrain Running (ORDER=ASCENDING)
 /MODEL Terrain Running Terrain*Running INTERCEPT=YES
DISTRIBUTION=GAMMA LINK=LOG
```

```
  /CRITERIA SCALE=MLE PCONVERGE=1E-006(ABSOLUTE) SINGULAR=1E-012 ANALYSISTY
PE=3(WALD) CILEVEL=95
    LIKELIHOOD=FULL
  /EMMEANS TABLES=Terrain SCALE=ORIGINAL COMPARE=Terrain CONTRAST=PAIRWISE
PADJUST=BONFERRONI
  /EMMEANS TABLES=Running SCALE=ORIGINAL COMPARE=Running CONTRAST=PAIRWISE
PADJUST=BONFERRONI
  /EMMEANS TABLES=Terrain*Running SCALE=ORIGINAL COMPARE=Running*Terrain CO
NTRAST=PAIRWISE
    PADJUST=BONFERRONI
  /REPEATED SUBJECT=Subject SORT=YES CORRTYPE=INDEPENDENT ADJUSTCORR=YES CO
VB=ROBUST
  /MISSING CLASSMISSING=EXCLUDE
  /PRINT CPS DESCRIPTIVES MODELINFO FIT SUMMARY SOLUTION.
```

Generalized Linear Models

Notes

Output Created		30-OCT-2023 12:24:28
Comments		
Input	Data	C:\Users\andre\Documents\Andre\Pesquisa\Artigos para Publicar\Nordic Running\Data\Statistics\SPSS\Spatiotemporal.sav
	Active Dataset	DataSet1
	Filter	<none>
	Weight	<none>
	Split File	<none>
	N of Rows in Working Data File	49
Missing Value Handling	Definition of Missing	User-defined missing values for factor, subject and within-subject variables are treated as missing.
	Cases Used	Statistics are based on cases with valid data for all variables in the model.
Weight Handling		not applicable

Notes

Syntax		GENLIN Stance_phase_Right BY Terrain Running (ORDER=ASCENDING)<br>/MODEL Terrain Running Terrain*Running INTERCEPT=YES<br>DISTRIBUTION=GAMMA LINK=LOG<br>/CRITERIA SCALE=MLE PCONVERGE=1E-006 (ABSOLUTE) SINGULAR=1E-012 ANALYSISTYPE=3 (WALD) CILEVEL=95<br>LIKELIHOOD=FULL<br>/EMMEANS TABLES=Terrain SCALE=ORIGINAL COMPARE=Terrain CONTRAST=PAIRWISE PADJUST=BONFERRONI<br>/EMMEANS TABLES=Running SCALE=ORIGINAL COMPARE=Running CONTRAST=PAIRWISE PADJUST=BONFERRONI<br>/EMMEANS TABLES=Terrain*Running SCALE=ORIGINAL COMPARE=Running*Terrain CONTRAST=PAIRWISE<br><br>PADJUST=BONFERRONI<br>/REPEATED SUBJECT=Subject SORT=YES CORRTYPE=INDEPENDENT ADJUSTCORR=YES COVB=ROBUST<br>/MISSING CLASSMISSING=EXCLUDE<br>/PRINT CPS DESCRIPTIVES MODELINFO FIT SUMMARY SOLUTION.
Resources	Processor Time	00:00:00.06
	Elapsed Time	00:00:00.07

Model Information

Dependent Variable	Stance_phase_Right
Probability Distribution	Gamma
Link Function	Log
Subject Effect 1	Subject
Working Correlation Matrix Structure	Independent

Case Processing Summary

	N	Percent
Included	48	98.0%
Excluded	1	2.0%
Total	49	100.0%

Correlated Data Summary

Number of Levels	Subject Effect	Subject	13
Number of Subjects			13
Number of Measurements per Subject	Minimum		2
	Maximum		4
Correlation Matrix Dimension			4

Categorical Variable Information

			N	Percent
Factor	Terrain	Regular	26	54.2%
		Irregular	22	45.8%
		Total	48	100.0%
	Running	Free	24	50.0%
		Nordic	24	50.0%
		Total	48	100.0%

Continuous Variable Information

		N	Minimum	Maximum	Mean
Dependent Variable	Stance_phase_Right	48	24.67	37.63	32.1414

Continuous Variable Information

		Std. Deviation
Dependent Variable	Stance_phase_Right	2.83687

Goodness of Fit[a]

	Value
Quasi Likelihood under Independence Model Criterion (QIC)[b]	8.317
Corrected Quasi Likelihood under Independence Model Criterion (QICC)[b]	8.366

Dependent Variable: Stance_phase_Right
Model: (Intercept), Terrain, Running, Terrain * Running[a]

a. Information criteria are in smaller-is-better form.

b. Computed using the full log quasi-likelihood function.

Tests of Model Effects

Source	Type III		
	Wald Chi-Square	df	Sig.
(Intercept)	23996.944	1	.000
Terrain	2.133	1	.144
Running	.169	1	.681
Terrain * Running	.407	1	.523

Dependent Variable: Stance_phase_Right
Model: (Intercept), Terrain, Running, Terrain * Running

Parameter Estimates

Parameter	B	Std. Error	95% Wald Confidence Interval Lower	Upper	Hypothesis Test Wald Chi-Square
(Intercept)	3.453	.0265	3.401	3.504	17035.563
[Terrain=1.00]	.027	.0176	-.007	.061	2.364
[Terrain=2.00]	0[a]	.	.	.	.
[Running=1.00]	.013	.0176	-.021	.048	.557
[Running=2.00]	0[a]	.	.	.	.
[Terrain=1.00] * [Running=1.00]	-.013	.0210	-.055	.028	.407
[Terrain=1.00] * [Running=2.00]	0[a]	.	.	.	.
[Terrain=2.00] * [Running=1.00]	0[a]	.	.	.	.
[Terrain=2.00] * [Running=2.00]	0[a]	.	.	.	.
(Scale)	.008				

Parameter Estimates

Parameter	Hypothesis Test df	Sig.
(Intercept)	1	.000
[Terrain=1.00]	1	.124
[Terrain=2.00]	.	.
[Running=1.00]	1	.456
[Running=2.00]	.	.
[Terrain=1.00] * [Running=1.00]	1	.523
[Terrain=1.00] * [Running=2.00]	.	.
[Terrain=2.00] * [Running=1.00]	.	.
[Terrain=2.00] * [Running=2.00]	.	.
(Scale)		

Dependent Variable: Stance_phase_Right
Model: (Intercept), Terrain, Running, Terrain * Running

a. Set to zero because this parameter is redundant.

Estimated Marginal Means 1: Terrain

Estimates

Terrain	Mean	Std. Error	95% Wald Confidence Interval Lower	95% Wald Confidence Interval Upper
Regular	32.4399	.67735	31.1391	33.7950
Irregular	31.7879	.81895	30.2226	33.4342

Pairwise Comparisons

(I) Terrain	(J) Terrain	Mean Difference (I-J)	Std. Error	df	Bonferroni Sig.	95% Wald Confidence ... Lower
Regular	Irregular	.6520	.44130	1	.140	-.2129
Irregular	Regular	-.6520	.44130	1	.140	-1.5169

Pairwise Comparisons

(I) Terrain	(J) Terrain	95% Wald Confidence ... Upper
Regular	Irregular	1.5169
Irregular	Regular	.2129

Pairwise comparisons of estimated marginal means based on the original scale of dependent variable Stance_phase_Right

Overall Test Results

Wald Chi-Square	df	Sig.
2.183	1	.140

The Wald chi-square tests the effect of Terrain. This test is based on the linearly independent pairwise comparisons among the estimated marginal means.

Estimated Marginal Means 2: Running

Estimates

Running	Mean	Std. Error	95% Wald Confidence Interval Lower	95% Wald Confidence Interval Upper
Free	32.2159	.72629	30.8234	33.6713
Nordic	32.0089	.79557	30.4870	33.6068

Pairwise Comparisons

(I) Running	(J) Running	Mean Difference (I-J)	Std. Error	df	Bonferroni Sig.	95% Wald Confidence ... Lower
Free	Nordic	.2070	.50278	1	.681	-.7784
Nordic	Free	-.2070	.50278	1	.681	-1.1924

Pairwise Comparisons

(I) Running	(J) Running	95% Wald Confidence ... Upper
Free	Nordic	1.1924
Nordic	Free	.7784

Pairwise comparisons of estimated marginal means based on the original scale of dependent variable Stance_phase_Right

Overall Test Results

Wald Chi-Square	df	Sig.
.170	1	.681

The Wald chi-square tests the effect of Running. This test is based on the linearly independent pairwise comparisons among the estimated marginal means.

Estimated Marginal Means 3: Terrain* Running

Estimates

Terrain	Running	Mean	Std. Error	95% Wald Confidence Interval	
				Lower	Upper
Regular	Free	32.4358	.63464	31.2155	33.7039
	Nordic	32.4439	.85225	30.8158	34.1581
Irregular	Free	31.9974	.89527	30.2900	33.8011
	Nordic	31.5797	.83534	29.9842	33.2601

Pairwise Comparisons

(I) Running*Terrain	(J) Running*Terrain	Mean Difference (I-J)	Std. Error	df
[Running=1.00]*[Terrain=1.00]	[Running=1.00]*[Terrain=2.00]	.4384	.55146	1
	[Running=2.00]*[Terrain=1.00]	-.0081	.65029	1
	[Running=2.00]*[Terrain=2.00]	.8562	.72996	1
[Running=1.00]*[Terrain=2.00]	[Running=1.00]*[Terrain=1.00]	-.4384	.55146	1
	[Running=2.00]*[Terrain=1.00]	-.4465	.60168	1
	[Running=2.00]*[Terrain=2.00]	.4178	.56093	1
[Running=2.00]*[Terrain=1.00]	[Running=1.00]*[Terrain=1.00]	.0081	.65029	1
	[Running=1.00]*[Terrain=2.00]	.4465	.60168	1
	[Running=2.00]*[Terrain=2.00]	.8643	.56236	1
[Running=2.00]*[Terrain=2.00]	[Running=1.00]*[Terrain=1.00]	-.8562	.72996	1
	[Running=1.00]*[Terrain=2.00]	-.4178	.56093	1
	[Running=2.00]*[Terrain=1.00]	-.8643	.56236	1

Pairwise Comparisons

(I) Running*Terrain	(J) Running*Terrain	Bonferroni Sig.	95% Wald Confidence ... Lower
[Running=1.00]*[Terrain=1.00]	[Running=1.00]*[Terrain=2.00]	1.000	-1.0165
	[Running=2.00]*[Terrain=1.00]	1.000	-1.7237
	[Running=2.00]*[Terrain=2.00]	1.000	-1.0696
[Running=1.00]*[Terrain=2.00]	[Running=1.00]*[Terrain=1.00]	1.000	-1.8933
	[Running=2.00]*[Terrain=1.00]	1.000	-2.0339
	[Running=2.00]*[Terrain=2.00]	1.000	-1.0621
[Running=2.00]*[Terrain=1.00]	[Running=1.00]*[Terrain=1.00]	1.000	-1.7075
	[Running=1.00]*[Terrain=2.00]	1.000	-1.1409
	[Running=2.00]*[Terrain=2.00]	.746	-.6194
[Running=2.00]*[Terrain=2.00]	[Running=1.00]*[Terrain=1.00]	1.000	-2.7820
	[Running=1.00]*[Terrain=2.00]	1.000	-1.8976
	[Running=2.00]*[Terrain=1.00]	.746	-2.3479

Pairwise Comparisons

(I) Running*Terrain	(J) Running*Terrain	95% Wald Confidence ... Upper
[Running=1.00]*[Terrain=1.00]	[Running=1.00]*[Terrain=2.00]	1.8933
	[Running=2.00]*[Terrain=1.00]	1.7075
	[Running=2.00]*[Terrain=2.00]	2.7820
[Running=1.00]*[Terrain=2.00]	[Running=1.00]*[Terrain=1.00]	1.0165
	[Running=2.00]*[Terrain=1.00]	1.1409
	[Running=2.00]*[Terrain=2.00]	1.8976
[Running=2.00]*[Terrain=1.00]	[Running=1.00]*[Terrain=1.00]	1.7237
	[Running=1.00]*[Terrain=2.00]	2.0339
	[Running=2.00]*[Terrain=2.00]	2.3479
[Running=2.00]*[Terrain=2.00]	[Running=1.00]*[Terrain=1.00]	1.0696
	[Running=1.00]*[Terrain=2.00]	1.0621
	[Running=2.00]*[Terrain=1.00]	.6194

Pairwise comparisons of estimated marginal means based on the original scale of dependent variable Stance_phase_Right

Overall Test Results

Wald Chi-Square	df	Sig.
2.589	3	.459

The Wald chi-square tests the effect of Running*Terrain. This test is based on the linearly independent pairwise comparisons among the estimated marginal means.

```
GENLIN Swing_phase_Right BY Terrain Running (ORDER=ASCENDING)
 /MODEL Terrain Running Terrain*Running INTERCEPT=YES
DISTRIBUTION=GAMMA LINK=LOG
```

```
  /CRITERIA SCALE=MLE PCONVERGE=1E-006(ABSOLUTE) SINGULAR=1E-012 ANALYSISTY
PE=3(WALD) CILEVEL=95
    LIKELIHOOD=FULL
  /EMMEANS TABLES=Terrain SCALE=ORIGINAL COMPARE=Terrain CONTRAST=PAIRWISE
PADJUST=BONFERRONI
  /EMMEANS TABLES=Running SCALE=ORIGINAL COMPARE=Running CONTRAST=PAIRWISE
PADJUST=BONFERRONI
  /EMMEANS TABLES=Terrain*Running SCALE=ORIGINAL COMPARE=Running*Terrain CO
NTRAST=PAIRWISE
    PADJUST=BONFERRONI
  /REPEATED SUBJECT=Subject SORT=YES CORRTYPE=INDEPENDENT ADJUSTCORR=YES CO
VB=ROBUST
  /MISSING CLASSMISSING=EXCLUDE
  /PRINT CPS DESCRIPTIVES MODELINFO FIT SUMMARY SOLUTION.
```

Generalized Linear Models

Notes

Output Created		30-OCT-2023 12:25:38
Comments		
Input	Data	C:\Users\andre\Documents\Andre\Pesquisa\Artigos para Publicar\Nordic Running\Data\Statistics\SPSS\Spatiotemporal.sav
	Active Dataset	DataSet1
	Filter	<none>
	Weight	<none>
	Split File	<none>
	N of Rows in Working Data File	49
Missing Value Handling	Definition of Missing	User-defined missing values for factor, subject and within-subject variables are treated as missing.
	Cases Used	Statistics are based on cases with valid data for all variables in the model.
Weight Handling		not applicable

Notes

Syntax		GENLIN Swing_phase_Right BY Terrain Running (ORDER=ASCENDING)<br>/MODEL Terrain Running Terrain*Running INTERCEPT=YES<br>DISTRIBUTION=GAMMA LINK=LOG<br>/CRITERIA SCALE=MLE PCONVERGE=1E-006 (ABSOLUTE) SINGULAR=1E-012 ANALYSISTYPE=3 (WALD) CILEVEL=95<br>LIKELIHOOD=FULL<br>/EMMEANS TABLES=Terrain SCALE=ORIGINAL COMPARE=Terrain CONTRAST=PAIRWISE PADJUST=BONFERRONI<br>/EMMEANS TABLES=Running SCALE=ORIGINAL COMPARE=Running CONTRAST=PAIRWISE PADJUST=BONFERRONI<br>/EMMEANS TABLES=Terrain*Running SCALE=ORIGINAL COMPARE=Running*Terrain CONTRAST=PAIRWISE<br><br>PADJUST=BONFERRONI<br>/REPEATED SUBJECT=Subject SORT=YES CORRTYPE=INDEPENDENT ADJUSTCORR=YES COVB=ROBUST<br>/MISSING CLASSMISSING=EXCLUDE<br>/PRINT CPS DESCRIPTIVES MODELINFO FIT SUMMARY SOLUTION.
Resources	Processor Time	00:00:00.06
	Elapsed Time	00:00:00.06

Model Information

Dependent Variable		Swing_phase_Right
Probability Distribution		Gamma
Link Function		Log
Subject Effect	1	Subject
Working Correlation Matrix Structure		Independent

Case Processing Summary

	N	Percent
Included	48	98.0%
Excluded	1	2.0%
Total	49	100.0%

Correlated Data Summary

Number of Levels	Subject Effect	Subject	13
Number of Subjects			13
Number of Measurements per Subject	Minimum		2
	Maximum		4
Correlation Matrix Dimension			4

Categorical Variable Information

			N	Percent
Factor	Terrain	Regular	26	54.2%
		Irregular	22	45.8%
		Total	48	100.0%
	Running	Free	24	50.0%
		Nordic	24	50.0%
		Total	48	100.0%

Continuous Variable Information

		N	Minimum	Maximum	Mean
Dependent Variable	Swing_phase_Right	48	62.37	75.33	67.8586

Continuous Variable Information

		Std. Deviation
Dependent Variable	Swing_phase_Right	2.83687

Goodness of Fit[a]

	Value
Quasi Likelihood under Independence Model Criterion (QIC)[b]	8.110
Corrected Quasi Likelihood under Independence Model Criterion (QICC)[b]	8.081

Dependent Variable: Swing_phase_Right
Model: (Intercept), Terrain, Running, Terrain * Running[a]

a. Information criteria are in smaller-is-better form.

b. Computed using the full log quasi-likelihood function.

Tests of Model Effects

	Type III		
Source	Wald Chi-Square	df	Sig.
(Intercept)	158902.131	1	.000
Terrain	2.204	1	.138
Running	.164	1	.685
Terrain * Running	.385	1	.535

Dependent Variable: Swing_phase_Right
Model: (Intercept), Terrain, Running, Terrain * Running

Parameter Estimates

Parameter	B	Std. Error	95% Wald Confidence Interval Lower	95% Wald Confidence Interval Upper	Hypothesis Test Wald Chi-Square
(Intercept)	4.226	.0122	4.202	4.250	119793.056
[Terrain=1.00]	-.013	.0083	-.029	.004	2.358
[Terrain=2.00]	0[a]	.	.	.	.
[Running=1.00]	-.006	.0082	-.022	.010	.553
[Running=2.00]	0[a]	.	.	.	.
[Terrain=1.00] * [Running=1.00]	.006	.0101	-.013	.026	.385
[Terrain=1.00] * [Running=2.00]	0[a]	.	.	.	.
[Terrain=2.00] * [Running=1.00]	0[a]	.	.	.	.
[Terrain=2.00] * [Running=2.00]	0[a]	.	.	.	.
(Scale)	.002				

Parameter Estimates

Parameter	Hypothesis Test df	Hypothesis Test Sig.
(Intercept)	1	.000
[Terrain=1.00]	1	.125
[Terrain=2.00]	.	.
[Running=1.00]	1	.457
[Running=2.00]	.	.
[Terrain=1.00] * [Running=1.00]	1	.535
[Terrain=1.00] * [Running=2.00]	.	.
[Terrain=2.00] * [Running=1.00]	.	.
[Terrain=2.00] * [Running=2.00]	.	.
(Scale)		

Dependent Variable: Swing_phase_Right
Model: (Intercept), Terrain, Running, Terrain * Running

a. Set to zero because this parameter is redundant.

Estimated Marginal Means 1: Terrain

Estimates

Terrain	Mean	Std. Error	95% Wald Confidence Interval Lower	95% Wald Confidence Interval Upper
Regular	67.5601	.67737	66.2454	68.9009
Irregular	68.2111	.81924	66.6242	69.8359

Pairwise Comparisons

(I) Terrain	(J) Terrain	Mean Difference (I-J)	Std. Error	df	Bonferroni Sig.	95% Wald Confidence ... Lower
Regular	Irregular	-.6510	.44078	1	.140	-1.5149
Irregular	Regular	.6510	.44078	1	.140	-.2129

Pairwise Comparisons

(I) Terrain	(J) Terrain	95% Wald Confidence ... Upper
Regular	Irregular	.2129
Irregular	Regular	1.5149

Pairwise comparisons of estimated marginal means based on the original scale of dependent variable Swing_phase_Right

Overall Test Results

Wald Chi-Square	df	Sig.
2.181	1	.140

The Wald chi-square tests the effect of Terrain. This test is based on the linearly independent pairwise comparisons among the estimated marginal means.

Estimated Marginal Means 2: Running

Estimates

Running	Mean	Std. Error	95% Wald Confidence Interval Lower	95% Wald Confidence Interval Upper
Free	67.7830	.72489	66.3770	69.2188
Nordic	67.9868	.79569	66.4451	69.5644

Pairwise Comparisons

(I) Running	(J) Running	Mean Difference (I-J)	Std. Error	df	Bonferroni Sig.	95% Wald Confidence ... Lower
Free	Nordic	-.2038	.50313	1	.685	-1.1899
Nordic	Free	.2038	.50313	1	.685	-.7823

Pairwise Comparisons

(I) Running	(J) Running	95% Wald Confidence ... Upper
Free	Nordic	.7823
Nordic	Free	1.1899

Pairwise comparisons of estimated marginal means based on the original scale of dependent variable Swing_phase_Right

Overall Test Results

Wald Chi-Square	df	Sig.
.164	1	.685

The Wald chi-square tests the effect of Running. This test is based on the linearly independent pairwise comparisons among the estimated marginal means.

Estimated Marginal Means 3: Terrain* Running

Estimates

Terrain	Running	Mean	Std. Error	95% Wald Confidence Interval Lower	Upper
Regular	Free	67.5642	.63464	66.3317	68.8196
	Nordic	67.5561	.85225	65.9062	69.2473
Irregular	Free	68.0026	.89527	66.2703	69.7801
	Nordic	68.4203	.83534	66.8025	70.0773

Pairwise Comparisons

(I) Running*Terrain	(J) Running*Terrain	Mean Difference (I-J)	Std. Error	df
[Running=1.00]*[Terrain=1.00]	[Running=1.00]*[Terrain=2.00]	-.4384	.55146	1
	[Running=2.00]*[Terrain=1.00]	.0081	.65029	1
	[Running=2.00]*[Terrain=2.00]	-.8562	.72996	1
[Running=1.00]*[Terrain=2.00]	[Running=1.00]*[Terrain=1.00]	.4384	.55146	1
	[Running=2.00]*[Terrain=1.00]	.4465	.60168	1
	[Running=2.00]*[Terrain=2.00]	-.4178	.56093	1
[Running=2.00]*[Terrain=1.00]	[Running=1.00]*[Terrain=1.00]	-.0081	.65029	1
	[Running=1.00]*[Terrain=2.00]	-.4465	.60168	1
	[Running=2.00]*[Terrain=2.00]	-.8643	.56236	1
[Running=2.00]*[Terrain=2.00]	[Running=1.00]*[Terrain=1.00]	.8562	.72996	1
	[Running=1.00]*[Terrain=2.00]	.4178	.56093	1
	[Running=2.00]*[Terrain=1.00]	.8643	.56236	1

Pairwise Comparisons

(I) Running*Terrain	(J) Running*Terrain	Bonferroni Sig.	95% Wald Confidence ... Lower
[Running=1.00]*[Terrain=1.00]	[Running=1.00]*[Terrain=2.00]	1.000	-1.8933
	[Running=2.00]*[Terrain=1.00]	1.000	-1.7075
	[Running=2.00]*[Terrain=2.00]	1.000	-2.7820
[Running=1.00]*[Terrain=2.00]	[Running=1.00]*[Terrain=1.00]	1.000	-1.0165
	[Running=2.00]*[Terrain=1.00]	1.000	-1.1409
	[Running=2.00]*[Terrain=2.00]	1.000	-1.8976
[Running=2.00]*[Terrain=1.00]	[Running=1.00]*[Terrain=1.00]	1.000	-1.7237
	[Running=1.00]*[Terrain=2.00]	1.000	-2.0339
	[Running=2.00]*[Terrain=2.00]	.746	-2.3479
[Running=2.00]*[Terrain=2.00]	[Running=1.00]*[Terrain=1.00]	1.000	-1.0696
	[Running=1.00]*[Terrain=2.00]	1.000	-1.0621
	[Running=2.00]*[Terrain=1.00]	.746	-.6194

Pairwise Comparisons

(I) Running*Terrain	(J) Running*Terrain	95% Wald Confidence ... Upper
[Running=1.00]*[Terrain=1.00]	[Running=1.00]*[Terrain=2.00]	1.0165
	[Running=2.00]*[Terrain=1.00]	1.7237
	[Running=2.00]*[Terrain=2.00]	1.0696
[Running=1.00]*[Terrain=2.00]	[Running=1.00]*[Terrain=1.00]	1.8933
	[Running=2.00]*[Terrain=1.00]	2.0339
	[Running=2.00]*[Terrain=2.00]	1.0621
[Running=2.00]*[Terrain=1.00]	[Running=1.00]*[Terrain=1.00]	1.7075
	[Running=1.00]*[Terrain=2.00]	1.1409
	[Running=2.00]*[Terrain=2.00]	.6194
[Running=2.00]*[Terrain=2.00]	[Running=1.00]*[Terrain=1.00]	2.7820
	[Running=1.00]*[Terrain=2.00]	1.8976
	[Running=2.00]*[Terrain=1.00]	2.3479

Pairwise comparisons of estimated marginal means based on the original scale of dependent variable Swing_phase_Right

Overall Test Results

Wald Chi-Square	df	Sig.
2.589	3	.459

The Wald chi-square tests the effect of Running*Terrain. This test is based on the linearly independent pairwise comparisons among the estimated marginal means.

COORDINATION VARIABLES

```
GENLIN PCI BY Terrain Running (ORDER=ASCENDING)
  /MODEL Terrain Running Terrain*Running INTERCEPT=YES
 DISTRIBUTION=GAMMA LINK=LOG
  /CRITERIA SCALE=MLE PCONVERGE=1E-006(ABSOLUTE) SINGULAR=1E-012 ANALYSISTY
PE=3(WALD) CILEVEL=95
    LIKELIHOOD=FULL
  /EMMEANS TABLES=Terrain SCALE=ORIGINAL COMPARE=Terrain CONTRAST=PAIRWISE
PADJUST=BONFERRONI
  /EMMEANS TABLES=Running SCALE=ORIGINAL COMPARE=Running CONTRAST=PAIRWISE
PADJUST=BONFERRONI
  /EMMEANS TABLES=Terrain*Running SCALE=ORIGINAL COMPARE=Running*Terrain CO
NTRAST=PAIRWISE
    PADJUST=BONFERRONI
  /REPEATED SUBJECT=Subject SORT=YES CORRTYPE=INDEPENDENT ADJUSTCORR=YES CO
VB=ROBUST
  /MISSING CLASSMISSING=EXCLUDE
```

Generalized Linear Models

Notes

Output Created		30-OCT-2023 12:26:24
Comments		
Input	Data	C:\Users\andre\Documents\Andre\Pesquisa\Artigos para Publicar\Nordic Running\Data\Statistics\SPSS\Spatiotemporal.sav
	Active Dataset	DataSet1
	Filter	<none>
	Weight	<none>
	Split File	<none>
	N of Rows in Working Data File	49
Missing Value Handling	Definition of Missing	User-defined missing values for factor, subject and within-subject variables are treated as missing.
	Cases Used	Statistics are based on cases with valid data for all variables in the model.
Weight Handling		not applicable

Notes

Syntax		GENLIN PCI BY Terrain Running (ORDER=ASCENDING)<br>/MODEL Terrain Running Terrain*Running INTERCEPT=YES<br>DISTRIBUTION=GAMMA LINK=LOG<br>/CRITERIA SCALE=MLE PCONVERGE=1E-006 (ABSOLUTE) SINGULAR=1E-012 ANALYSISTYPE=3 (WALD) CILEVEL=95<br>LIKELIHOOD=FULL<br>/EMMEANS TABLES=Terrain SCALE=ORIGINAL COMPARE=Terrain CONTRAST=PAIRWISE PADJUST=BONFERRONI<br>/EMMEANS TABLES=Running SCALE=ORIGINAL COMPARE=Running CONTRAST=PAIRWISE PADJUST=BONFERRONI<br>/EMMEANS TABLES=Terrain*Running SCALE=ORIGINAL COMPARE=Running*Terrain CONTRAST=PAIRWISE<br><br>PADJUST=BONFERRONI<br>/REPEATED SUBJECT=Subject SORT=YES CORRTYPE=INDEPENDENT ADJUSTCORR=YES COVB=ROBUST<br>/MISSING CLASSMISSING=EXCLUDE<br>/PRINT CPS DESCRIPTIVES MODELINFO FIT SUMMARY SOLUTION.
Resources	Processor Time	00:00:00.06
	Elapsed Time	00:00:00.06

Model Information

Dependent Variable		PCI
Probability Distribution		Gamma
Link Function		Log
Subject Effect	1	Subject
Working Correlation Matrix Structure		Independent

Case Processing Summary

	N	Percent
Included	48	98.0%
Excluded	1	2.0%
Total	49	100.0%

Correlated Data Summary

Number of Levels	Subject Effect	Subject	13
Number of Subjects			13
Number of Measurements per Subject	Minimum		2
	Maximum		4
Correlation Matrix Dimension			4

Categorical Variable Information

			N	Percent
Factor	Terrain	Regular	26	54.2%
		Irregular	22	45.8%
		Total	48	100.0%
	Running	Free	24	50.0%
		Nordic	24	50.0%
		Total	48	100.0%

Continuous Variable Information

		N	Minimum	Maximum	Mean	Std. Deviation
Dependent Variable	PCI	48	2.30	7.56	4.3371	1.40525

Goodness of Fit[a]

	Value
Quasi Likelihood under Independence Model Criterion (QIC)[b]	12.426
Corrected Quasi Likelihood under Independence Model Criterion (QICC)[b]	12.221

Dependent Variable: PCI
Model: (Intercept), Terrain, Running, Terrain * Running[a]

a. Information criteria are in smaller-is-better form.

b. Computed using the full log quasi-likelihood function.

Tests of Model Effects

	Type III		
Source	Wald Chi-Square	df	Sig.
(Intercept)	568.285	1	.000
Terrain	3.298	1	.069
Running	2.779	1	.095
Terrain * Running	1.805	1	.179

Dependent Variable: PCI
Model: (Intercept), Terrain, Running, Terrain * Running

Parameter Estimates

Parameter	B	Std. Error	95% Wald Confidence Interval		Hypothesis Test
			Lower	Upper	Wald Chi-Square
(Intercept)	1.490	.1075	1.279	1.701	192.266
[Terrain=1.00]	.073	.0844	-.093	.238	.740
[Terrain=2.00]	0[a]	.	.	.	.
[Running=1.00]	-.203	.1232	-.445	.038	2.724
[Running=2.00]	0[a]	.	.	.	.
[Terrain=1.00] * [Running=1.00]	.128	.0950	-.059	.314	1.805
[Terrain=1.00] * [Running=2.00]	0[a]	.	.	.	.
[Terrain=2.00] * [Running=1.00]	0[a]	.	.	.	.
[Terrain=2.00] * [Running=2.00]	0[a]	.	.	.	.
(Scale)	.097				

Parameter Estimates

Parameter	Hypothesis Test	
	df	Sig.
(Intercept)	1	.000
[Terrain=1.00]	1	.390
[Terrain=2.00]	.	.
[Running=1.00]	1	.099
[Running=2.00]	.	.
[Terrain=1.00] * [Running=1.00]	1	.179
[Terrain=1.00] * [Running=2.00]	.	.
[Terrain=2.00] * [Running=1.00]	.	.
[Terrain=2.00] * [Running=2.00]	.	.
(Scale)		

Dependent Variable: PCI
Model: (Intercept), Terrain, Running, Terrain * Running

a. Set to zero because this parameter is redundant.

Estimated Marginal Means 1: Terrain

Estimates

Terrain	Mean	Std. Error	95% Wald Confidence Interval	
			Lower	Upper
Regular	4.5944	.35625	3.9466	5.3485
Irregular	4.0084	.26217	3.5261	4.5566

Pairwise Comparisons

(I) Terrain	(J) Terrain	Mean Difference (I-J)	Std. Error	df	Bonferroni Sig.	95% Wald Confidence ...
						Lower
Regular	Irregular	.5860	.33186	1	.077	-.0644
Irregular	Regular	-.5860	.33186	1	.077	-1.2365

Pairwise Comparisons

(I) Terrain	(J) Terrain	95% Wald Confidence ...
		Upper
Regular	Irregular	1.2365
Irregular	Regular	.0644

Pairwise comparisons of estimated marginal means based on the original scale of dependent variable PCI

Overall Test Results

Wald Chi-Square	df	Sig.
3.119	1	.077

The Wald chi-square tests the effect of Terrain. This test is based on the linearly independent pairwise comparisons among the estimated marginal means.

Estimated Marginal Means 2: Running

Estimates

Running	Mean	Std. Error	95% Wald Confidence Interval	
			Lower	Upper
Free	4.0023	.21534	3.6017	4.4474
Nordic	4.6014	.41342	3.8584	5.4874

Pairwise Comparisons

(I) Running	(J) Running	Mean Difference (I-J)	Std. Error	df	Bonferroni Sig.	95% Wald Confidence ... Lower
Free	Nordic	-.5991	.37979	1	.115	-1.3435
Nordic	Free	.5991	.37979	1	.115	-.1453

Pairwise Comparisons

(I) Running	(J) Running	95% Wald Confidence ... Upper
Free	Nordic	.1453
Nordic	Free	1.3435

Pairwise comparisons of estimated marginal means based on the original scale of dependent variable PCI

Overall Test Results

Wald Chi-Square	df	Sig.
2.488	1	.115

The Wald chi-square tests the effect of Running. This test is based on the linearly independent pairwise comparisons among the estimated marginal means.

Estimated Marginal Means 3: Terrain* Running

Estimates

Terrain	Running	Mean	Std. Error	95% Wald Confidence Interval Lower	95% Wald Confidence Interval Upper
Regular	Free	4.4238	.32907	3.8237	5.1182
	Nordic	4.7715	.43108	3.9972	5.6959
Irregular	Free	3.6209	.24546	3.1704	4.1354
	Nordic	4.4373	.47683	3.5946	5.4775

Pairwise Comparisons

(I) Running*Terrain	(J) Running*Terrain	Mean Difference (I-J)	Std. Error	df
[Running=1.00]*[Terrain=1.00]	[Running=1.00]*[Terrain=2.00]	.8029	.38117	1
	[Running=2.00]*[Terrain=1.00]	-.3477	.27487	1
	[Running=2.00]*[Terrain=2.00]	-.0134	.50630	1
[Running=1.00]*[Terrain=2.00]	[Running=1.00]*[Terrain=1.00]	-.8029	.38117	1
	[Running=2.00]*[Terrain=1.00]	-1.1506	.48723	1
	[Running=2.00]*[Terrain=2.00]	-.8164	.52159	1
[Running=2.00]*[Terrain=1.00]	[Running=1.00]*[Terrain=1.00]	.3477	.27487	1
	[Running=1.00]*[Terrain=2.00]	1.1506	.48723	1
	[Running=2.00]*[Terrain=2.00]	.3343	.38320	1
[Running=2.00]*[Terrain=2.00]	[Running=1.00]*[Terrain=1.00]	.0134	.50630	1
	[Running=1.00]*[Terrain=2.00]	.8164	.52159	1
	[Running=2.00]*[Terrain=1.00]	-.3343	.38320	1

Pairwise Comparisons

(I) Running*Terrain	(J) Running*Terrain	Bonferroni Sig.	95% Wald Confidence ...
			Lower
[Running=1.00]*[Terrain=1.00]	[Running=1.00]*[Terrain=2.00]	.211	-.2027
	[Running=2.00]*[Terrain=1.00]	1.000	-1.0729
	[Running=2.00]*[Terrain=2.00]	1.000	-1.3492
[Running=1.00]*[Terrain=2.00]	[Running=1.00]*[Terrain=1.00]	.211	-1.8086
	[Running=2.00]*[Terrain=1.00]	.109	-2.4361
	[Running=2.00]*[Terrain=2.00]	.705	-2.1925
[Running=2.00]*[Terrain=1.00]	[Running=1.00]*[Terrain=1.00]	1.000	-.3775
	[Running=1.00]*[Terrain=2.00]	.109	-.1348
	[Running=2.00]*[Terrain=2.00]	1.000	-.6767
[Running=2.00]*[Terrain=2.00]	[Running=1.00]*[Terrain=1.00]	1.000	-1.3223
	[Running=1.00]*[Terrain=2.00]	.705	-.5597
	[Running=2.00]*[Terrain=1.00]	1.000	-1.3452

Pairwise Comparisons

(I) Running*Terrain	(J) Running*Terrain	95% Wald Confidence ... Upper
[Running=1.00]*[Terrain=1.00]	[Running=1.00]*[Terrain=2.00]	1.8086
	[Running=2.00]*[Terrain=1.00]	.3775
	[Running=2.00]*[Terrain=2.00]	1.3223
[Running=1.00]*[Terrain=2.00]	[Running=1.00]*[Terrain=1.00]	.2027
	[Running=2.00]*[Terrain=1.00]	.1348
	[Running=2.00]*[Terrain=2.00]	.5597
[Running=2.00]*[Terrain=1.00]	[Running=1.00]*[Terrain=1.00]	1.0729
	[Running=1.00]*[Terrain=2.00]	2.4361
	[Running=2.00]*[Terrain=2.00]	1.3452
[Running=2.00]*[Terrain=2.00]	[Running=1.00]*[Terrain=1.00]	1.3492
	[Running=1.00]*[Terrain=2.00]	2.1925
	[Running=2.00]*[Terrain=1.00]	.6767

Pairwise comparisons of estimated marginal means based on the original scale of dependent variable PCI

Overall Test Results

Wald Chi-Square	df	Sig.
5.654	3	.130

The Wald chi-square tests the effect of Running*Terrain. This test is based on the linearly independent pairwise comparisons among the estimated marginal means.

```
GENLIN Accuracy BY Terrain Running (ORDER=ASCENDING)
 /MODEL Terrain Running Terrain*Running INTERCEPT=YES
DISTRIBUTION=GAMMA LINK=LOG
```

```
  /CRITERIA SCALE=MLE PCONVERGE=1E-006(ABSOLUTE) SINGULAR=1E-012 ANALYSISTY
PE=3(WALD) CILEVEL=95
    LIKELIHOOD=FULL
  /EMMEANS TABLES=Terrain SCALE=ORIGINAL COMPARE=Terrain CONTRAST=PAIRWISE
PADJUST=BONFERRONI
  /EMMEANS TABLES=Running SCALE=ORIGINAL COMPARE=Running CONTRAST=PAIRWISE
PADJUST=BONFERRONI
  /EMMEANS TABLES=Terrain*Running SCALE=ORIGINAL COMPARE=Running*Terrain CO
NTRAST=PAIRWISE
    PADJUST=BONFERRONI
  /REPEATED SUBJECT=Subject SORT=YES CORRTYPE=INDEPENDENT ADJUSTCORR=YES CO
VB=ROBUST
  /MISSING CLASSMISSING=EXCLUDE
  /PRINT CPS DESCRIPTIVES MODELINFO FIT SUMMARY SOLUTION.
```

Generalized Linear Models

Notes

Output Created		30-OCT-2023 12:30:16
Comments		
Input	Data	C:\Users\andre\Documents\Andre\Pesquisa\Artigos para Publicar\Nordic Running\Data\Statistics\SPSS\Spatiotemporal.sav
	Active Dataset	DataSet1
	Filter	<none>
	Weight	<none>
	Split File	<none>
	N of Rows in Working Data File	49
Missing Value Handling	Definition of Missing	User-defined missing values for factor, subject and within-subject variables are treated as missing.
	Cases Used	Statistics are based on cases with valid data for all variables in the model.
Weight Handling		not applicable

Notes

Syntax		GENLIN Accuracy BY Terrain Running (ORDER=ASCENDING)<br>/MODEL Terrain Running Terrain*Running INTERCEPT=YES<br>DISTRIBUTION=GAMMA LINK=LOG<br>/CRITERIA SCALE=MLE PCONVERGE=1E-006 (ABSOLUTE) SINGULAR=1E-012 ANALYSISTYPE=3 (WALD) CILEVEL=95<br>LIKELIHOOD=FULL<br>/EMMEANS TABLES=Terrain SCALE=ORIGINAL COMPARE=Terrain CONTRAST=PAIRWISE PADJUST=BONFERRONI<br>/EMMEANS TABLES=Running SCALE=ORIGINAL COMPARE=Running CONTRAST=PAIRWISE PADJUST=BONFERRONI<br>/EMMEANS TABLES=Terrain*Running SCALE=ORIGINAL COMPARE=Running*Terrain CONTRAST=PAIRWISE<br><br>PADJUST=BONFERRONI<br>/REPEATED SUBJECT=Subject SORT=YES CORRTYPE=INDEPENDENT ADJUSTCORR=YES COVB=ROBUST<br>/MISSING CLASSMISSING=EXCLUDE<br>/PRINT CPS DESCRIPTIVES MODELINFO FIT SUMMARY SOLUTION.
Resources	Processor Time	00:00:00.06
	Elapsed Time	00:00:00.08

Model Information

Dependent Variable		Accuracy
Probability Distribution		Gamma
Link Function		Log
Subject Effect	1	Subject
Working Correlation Matrix Structure		Independent

Case Processing Summary

	N	Percent
Included	48	98.0%
Excluded	1	2.0%
Total	49	100.0%

Correlated Data Summary

Number of Levels	Subject Effect	Subject	13
Number of Subjects			13
Number of Measurements per Subject	Minimum		2
	Maximum		4
Correlation Matrix Dimension			4

Categorical Variable Information

			N	Percent
Factor	Terrain	Regular	26	54.2%
		Irregular	22	45.8%
		Total	48	100.0%
	Running	Free	24	50.0%
		Nordic	24	50.0%
		Total	48	100.0%

Continuous Variable Information

		N	Minimum	Maximum	Mean	Std. Deviation
Dependent Variable	Accuracy	48	.14	3.49	1.2292	.81080

Goodness of Fit[a]

	Value
Quasi Likelihood under Independence Model Criterion (QIC)[b]	29.888
Corrected Quasi Likelihood under Independence Model Criterion (QICC)[b]	30.388

Dependent Variable: Accuracy
Model: (Intercept), Terrain, Running, Terrain * Running[a]

a. Information criteria are in smaller-is-better form.

b. Computed using the full log quasi-likelihood function.

Tests of Model Effects

Source	Type III		
	Wald Chi-Square	df	Sig.
(Intercept)	2.186	1	.139
Terrain	2.813	1	.093
Running	1.087	1	.297
Terrain * Running	.730	1	.393

Dependent Variable: Accuracy
Model: (Intercept), Terrain, Running, Terrain * Running

Parameter Estimates

Parameter	B	Std. Error	95% Wald Confidence Interval Lower	Upper	Hypothesis Test Wald Chi-Square
(Intercept)	.157	.2059	-.246	.560	.582
[Terrain=1.00]	.219	.2068	-.186	.625	1.125
[Terrain=2.00]	0[a]	.	.	.	.
[Running=1.00]	-.286	.2760	-.827	.255	1.073
[Running=2.00]	0[a]	.	.	.	.
[Terrain=1.00] * [Running=1.00]	.208	.2433	-.269	.685	.730
[Terrain=1.00] * [Running=2.00]	0[a]	.	.	.	.
[Terrain=2.00] * [Running=1.00]	0[a]	.	.	.	.
[Terrain=2.00] * [Running=2.00]	0[a]	.	.	.	.
(Scale)	.439				

Parameter Estimates

Parameter	Hypothesis Test df	Sig.
(Intercept)	1	.446
[Terrain=1.00]	1	.289
[Terrain=2.00]	.	.
[Running=1.00]	1	.300
[Running=2.00]	.	.
[Terrain=1.00] * [Running=1.00]	1	.393
[Terrain=1.00] * [Running=2.00]	.	.
[Terrain=2.00] * [Running=1.00]	.	.
[Terrain=2.00] * [Running=2.00]	.	.
(Scale)		

Dependent Variable: Accuracy
Model: (Intercept), Terrain, Running, Terrain * Running

a. Set to zero because this parameter is redundant.

Estimated Marginal Means 1: Terrain

Estimates

Terrain	Mean	Std. Error	95% Wald Confidence Interval Lower	Upper
Regular	1.4012	.21072	1.0435	1.8816
Irregular	1.0142	.15780	.7476	1.3758

Pairwise Comparisons

(I) Terrain	(J) Terrain	Mean Difference (I-J)	Std. Error	df	Bonferroni Sig.	95% Wald Confidence ... Lower
Regular	Irregular	.3871	.23570	1	.101	-.0749
Irregular	Regular	-.3871	.23570	1	.101	-.8490

Pairwise Comparisons

(I) Terrain	(J) Terrain	95% Wald Confidence ... Upper
Regular	Irregular	.8490
Irregular	Regular	.0749

Pairwise comparisons of estimated marginal means based on the original scale of dependent variable Accuracy

Overall Test Results

Wald Chi-Square	df	Sig.
2.697	1	.101

The Wald chi-square tests the effect of Terrain. This test is based on the linearly independent pairwise comparisons among the estimated marginal means.

Estimated Marginal Means 2: Running

Estimates

Running	Mean	Std. Error	95% Wald Confidence Interval Lower	Upper
Free	1.0885	.15079	.8296	1.4280
Nordic	1.3056	.20345	.9620	1.7720

Pairwise Comparisons

(I) Running	(J) Running	Mean Difference (I-J)	Std. Error	df	Bonferroni Sig.	95% Wald Confidence ... Lower
Free	Nordic	-.2171	.21358	1	.309	-.6357
Nordic	Free	.2171	.21358	1	.309	-.2015

Pairwise Comparisons

(I) Running	(J) Running	95% Wald Confidence ... Upper
Free	Nordic	.2015
Nordic	Free	.6357

Pairwise comparisons of estimated marginal means based on the original scale of dependent variable Accuracy

Overall Test Results

Wald Chi-Square	df	Sig.
1.034	1	.309

The Wald chi-square tests the effect of Running. This test is based on the linearly independent pairwise comparisons among the estimated marginal means.

Estimated Marginal Means 3: Terrain* Running

Estimates

Terrain	Running	Mean	Std. Error	95% Wald Confidence Interval Lower	95% Wald Confidence Interval Upper
Regular	Free	1.3477	.21232	.9897	1.8352
	Nordic	1.4569	.24192	1.0522	2.0173
Irregular	Free	.8791	.18467	.5824	1.3269
	Nordic	1.1700	.24085	.7816	1.7515

Pairwise Comparisons

(I) Running*Terrain	(J) Running*Terrain	Mean Difference (I-J)	Std. Error	df
[Running=1.00]*[Terrain=1.00]	[Running=1.00]*[Terrain=2.00]	.4686	.26442	1
	[Running=2.00]*[Terrain=1.00]	-.1092	.16988	1
	[Running=2.00]*[Terrain=2.00]	.1777	.30436	1
[Running=1.00]*[Terrain=2.00]	[Running=1.00]*[Terrain=1.00]	-.4686	.26442	1
	[Running=2.00]*[Terrain=1.00]	-.5778	.31045	1
	[Running=2.00]*[Terrain=2.00]	-.2909	.28544	1
[Running=2.00]*[Terrain=1.00]	[Running=1.00]*[Terrain=1.00]	.1092	.16988	1
	[Running=1.00]*[Terrain=2.00]	.5778	.31045	1
	[Running=2.00]*[Terrain=2.00]	.2869	.26496	1
[Running=2.00]*[Terrain=2.00]	[Running=1.00]*[Terrain=1.00]	-.1777	.30436	1
	[Running=1.00]*[Terrain=2.00]	.2909	.28544	1
	[Running=2.00]*[Terrain=1.00]	-.2869	.26496	1

Pairwise Comparisons

(I) Running*Terrain	(J) Running*Terrain	Bonferroni Sig.	95% Wald Confidence ... Lower
[Running=1.00]*[Terrain=1.00]	[Running=1.00]*[Terrain=2.00]	.458	-.2290
	[Running=2.00]*[Terrain=1.00]	1.000	-.5574
	[Running=2.00]*[Terrain=2.00]	1.000	-.6253
[Running=1.00]*[Terrain=2.00]	[Running=1.00]*[Terrain=1.00]	.458	-1.1662
	[Running=2.00]*[Terrain=1.00]	.376	-1.3969
	[Running=2.00]*[Terrain=2.00]	1.000	-1.0440
[Running=2.00]*[Terrain=1.00]	[Running=1.00]*[Terrain=1.00]	1.000	-.3390
	[Running=1.00]*[Terrain=2.00]	.376	-.2412
	[Running=2.00]*[Terrain=2.00]	1.000	-.4121
[Running=2.00]*[Terrain=2.00]	[Running=1.00]*[Terrain=1.00]	1.000	-.9807
	[Running=1.00]*[Terrain=2.00]	1.000	-.4622
	[Running=2.00]*[Terrain=1.00]	1.000	-.9859

Pairwise Comparisons

(I) Running*Terrain	(J) Running*Terrain	95% Wald Confidence ... Upper
[Running=1.00]*[Terrain=1.00]	[Running=1.00]*[Terrain=2.00]	1.1662
	[Running=2.00]*[Terrain=1.00]	.3390
	[Running=2.00]*[Terrain=2.00]	.9807
[Running=1.00]*[Terrain=2.00]	[Running=1.00]*[Terrain=1.00]	.2290
	[Running=2.00]*[Terrain=1.00]	.2412
	[Running=2.00]*[Terrain=2.00]	.4622
[Running=2.00]*[Terrain=1.00]	[Running=1.00]*[Terrain=1.00]	.5574
	[Running=1.00]*[Terrain=2.00]	1.3969
	[Running=2.00]*[Terrain=2.00]	.9859
[Running=2.00]*[Terrain=2.00]	[Running=1.00]*[Terrain=1.00]	.6253
	[Running=1.00]*[Terrain=2.00]	1.0440
	[Running=2.00]*[Terrain=1.00]	.4121

Pairwise comparisons of estimated marginal means based on the original scale of dependent variable Accuracy

Overall Test Results

Wald Chi-Square	df	Sig.
3.619	3	.306

The Wald chi-square tests the effect of Running*Terrain. This test is based on the linearly independent pairwise comparisons among the estimated marginal means.

```
GENLIN Variability BY Terrain Running (ORDER=ASCENDING)
 /MODEL Terrain Running Terrain*Running INTERCEPT=YES
DISTRIBUTION=GAMMA LINK=LOG
```

```
  /CRITERIA SCALE=MLE PCONVERGE=1E-006(ABSOLUTE) SINGULAR=1E-012 ANALYSISTY
PE=3(WALD) CILEVEL=95
    LIKELIHOOD=FULL
  /EMMEANS TABLES=Terrain SCALE=ORIGINAL COMPARE=Terrain CONTRAST=PAIRWISE
PADJUST=BONFERRONI
  /EMMEANS TABLES=Running SCALE=ORIGINAL COMPARE=Running CONTRAST=PAIRWISE
PADJUST=BONFERRONI
  /EMMEANS TABLES=Terrain*Running SCALE=ORIGINAL COMPARE=Running*Terrain CO
NTRAST=PAIRWISE
    PADJUST=BONFERRONI
  /REPEATED SUBJECT=Subject SORT=YES CORRTYPE=INDEPENDENT ADJUSTCORR=YES CO
VB=ROBUST
  /MISSING CLASSMISSING=EXCLUDE
  /PRINT CPS DESCRIPTIVES MODELINFO FIT SUMMARY SOLUTION.
```

Generalized Linear Models

Notes

Output Created		30-OCT-2023 12:31:37
Comments		
Input	Data	C:\Users\andre\Documents\Andre\Pesquisa\Artigos para Publicar\Nordic Running\Data\Statistics\SPSS\Spatiotemporal.sav
	Active Dataset	DataSet1
	Filter	<none>
	Weight	<none>
	Split File	<none>
	N of Rows in Working Data File	49
Missing Value Handling	Definition of Missing	User-defined missing values for factor, subject and within-subject variables are treated as missing.
	Cases Used	Statistics are based on cases with valid data for all variables in the model.
Weight Handling		not applicable

Notes

Syntax		GENLIN Variability BY Terrain Running (ORDER=ASCENDING)<br>/MODEL Terrain Running Terrain*Running INTERCEPT=YES<br>DISTRIBUTION=GAMMA LINK=LOG<br>/CRITERIA SCALE=MLE PCONVERGE=1E-006 (ABSOLUTE) SINGULAR=1E-012 ANALYSISTYPE=3 (WALD) CILEVEL=95<br>LIKELIHOOD=FULL<br>/EMMEANS TABLES=Terrain SCALE=ORIGINAL COMPARE=Terrain CONTRAST=PAIRWISE PADJUST=BONFERRONI<br>/EMMEANS TABLES=Running SCALE=ORIGINAL COMPARE=Running CONTRAST=PAIRWISE PADJUST=BONFERRONI<br>/EMMEANS TABLES=Terrain*Running SCALE=ORIGINAL COMPARE=Running*Terrain CONTRAST=PAIRWISE<br><br>PADJUST=BONFERRONI<br>/REPEATED SUBJECT=Subject SORT=YES CORRTYPE=INDEPENDENT ADJUSTCORR=YES COVB=ROBUST<br>/MISSING CLASSMISSING=EXCLUDE<br>/PRINT CPS DESCRIPTIVES MODELINFO FIT SUMMARY SOLUTION.
Resources	Processor Time	00:00:00.06
	Elapsed Time	00:00:00.07

Model Information

Dependent Variable		Variability
Probability Distribution		Gamma
Link Function		Log
Subject Effect	1	Subject
Working Correlation Matrix Structure		Independent

Case Processing Summary

	N	Percent
Included	48	98.0%
Excluded	1	2.0%
Total	49	100.0%

Correlated Data Summary

Number of Levels	Subject Effect	Subject	13
Number of Subjects			13
Number of Measurements per Subject	Minimum		2
	Maximum		4
Correlation Matrix Dimension			4

Categorical Variable Information

			N	Percent
Factor	Terrain	Regular	26	54.2%
		Irregular	22	45.8%
		Total	48	100.0%
	Running	Free	24	50.0%
		Nordic	24	50.0%
		Total	48	100.0%

Continuous Variable Information

		N	Minimum	Maximum	Mean	Std. Deviation
Dependent Variable	Variability	48	1.93	6.04	3.1083	.88261

Goodness of Fit[a]

	Value
Quasi Likelihood under Independence Model Criterion (QIC)[b]	11.951
Corrected Quasi Likelihood under Independence Model Criterion (QICC)[b]	10.960

Dependent Variable: Variability
Model: (Intercept), Terrain, Running, Terrain * Running[a]

a. Information criteria are in smaller-is-better form.

b. Computed using the full log quasi-likelihood function.

Tests of Model Effects

Source	Type III		
	Wald Chi-Square	df	Sig.
(Intercept)	461.127	1	.000
Terrain	.911	1	.340
Running	2.603	1	.107
Terrain * Running	1.927	1	.165

Dependent Variable: Variability
Model: (Intercept), Terrain, Running, Terrain * Running

Parameter Estimates

Parameter	B	Std. Error	95% Wald Confidence Interval Lower	95% Wald Confidence Interval Upper	Hypothesis Test Wald Chi-Square
(Intercept)	1.185	.0977	.993	1.376	146.990
[Terrain=1.00]	.013	.0746	-.133	.159	.031
[Terrain=2.00]	0[a]	.	.	.	.
[Running=1.00]	-.175	.0857	-.343	-.007	4.182
[Running=2.00]	0[a]	.	.	.	.
[Terrain=1.00] * [Running=1.00]	.102	.0731	-.042	.245	1.927
[Terrain=1.00] * [Running=2.00]	0[a]	.	.	.	.
[Terrain=2.00] * [Running=1.00]	0[a]	.	.	.	.
[Terrain=2.00] * [Running=2.00]	0[a]	.	.	.	.
(Scale)	.076				

Parameter Estimates

Parameter	Hypothesis Test df	Hypothesis Test Sig.
(Intercept)	1	.000
[Terrain=1.00]	1	.860
[Terrain=2.00]	.	.
[Running=1.00]	1	.041
[Running=2.00]	.	.
[Terrain=1.00] * [Running=1.00]	1	.165
[Terrain=1.00] * [Running=2.00]	.	.
[Terrain=2.00] * [Running=1.00]	.	.
[Terrain=2.00] * [Running=2.00]	.	.
(Scale)		

Dependent Variable: Variability
Model: (Intercept), Terrain, Running, Terrain * Running

a. Set to zero because this parameter is redundant.

Estimated Marginal Means 1: Terrain

Estimates

Terrain	Mean	Std. Error	95% Wald Confidence Interval Lower	Upper
Regular	3.1924	.20286	2.8186	3.6159
Irregular	2.9949	.18291	2.6570	3.3757

Pairwise Comparisons

(I) Terrain	(J) Terrain	Mean Difference (I-J)	Std. Error	df	Bonferroni Sig.	95% Wald Confidence ... Lower
Regular	Irregular	.1976	.20783	1	.342	-.2098
Irregular	Regular	-.1976	.20783	1	.342	-.6049

Pairwise Comparisons

(I) Terrain	(J) Terrain	95% Wald Confidence ... Upper
Regular	Irregular	.6049
Irregular	Regular	.2098

Pairwise comparisons of estimated marginal means based on the original scale of dependent variable Variability

Overall Test Results

Wald Chi-Square	df	Sig.
.904	1	.342

The Wald chi-square tests the effect of Terrain. This test is based on the linearly independent pairwise comparisons among the estimated marginal means.

Estimated Marginal Means 2: Running

Estimates

Running	Mean	Std. Error	95% Wald Confidence Interval Lower	Upper
Free	2.9055	.11431	2.6899	3.1384
Nordic	3.2906	.27442	2.7944	3.8749

Pairwise Comparisons

(I) Running	(J) Running	Mean Difference (I-J)	Std. Error	df	Bonferroni Sig.	95% Wald Confidence ... Lower
Free	Nordic	-.3851	.25294	1	.128	-.8809
Nordic	Free	.3851	.25294	1	.128	-.1106

Pairwise Comparisons

(I) Running	(J) Running	95% Wald Confidence ... Upper
Free	Nordic	.1106
Nordic	Free	.8809

Pairwise comparisons of estimated marginal means based on the original scale of dependent variable Variability

Overall Test Results

Wald Chi-Square	df	Sig.
2.318	1	.128

The Wald chi-square tests the effect of Running. This test is based on the linearly independent pairwise comparisons among the estimated marginal means.

Estimated Marginal Means 3: Terrain* Running

Estimates

Terrain	Running	Mean	Std. Error	95% Wald Confidence Interval Lower	95% Wald Confidence Interval Upper
Regular	Free	3.0769	.20744	2.6961	3.5116
	Nordic	3.3123	.28002	2.8065	3.9092
Irregular	Free	2.7436	.10928	2.5376	2.9664
	Nordic	3.2691	.31939	2.6994	3.9590

Pairwise Comparisons

(I) Running*Terrain	(J) Running*Terrain	Mean Difference (I-J)	Std. Error	df
[Running=1.00]*[Terrain=1.00]	[Running=1.00]*[Terrain=2.00]	.3333	.23339	1
	[Running=2.00]*[Terrain=1.00]	-.2354	.27569	1
	[Running=2.00]*[Terrain=2.00]	-.1922	.38475	1
[Running=1.00]*[Terrain=2.00]	[Running=1.00]*[Terrain=1.00]	-.3333	.23339	1
	[Running=2.00]*[Terrain=1.00]	-.5687	.26431	1
	[Running=2.00]*[Terrain=2.00]	-.5255	.28278	1
[Running=2.00]*[Terrain=1.00]	[Running=1.00]*[Terrain=1.00]	.2354	.27569	1
	[Running=1.00]*[Terrain=2.00]	.5687	.26431	1
	[Running=2.00]*[Terrain=2.00]	.0432	.24486	1
[Running=2.00]*[Terrain=2.00]	[Running=1.00]*[Terrain=1.00]	.1922	.38475	1
	[Running=1.00]*[Terrain=2.00]	.5255	.28278	1
	[Running=2.00]*[Terrain=1.00]	-.0432	.24486	1

Pairwise Comparisons

(I) Running*Terrain	(J) Running*Terrain	Bonferroni Sig.	95% Wald Confidence ...
			Lower
[Running=1.00]*[Terrain=1.00]	[Running=1.00]*[Terrain=2.00]	.920	-.2824
	[Running=2.00]*[Terrain=1.00]	1.000	-.9627
	[Running=2.00]*[Terrain=2.00]	1.000	-1.2072
[Running=1.00]*[Terrain=2.00]	[Running=1.00]*[Terrain=1.00]	.920	-.9490
	[Running=2.00]*[Terrain=1.00]	.189	-1.2660
	[Running=2.00]*[Terrain=2.00]	.379	-1.2715
[Running=2.00]*[Terrain=1.00]	[Running=1.00]*[Terrain=1.00]	1.000	-.4920
	[Running=1.00]*[Terrain=2.00]	.189	-.1286
	[Running=2.00]*[Terrain=2.00]	1.000	-.6028
[Running=2.00]*[Terrain=2.00]	[Running=1.00]*[Terrain=1.00]	1.000	-.8229
	[Running=1.00]*[Terrain=2.00]	.379	-.2206
	[Running=2.00]*[Terrain=1.00]	1.000	-.6892

Pairwise Comparisons

(I) Running*Terrain	(J) Running*Terrain	95% Wald Confidence ... Upper
[Running=1.00]*[Terrain=1.00]	[Running=1.00]*[Terrain=2.00]	.9490
	[Running=2.00]*[Terrain=1.00]	.4920
	[Running=2.00]*[Terrain=2.00]	.8229
[Running=1.00]*[Terrain=2.00]	[Running=1.00]*[Terrain=1.00]	.2824
	[Running=2.00]*[Terrain=1.00]	.1286
	[Running=2.00]*[Terrain=2.00]	.2206
[Running=2.00]*[Terrain=1.00]	[Running=1.00]*[Terrain=1.00]	.9627
	[Running=1.00]*[Terrain=2.00]	1.2660
	[Running=2.00]*[Terrain=2.00]	.6892
[Running=2.00]*[Terrain=2.00]	[Running=1.00]*[Terrain=1.00]	1.2072
	[Running=1.00]*[Terrain=2.00]	1.2715
	[Running=2.00]*[Terrain=1.00]	.6028

Pairwise comparisons of estimated marginal means based on the original scale of dependent variable Variability

Overall Test Results

Wald Chi-Square	df	Sig.
6.301	3	.098

The Wald chi-square tests the effect of Running*Terrain. This test is based on the linearly independent pairwise comparisons among the estimated marginal means.

PERCEPTUAL RESPONSE VARIABLES

```
GENLIN Borg010 BY Terrain Running (ORDER=ASCENDING)
  /MODEL Terrain Running Terrain*Running INTERCEPT=YES
 DISTRIBUTION=GAMMA LINK=LOG
  /CRITERIA SCALE=MLE PCONVERGE=1E-006(ABSOLUTE) SINGULAR=1E-012 ANALYSISTY
PE=3(WALD) CILEVEL=95
    LIKELIHOOD=FULL
  /EMMEANS TABLES=Terrain SCALE=ORIGINAL COMPARE=Terrain CONTRAST=PAIRWISE
PADJUST=BONFERRONI
  /EMMEANS TABLES=Running SCALE=ORIGINAL COMPARE=Running CONTRAST=PAIRWISE
PADJUST=BONFERRONI
  /EMMEANS TABLES=Terrain*Running SCALE=ORIGINAL COMPARE=Running*Terrain CO
NTRAST=PAIRWISE
    PADJUST=BONFERRONI
  /REPEATED SUBJECT=Subject SORT=YES CORRTYPE=INDEPENDENT ADJUSTCORR=YES CO
VB=ROBUST
  /MISSING CLASSMISSING=EXCLUDE
```

Generalized Linear Models

Notes

Output Created		30-OCT-2023 12:34:48
Comments		
Input	Data	C:\Users\andre\Documents\Andre\Pesquisa\Artigos para Publicar\Nordic Running\Data\Statistics\SPSS\Scales.sav
	Active Dataset	DataSet5
	Filter	<none>
	Weight	<none>
	Split File	<none>
	N of Rows in Working Data File	101
Missing Value Handling	Definition of Missing	User-defined missing values for factor, subject and within-subject variables are treated as missing.
	Cases Used	Statistics are based on cases with valid data for all variables in the model.
Weight Handling		not applicable

Notes

Syntax		GENLIN Borg010 BY Terrain Running (ORDER=ASCENDING)<br>/MODEL Terrain Running Terrain*Running INTERCEPT=YES<br>DISTRIBUTION=GAMMA LINK=LOG<br>/CRITERIA SCALE=MLE PCONVERGE=1E-006 (ABSOLUTE) SINGULAR=1E-012 ANALYSISTYPE=3 (WALD) CILEVEL=95<br>LIKELIHOOD=FULL<br>/EMMEANS TABLES=Terrain SCALE=ORIGINAL COMPARE=Terrain CONTRAST=PAIRWISE PADJUST=BONFERRONI<br>/EMMEANS TABLES=Running SCALE=ORIGINAL COMPARE=Running CONTRAST=PAIRWISE PADJUST=BONFERRONI<br>/EMMEANS TABLES=Terrain*Running SCALE=ORIGINAL COMPARE=Running*Terrain CONTRAST=PAIRWISE<br><br>PADJUST=BONFERRONI<br>/REPEATED SUBJECT=Subject SORT=YES CORRTYPE=INDEPENDENT ADJUSTCORR=YES COVB=ROBUST<br>/MISSING CLASSMISSING=EXCLUDE<br>/PRINT CPS DESCRIPTIVES MODELINFO FIT SUMMARY SOLUTION.
Resources	Processor Time	00:00:00.06
	Elapsed Time	00:00:00.07

Model Information

Dependent Variable		Borg 0-10
Probability Distribution		Gamma
Link Function		Log
Subject Effect	1	Subject
Working Correlation Matrix Structure		Independent

Case Processing Summary

	N	Percent
Included	98	97.0%
Excluded	3	3.0%
Total	101	100.0%

Correlated Data Summary

Number of Levels	Subject Effect	Subject	13
Number of Subjects			13
Number of Measurements per Subject	Minimum		4
	Maximum		10
Correlation Matrix Dimension			10

Categorical Variable Information

			N	Percent
Factor	Terrain	Regular	53	54.1%
		Irregular	45	45.9%
		Total	98	100.0%
	Running	Free	46	46.9%
		Nordic	52	53.1%
		Total	98	100.0%

Continuous Variable Information

		N	Minimum	Maximum	Mean	Std. Deviation
Dependent Variable	Borg 0-10	98	.5	3.0	1.689	.8419

Goodness of Fit[a]

	Value
Quasi Likelihood under Independence Model Criterion (QIC)[b]	40.599
Corrected Quasi Likelihood under Independence Model Criterion (QICC)[b]	35.306

Dependent Variable: Borg 0-10
Model: (Intercept), Terrain, Running, Terrain * Running[a]

a. Information criteria are in smaller-is-better form.

b. Computed using the full log quasi-likelihood function.

Tests of Model Effects

Source	Type III		
	Wald Chi-Square	df	Sig.
(Intercept)	21.349	1	.000
Terrain	3.300	1	.069
Running	5.483	1	.019
Terrain * Running	1.190	1	.275

Dependent Variable: Borg 0-10
Model: (Intercept), Terrain, Running, Terrain * Running

Parameter Estimates

Parameter	B	Std. Error	95% Wald Confidence Interval		Hypothesis Test
			Lower	Upper	Wald Chi-Square
(Intercept)	.673	.1104	.457	.889	37.139
[Terrain=1.00]	-.162	.1134	-.384	.060	2.044
[Terrain=2.00]	0[a]	.	.	.	.
[Running=1.00]	-.085	.0535	-.190	.020	2.533
[Running=2.00]	0[a]	.	.	.	.
[Terrain=1.00] * [Running=1.00]	-.114	.1047	-.319	.091	1.190
[Terrain=1.00] * [Running=2.00]	0[a]	.	.	.	.
[Terrain=2.00] * [Running=1.00]	0[a]	.	.	.	.
[Terrain=2.00] * [Running=2.00]	0[a]	.	.	.	.
(Scale)	.250				

Parameter Estimates

Parameter	Hypothesis Test	
	df	Sig.
(Intercept)	1	.000
[Terrain=1.00]	1	.153
[Terrain=2.00]	.	.
[Running=1.00]	1	.112
[Running=2.00]	.	.
[Terrain=1.00] * [Running=1.00]	1	.275
[Terrain=1.00] * [Running=2.00]	.	.
[Terrain=2.00] * [Running=1.00]	.	.
[Terrain=2.00] * [Running=2.00]	.	.
(Scale)		

Dependent Variable: Borg 0-10
Model: (Intercept), Terrain, Running, Terrain * Running

a. Set to zero because this parameter is redundant.

Estimated Marginal Means 1: Terrain

Estimates

Terrain	Mean	Std. Error	95% Wald Confidence Interval Lower	95% Wald Confidence Interval Upper
Regular	1.509	.2068	1.153	1.973
Irregular	1.878	.2214	1.491	2.367

Pairwise Comparisons

(I) Terrain	(J) Terrain	Mean Difference (I-J)	Std. Error	df	Bonferroni Sig.	95% Wald Confidence ... Lower
Regular	Irregular	-.370	.2011	1	.066	-.764
Irregular	Regular	.370	.2011	1	.066	-.024

Pairwise Comparisons

(I) Terrain	(J) Terrain	95% Wald Confidence ... Upper
Regular	Irregular	.024
Irregular	Regular	.764

Pairwise comparisons of estimated marginal means based on the original scale of dependent variable Borg 0-10

Overall Test Results

Wald Chi-Square	df	Sig.
3.381	1	.066

The Wald chi-square tests the effect of Terrain. This test is based on the linearly independent pairwise comparisons among the estimated marginal means.

Estimated Marginal Means 2: Running

Estimates

Running	Mean	Std. Error	95% Wald Confidence Interval Lower	95% Wald Confidence Interval Upper
Free	1.568	.1848	1.244	1.975
Nordic	1.807	.2089	1.441	2.267

Pairwise Comparisons

(I) Running	(J) Running	Mean Difference (I-J)	Std. Error	df	Bonferroni Sig.	95% Wald Confidence ... Lower
Free	Nordic	-.240[a]	.1050	1	.022	-.446
Nordic	Free	.240[a]	.1050	1	.022	.034

Pairwise Comparisons

(I) Running	(J) Running	95% Wald Confidence ... Upper
Free	Nordic	-.034
Nordic	Free	.446

Pairwise comparisons of estimated marginal means based on the original scale of dependent variable Borg 0-10

a. The mean difference is significant at the .05 level.

Overall Test Results

Wald Chi-Square	df	Sig.
5.209	1	.022

The Wald chi-square tests the effect of Running. This test is based on the linearly independent pairwise comparisons among the estimated marginal means.

Estimated Marginal Means 3: Terrain* Running

Estimates

Terrain	Running	Mean	Std. Error	95% Wald Confidence Interval Lower	95% Wald Confidence Interval Upper
Regular	Free	1.365	.2008	1.024	1.821
	Nordic	1.667	.2413	1.255	2.214
Irregular	Free	1.800	.2349	1.394	2.325
	Nordic	1.960	.2164	1.579	2.434

Pairwise Comparisons

(I) Running*Terrain	(J) Running*Terrain	Mean Difference (I-J)	Std. Error	df
[Running=1.00]*[Terrain=1.00]	[Running=1.00]*[Terrain=2.00]	-.435	.2323	1
	[Running=2.00]*[Terrain=1.00]	-.301	.1562	1
	[Running=2.00]*[Terrain=2.00]	-.595[a]	.2220	1
[Running=1.00]*[Terrain=2.00]	[Running=1.00]*[Terrain=1.00]	.435	.2323	1
	[Running=2.00]*[Terrain=1.00]	.133	.2235	1
	[Running=2.00]*[Terrain=2.00]	-.160	.0950	1
[Running=2.00]*[Terrain=1.00]	[Running=1.00]*[Terrain=1.00]	.301	.1562	1
	[Running=1.00]*[Terrain=2.00]	-.133	.2235	1
	[Running=2.00]*[Terrain=2.00]	-.293	.1969	1
[Running=2.00]*[Terrain=2.00]	[Running=1.00]*[Terrain=1.00]	.595[a]	.2220	1
	[Running=1.00]*[Terrain=2.00]	.160	.0950	1
	[Running=2.00]*[Terrain=1.00]	.293	.1969	1

Pairwise Comparisons

(I) Running*Terrain	(J) Running*Terrain	Bonferroni Sig.	95% Wald Confidence ... Lower
[Running=1.00]*[Terrain=1.00]	[Running=1.00]*[Terrain=2.00]	.368	-1.047
	[Running=2.00]*[Terrain=1.00]	.322	-.713
	[Running=2.00]*[Terrain=2.00]	.044	-1.180
[Running=1.00]*[Terrain=2.00]	[Running=1.00]*[Terrain=1.00]	.368	-.178
	[Running=2.00]*[Terrain=1.00]	1.000	-.456
	[Running=2.00]*[Terrain=2.00]	.552	-.411
[Running=2.00]*[Terrain=1.00]	[Running=1.00]*[Terrain=1.00]	.322	-.111
	[Running=1.00]*[Terrain=2.00]	1.000	-.723
	[Running=2.00]*[Terrain=2.00]	.818	-.813
[Running=2.00]*[Terrain=2.00]	[Running=1.00]*[Terrain=1.00]	.044	.009
	[Running=1.00]*[Terrain=2.00]	.552	-.091
	[Running=2.00]*[Terrain=1.00]	.818	-.226

Pairwise Comparisons

(I) Running*Terrain	(J) Running*Terrain	95% Wald Confidence ... Upper
[Running=1.00]*[Terrain=1.00]	[Running=1.00]*[Terrain=2.00]	.178
	[Running=2.00]*[Terrain=1.00]	.111
	[Running=2.00]*[Terrain=2.00]	-.009
[Running=1.00]*[Terrain=2.00]	[Running=1.00]*[Terrain=1.00]	1.047
	[Running=2.00]*[Terrain=1.00]	.723
	[Running=2.00]*[Terrain=2.00]	.091
[Running=2.00]*[Terrain=1.00]	[Running=1.00]*[Terrain=1.00]	.713
	[Running=1.00]*[Terrain=2.00]	.456
	[Running=2.00]*[Terrain=2.00]	.226
[Running=2.00]*[Terrain=2.00]	[Running=1.00]*[Terrain=1.00]	1.180
	[Running=1.00]*[Terrain=2.00]	.411
	[Running=2.00]*[Terrain=1.00]	.813

Pairwise comparisons of estimated marginal means based on the original scale of dependent variable Borg 0-10

a. The mean difference is significant at the .05 level.

Overall Test Results

Wald Chi-Square	df	Sig.
9.339	3	.025

The Wald chi-square tests the effect of Running*Terrain. This test is based on the linearly independent pairwise comparisons among the estimated marginal means.

```
GENLIN Prazer5a5 BY Terrain Running (ORDER=ASCENDING)
 /MODEL Terrain Running Terrain*Running INTERCEPT=YES
```

```
 DISTRIBUTION=IGAUSS LINK=LOG
  /CRITERIA SCALE=MLE PCONVERGE=1E-006(ABSOLUTE) SINGULAR=1E-012 ANALYSISTY
PE=3(WALD) CILEVEL=95
    LIKELIHOOD=FULL
  /EMMEANS TABLES=Terrain SCALE=ORIGINAL COMPARE=Terrain CONTRAST=PAIRWISE
PADJUST=BONFERRONI
  /EMMEANS TABLES=Running SCALE=ORIGINAL COMPARE=Running CONTRAST=PAIRWISE
PADJUST=BONFERRONI
  /EMMEANS TABLES=Terrain*Running SCALE=ORIGINAL COMPARE=Running*Terrain CO
NTRAST=PAIRWISE
    PADJUST=BONFERRONI
  /REPEATED SUBJECT=Subject SORT=YES CORRTYPE=INDEPENDENT ADJUSTCORR=YES CO
VB=ROBUST
  /MISSING CLASSMISSING=EXCLUDE
  /PRINT CPS DESCRIPTIVES MODELINFO FIT SUMMARY SOLUTION.
```

Generalized Linear Models

Notes

Output Created		30-OCT-2023 12:37:06
Comments		
Input	Data	C:\Users\andre\Documents\Andre\Pesquisa\Artigos para Publicar\Nordic Running\Data\Statistics\SPSS\Scales.sav
	Active Dataset	DataSet5
	Filter	<none>
	Weight	<none>
	Split File	<none>
	N of Rows in Working Data File	101
Missing Value Handling	Definition of Missing	User-defined missing values for factor, subject and within-subject variables are treated as missing.
	Cases Used	Statistics are based on cases with valid data for all variables in the model.
Weight Handling		not applicable

Notes

Syntax		GENLIN Prazer5a5 BY Terrain Running (ORDER=ASCENDING)<br>/MODEL Terrain Running Terrain*Running INTERCEPT=YES<br>DISTRIBUTION=IGAUSS LINK=LOG<br>/CRITERIA SCALE=MLE PCONVERGE=1E-006 (ABSOLUTE) SINGULAR=1E-012 ANALYSISTYPE=3 (WALD) CILEVEL=95<br>LIKELIHOOD=FULL<br>/EMMEANS TABLES=Terrain SCALE=ORIGINAL COMPARE=Terrain CONTRAST=PAIRWISE PADJUST=BONFERRONI<br>/EMMEANS TABLES=Running SCALE=ORIGINAL COMPARE=Running CONTRAST=PAIRWISE PADJUST=BONFERRONI<br>/EMMEANS TABLES=Terrain*Running SCALE=ORIGINAL COMPARE=Running*Terrain CONTRAST=PAIRWISE<br><br>PADJUST=BONFERRONI<br>/REPEATED SUBJECT=Subject SORT=YES CORRTYPE=INDEPENDENT ADJUSTCORR=YES COVB=ROBUST<br>/MISSING CLASSMISSING=EXCLUDE<br>/PRINT CPS DESCRIPTIVES MODELINFO FIT SUMMARY SOLUTION.
Resources	Processor Time	00:00:00.06
	Elapsed Time	00:00:00.06

Model Information

Dependent Variable		Prazer (-5 a +5)
Probability Distribution		Inverse Gaussian
Link Function		Log
Subject Effect	1	Subject
Working Correlation Matrix Structure		Independent

Case Processing Summary

	N	Percent
Included	98	97.0%
Excluded	3	3.0%
Total	101	100.0%

Correlated Data Summary

Number of Levels	Subject Effect	Subject	13
Number of Subjects			13
Number of Measurements per Subject	Minimum		4
	Maximum		10
Correlation Matrix Dimension			10

Categorical Variable Information

			N	Percent
Factor	Terrain	Regular	53	54.1%
		Irregular	45	45.9%
		Total	98	100.0%
	Running	Free	46	46.9%
		Nordic	52	53.1%
		Total	98	100.0%

Continuous Variable Information

		N	Minimum	Maximum	Mean
Dependent Variable	Prazer (-5 a +5)	98	1	5	3.48

Continuous Variable Information

		Std. Deviation
Dependent Variable	Prazer (-5 a +5)	1.077

Goodness of Fit[a]

	Value
Quasi Likelihood under Independence Model Criterion (QIC)[b]	9.836
Corrected Quasi Likelihood under Independence Model Criterion (QICC)[b]	11.873

Dependent Variable: Prazer (-5 a +5)
Model: (Intercept), Terrain, Running, Terrain * Running[a]

a. Information criteria are in smaller-is-better form.

b. Computed using the full log quasi-likelihood function.

Tests of Model Effects

Source	Type III		
	Wald Chi-Square	df	Sig.
(Intercept)	848.901	1	.000
Terrain	8.415	1	.004
Running	.903	1	.342
Terrain * Running	.017	1	.897

Dependent Variable: Prazer (-5 a +5)
Model: (Intercept), Terrain, Running, Terrain * Running

Parameter Estimates

Parameter	B	Std. Error	95% Wald Confidence Interval Lower	95% Wald Confidence Interval Upper	Hypothesis Test Wald Chi-Square
(Intercept)	1.085	.0223	1.042	1.129	2373.650
[Terrain=1.00]	.254	.0897	.078	.430	7.995
[Terrain=2.00]	0[a]	.	.	.	.
[Running=1.00]	.030	.0192	-.008	.068	2.425
[Running=2.00]	0[a]	.	.	.	.
[Terrain=1.00] * [Running=1.00]	.008	.0599	-.110	.125	.017
[Terrain=1.00] * [Running=2.00]	0[a]	.	.	.	.
[Terrain=2.00] * [Running=1.00]	0[a]	.	.	.	.
[Terrain=2.00] * [Running=2.00]	0[a]	.	.	.	.
(Scale)	.020				

Parameter Estimates

Parameter	Hypothesis Test df	Hypothesis Test Sig.
(Intercept)	1	.000
[Terrain=1.00]	1	.005
[Terrain=2.00]	.	.
[Running=1.00]	1	.119
[Running=2.00]	.	.
[Terrain=1.00] * [Running=1.00]	1	.897
[Terrain=1.00] * [Running=2.00]	.	.
[Terrain=2.00] * [Running=1.00]	.	.
[Terrain=2.00] * [Running=2.00]	.	.
(Scale)		

Dependent Variable: Prazer (-5 a +5)
Model: (Intercept), Terrain, Running, Terrain * Running

a. Set to zero because this parameter is redundant.

Estimated Marginal Means 1: Terrain

Estimates

Terrain	Mean	Std. Error	95% Wald Confidence Interval Lower	95% Wald Confidence Interval Upper
Regular	3.89	.325	3.30	4.58
Irregular	3.00	.069	2.87	3.14

Pairwise Comparisons

(I) Terrain	(J) Terrain	Mean Difference (I-J)	Std. Error	df	Bonferroni Sig.	95% Wald Confidence ... Lower
Regular	Irregular	.88[a]	.339	1	.009	.22
Irregular	Regular	-.88[a]	.339	1	.009	-1.55

Pairwise Comparisons

(I) Terrain	(J) Terrain	95% Wald Confidence ... Upper
Regular	Irregular	1.55
Irregular	Regular	-.22

Pairwise comparisons of estimated marginal means based on the original scale of dependent variable Prazer (-5 a +5)

a. The mean difference is significant at the .05 level.

Overall Test Results

Wald Chi-Square	df	Sig.
6.800	1	.009

The Wald chi-square tests the effect of Terrain. This test is based on the linearly independent pairwise comparisons among the estimated marginal means.

Estimated Marginal Means 2: Running

Estimates

Running	Mean	Std. Error	95% Wald Confidence Interval Lower	95% Wald Confidence Interval Upper
Free	3.48	.149	3.20	3.78
Nordic	3.36	.163	3.06	3.70

Pairwise Comparisons

(I) Running	(J) Running	Mean Difference (I-J)	Std. Error	df	Bonferroni Sig.	95% Wald Confidence ... Lower
Free	Nordic	.12	.121	1	.339	-.12
Nordic	Free	-.12	.121	1	.339	-.35

Pairwise Comparisons

(I) Running	(J) Running	95% Wald Confidence ... Upper
Free	Nordic	.35
Nordic	Free	.12

Pairwise comparisons of estimated marginal means based on the original scale of dependent variable Prazer (-5 a +5)

Overall Test Results

Wald Chi-Square	df	Sig.
.914	1	.339

The Wald chi-square tests the effect of Running. This test is based on the linearly independent pairwise comparisons among the estimated marginal means.

Estimated Marginal Means 3: Terrain* Running

Estimates

Terrain	Running	Mean	Std. Error	95% Wald Confidence Interval	
				Lower	Upper
Regular	Free	3.96	.347	3.34	4.70
	Nordic	3.81	.346	3.19	4.56
Irregular	Free	3.05	.084	2.89	3.22
	Nordic	2.96	.066	2.83	3.09

Pairwise Comparisons

(I) Running*Terrain	(J) Running*Terrain	Mean Difference (I-J)	Std. Error	df
[Running=1.00]*[Terrain=1.00]	[Running=1.00]*[Terrain=2.00]	.91	.375	1
	[Running=2.00]*[Terrain=1.00]	.15	.244	1
	[Running=2.00]*[Terrain=2.00]	1.00[a]	.353	1
[Running=1.00]*[Terrain=2.00]	[Running=1.00]*[Terrain=1.00]	-.91	.375	1
	[Running=2.00]*[Terrain=1.00]	-.76	.375	1
	[Running=2.00]*[Terrain=2.00]	.09	.058	1
[Running=2.00]*[Terrain=1.00]	[Running=1.00]*[Terrain=1.00]	-.15	.244	1
	[Running=1.00]*[Terrain=2.00]	.76	.375	1
	[Running=2.00]*[Terrain=2.00]	.85	.341	1
[Running=2.00]*[Terrain=2.00]	[Running=1.00]*[Terrain=1.00]	-1.00[a]	.353	1
	[Running=1.00]*[Terrain=2.00]	-.09	.058	1
	[Running=2.00]*[Terrain=1.00]	-.85	.341	1

Pairwise Comparisons

(I) Running*Terrain	(J) Running*Terrain	Bonferroni Sig.	95% Wald Confidence ...
			Lower
[Running=1.00]*[Terrain=1.00]	[Running=1.00]*[Terrain=2.00]	.090	-.08
	[Running=2.00]*[Terrain=1.00]	1.000	-.50
	[Running=2.00]*[Terrain=2.00]	.027	.07
[Running=1.00]*[Terrain=2.00]	[Running=1.00]*[Terrain=1.00]	.090	-1.90
	[Running=2.00]*[Terrain=1.00]	.248	-1.75
	[Running=2.00]*[Terrain=2.00]	.741	-.06
[Running=2.00]*[Terrain=1.00]	[Running=1.00]*[Terrain=1.00]	1.000	-.79
	[Running=1.00]*[Terrain=2.00]	.248	-.22
	[Running=2.00]*[Terrain=2.00]	.074	-.05
[Running=2.00]*[Terrain=2.00]	[Running=1.00]*[Terrain=1.00]	.027	-1.93
	[Running=1.00]*[Terrain=2.00]	.741	-.24
	[Running=2.00]*[Terrain=1.00]	.074	-1.76

Pairwise Comparisons

(I) Running*Terrain	(J) Running*Terrain	95% Wald Confidence ... Upper
[Running=1.00]*[Terrain=1.00]	[Running=1.00]*[Terrain=2.00]	1.90
	[Running=2.00]*[Terrain=1.00]	.79
	[Running=2.00]*[Terrain=2.00]	1.93
[Running=1.00]*[Terrain=2.00]	[Running=1.00]*[Terrain=1.00]	.08
	[Running=2.00]*[Terrain=1.00]	.22
	[Running=2.00]*[Terrain=2.00]	.24
[Running=2.00]*[Terrain=1.00]	[Running=1.00]*[Terrain=1.00]	.50
	[Running=1.00]*[Terrain=2.00]	1.75
	[Running=2.00]*[Terrain=2.00]	1.76
[Running=2.00]*[Terrain=2.00]	[Running=1.00]*[Terrain=1.00]	-.07
	[Running=1.00]*[Terrain=2.00]	.06
	[Running=2.00]*[Terrain=1.00]	.05

Pairwise comparisons of estimated marginal means based on the original scale of dependent variable Prazer (-5 a +5)

a. The mean difference is significant at the .05 level.

Overall Test Results

Wald Chi-Square	df	Sig.
18.229	3	.000

The Wald chi-square tests the effect of Running*Terrain. This test is based on the linearly independent pairwise comparisons among the estimated marginal means.

ANGULAR VARIABLES

JOINT'S ROM - CONTACT PHASE

```
 GET
  FILE='C:\Users\andre\Documents\Andre\Pesquisa\Artigos para Publicar\Nordi
c Running\Data\Statistics\SPSS\Angular_Parameters_Range_Swing.sav'.
DATASET NAME DataSet10 WINDOW=FRONT.
GET
  FILE='C:\Users\andre\Documents\Andre\Pesquisa\Artigos para Publicar\Nordi
c Running\Data\Statistics\SPSS\Angular_Parameters_Range_Contact.sav'.
DATASET NAME DataSet11 WINDOW=FRONT.
GENLIN HipFlexionRT BY Terrain Running (ORDER=ASCENDING)
  /MODEL Terrain Running Terrain*Running INTERCEPT=YES
 DISTRIBUTION=GAMMA LINK=LOG
  /CRITERIA SCALE=MLE PCONVERGE=1E-006(ABSOLUTE) SINGULAR=1E-012 ANALYSISTY
PE=3(WALD) CILEVEL=95
    LIKELIHOOD=FULL
  /EMMEANS TABLES=Terrain SCALE=ORIGINAL COMPARE=Terrain CONTRAST=PAIRWISE
PADJUST=BONFERRONI
  /EMMEANS TABLES=Running SCALE=ORIGINAL COMPARE=Running CONTRAST=PAIRWISE
PADJUST=BONFERRONI
  /EMMEANS TABLES=Terrain*Running SCALE=ORIGINAL COMPARE=Running*Terrain CO
NTRAST=PAIRWISE
    PADJUST=BONFERRONI
  /REPEATED SUBJECT=Subject SORT=YES CORRTYPE=INDEPENDENT ADJUSTCORR=YES CO
VB=ROBUST
  /MISSING CLASSMISSING=EXCLUDE
  /PRINT CPS DESCRIPTIVES MODELINFO FIT SUMMARY SOLUTION.
```

Generalized Linear Models

Notes

Output Created		31-OCT-2023 12:50:46
Comments		
Input	Data	C: \Users\andre\Documents\Andre\Pesquisa\Artigos para Publicar\Nordic Running\Data\Statistics\SPSS\Angular_Parameters_Range_Contact.sav
	Active Dataset	DataSet11
	Filter	<none>
	Weight	<none>
	Split File	<none>
	N of Rows in Working Data File	48
Missing Value Handling	Definition of Missing	User-defined missing values for factor, subject and within-subject variables are treated as missing.
	Cases Used	Statistics are based on cases with valid data for all variables in the model.
Weight Handling		not applicable

Notes

Syntax		GENLIN HipFlexionRT BY Terrain Running (ORDER=ASCENDING)<br>/MODEL Terrain Running Terrain*Running INTERCEPT=YES<br>DISTRIBUTION=GAMMA LINK=LOG<br>/CRITERIA SCALE=MLE PCONVERGE=1E-006 (ABSOLUTE) SINGULAR=1E-012 ANALYSISTYPE=3 (WALD) CILEVEL=95<br>LIKELIHOOD=FULL<br>/EMMEANS TABLES=Terrain SCALE=ORIGINAL COMPARE=Terrain CONTRAST=PAIRWISE PADJUST=BONFERRONI<br>/EMMEANS TABLES=Running SCALE=ORIGINAL COMPARE=Running CONTRAST=PAIRWISE PADJUST=BONFERRONI<br>/EMMEANS TABLES=Terrain*Running SCALE=ORIGINAL COMPARE=Running*Terrain CONTRAST=PAIRWISE<br><br>PADJUST=BONFERRONI<br>/REPEATED SUBJECT=Subject SORT=YES CORRTYPE=INDEPENDENT ADJUSTCORR=YES COVB=ROBUST<br>/MISSING CLASSMISSING=EXCLUDE<br>/PRINT CPS DESCRIPTIVES MODELINFO FIT SUMMARY SOLUTION.
Resources	Processor Time	00:00:00.06
	Elapsed Time	00:00:00.06

```
[DataSet11] C:\Users\andre\Documents\Andre\Pesquisa\Artigos para Publicar\Nordic Running\Data\Statistics\SPSS\Angular_Parameters_Range_Contact.sav
```

Model Information

Dependent Variable	HipFlexionRT
Probability Distribution	Gamma
Link Function	Log
Subject Effect 1	Subject
Working Correlation Matrix Structure	Independent

Case Processing Summary

	N	Percent
Included	48	100.0%
Excluded	0	0.0%
Total	48	100.0%

Correlated Data Summary

Number of Levels	Subject Effect	Subject	13
Number of Subjects			13
Number of Measurements per Subject	Minimum		2
	Maximum		4
Correlation Matrix Dimension			4

Categorical Variable Information

			N	Percent
Factor	Terrain	Regular	26	54.2%
		Irregular	22	45.8%
		Total	48	100.0%
	Running	Free	24	50.0%
		Nordic	24	50.0%
		Total	48	100.0%

Continuous Variable Information

		N	Minimum	Maximum	Mean	Std. Deviation
Dependent Variable	HipFlexionRT	48	36.4	59.6	48.147	6.4037

Goodness of Fit[a]

	Value
Quasi Likelihood under Independence Model Criterion (QIC)[b]	8.482
Corrected Quasi Likelihood under Independence Model Criterion (QICC)[b]	8.767

Dependent Variable: HipFlexionRT
Model: (Intercept), Terrain, Running, Terrain * Running

a. Information criteria are in smaller-is-better form.

b. Computed using the full log quasi-likelihood function.

Tests of Model Effects

	Type III		
Source	Wald Chi-Square	df	Sig.
(Intercept)	15690.853	1	.000
Terrain	10.215	1	.001
Running	.000	1	.985
Terrain * Running	.003	1	.957

Dependent Variable: HipFlexionRT
Model: (Intercept), Terrain, Running, Terrain * Running

Parameter Estimates

Parameter	B	Std. Error	95% Wald Confidence Interval		Hypothesis Test
			Lower	Upper	Wald Chi-Square
(Intercept)	3.920	.0343	3.853	3.988	13084.590
[Terrain=1.000]	-.087	.0271	-.140	-.034	10.307
[Terrain=2.000]	0[a]	.	.	.	.
[Running=1.00]	.001	.0200	-.038	.040	.003
[Running=2.00]	0[a]	.	.	.	.
[Terrain=1.000] * [Running=1.00]	-.002	.0313	-.063	.060	.003
[Terrain=1.000] * [Running=2.00]	0[a]	.	.	.	.
[Terrain=2.000] * [Running=1.00]	0[a]	.	.	.	.
[Terrain=2.000] * [Running=2.00]	0[a]	.	.	.	.
(Scale)	.017				

Parameter Estimates

Parameter	Hypothesis Test	
	df	Sig.
(Intercept)	1	.000
[Terrain=1.000]	1	.001
[Terrain=2.000]	.	.
[Running=1.00]	1	.954
[Running=2.00]	.	.
[Terrain=1.000] * [Running=1.00]	1	.957
[Terrain=1.000] * [Running=2.00]	.	.
[Terrain=2.000] * [Running=1.00]	.	.
[Terrain=2.000] * [Running=2.00]	.	.
(Scale)		

Dependent Variable: HipFlexionRT
Model: (Intercept), Terrain, Running, Terrain * Running

a. Set to zero because this parameter is redundant.

Estimated Marginal Means 1: Terrain

Estimates

Terrain	Mean	Std. Error	95% Wald Confidence Interval Lower	95% Wald Confidence Interval Upper
Regular	46.201	1.5351	43.288	49.310
Irregular	50.447	1.7403	47.149	53.976

Pairwise Comparisons

(I) Terrain	(J) Terrain	Mean Difference (I-J)	Std. Error	df	Bonferroni Sig.	95% Wald Confidence ... Lower
Regular	Irregular	-4.245[a]	1.3422	1	.002	-6.876
Irregular	Regular	4.245[a]	1.3422	1	.002	1.615

Pairwise Comparisons

(I) Terrain	(J) Terrain	95% Wald Confidence ... Upper
Regular	Irregular	-1.615
Irregular	Regular	6.876

Pairwise comparisons of estimated marginal means based on the original scale of dependent variable HipFlexionRT

a. The mean difference is significant at the .05 level.

Overall Test Results

Wald Chi-Square	df	Sig.
10.004	1	.002

The Wald chi-square tests the effect of Terrain. This test is based on the linearly independent pairwise comparisons among the estimated marginal means.

Estimated Marginal Means 2: Running

Estimates

Running	Mean	Std. Error	95% Wald Confidence Interval Lower	95% Wald Confidence Interval Upper
Free	48.285	1.5503	45.340	51.422
Nordic	48.270	1.5499	45.326	51.405

Pairwise Comparisons

(I) Running	(J) Running	Mean Difference (I-J)	Std. Error	df	Bonferroni Sig.	95% Wald Confidence ... Lower
Free	Nordic	.016	.8250	1	.985	-1.601
Nordic	Free	-.016	.8250	1	.985	-1.632

Pairwise Comparisons

(I) Running	(J) Running	95% Wald Confidence ... Upper
Free	Nordic	1.632
Nordic	Free	1.601

Pairwise comparisons of estimated marginal means based on the original scale of dependent variable HipFlexionRT

Overall Test Results

Wald Chi-Square	df	Sig.
.000	1	.985

The Wald chi-square tests the effect of Running. This test is based on the linearly independent pairwise comparisons among the estimated marginal means.

Estimated Marginal Means 3: Terrain* Running

Estimates

Terrain	Running	Mean	Std. Error	95% Wald Confidence Interval Lower	95% Wald Confidence Interval Upper
Regular	Free	46.190	1.6589	43.050	49.558
	Nordic	46.213	1.6372	43.113	49.536
Irregular	Free	50.476	1.8922	46.900	54.324
	Nordic	50.418	1.7279	47.142	53.921

Pairwise Comparisons

(I) Running*Terrain	(J) Running*Terrain	Mean Difference (I-J)	Std. Error	df
[Running=1.00]*[Terrain=1.000]	[Running=1.00]*[Terrain=2.000]	-4.286	1.7325	1
	[Running=2.00]*[Terrain=1.000]	-.024	1.1993	1
	[Running=2.00]*[Terrain=2.000]	-4.228[a]	1.5267	1
[Running=1.00]*[Terrain=2.000]	[Running=1.00]*[Terrain=1.000]	4.286	1.7325	1
	[Running=2.00]*[Terrain=1.000]	4.263	1.6157	1
	[Running=2.00]*[Terrain=2.000]	.058	1.0084	1
[Running=2.00]*[Terrain=1.000]	[Running=1.00]*[Terrain=1.000]	.024	1.1993	1
	[Running=1.00]*[Terrain=2.000]	-4.263	1.6157	1
	[Running=2.00]*[Terrain=2.000]	-4.204[a]	1.3111	1
[Running=2.00]*[Terrain=2.000]	[Running=1.00]*[Terrain=1.000]	4.228[a]	1.5267	1
	[Running=1.00]*[Terrain=2.000]	-.058	1.0084	1
	[Running=2.00]*[Terrain=1.000]	4.204[a]	1.3111	1

Pairwise Comparisons

(I) Running*Terrain	(J) Running*Terrain	Bonferroni Sig.	95% Wald Confidence ... Lower
[Running=1.00]*[Terrain=1.000]	[Running=1.00]*[Terrain=2.000]	.080	-8.857
	[Running=2.00]*[Terrain=1.000]	1.000	-3.188
	[Running=2.00]*[Terrain=2.000]	.034	-8.256
[Running=1.00]*[Terrain=2.000]	[Running=1.00]*[Terrain=1.000]	.080	-.284
	[Running=2.00]*[Terrain=1.000]	.050	.000
	[Running=2.00]*[Terrain=2.000]	1.000	-2.602
[Running=2.00]*[Terrain=1.000]	[Running=1.00]*[Terrain=1.000]	1.000	-3.140
	[Running=1.00]*[Terrain=2.000]	.050	-8.525
	[Running=2.00]*[Terrain=2.000]	.008	-7.664
[Running=2.00]*[Terrain=2.000]	[Running=1.00]*[Terrain=1.000]	.034	.200
	[Running=1.00]*[Terrain=2.000]	1.000	-2.719
	[Running=2.00]*[Terrain=1.000]	.008	.745

Pairwise Comparisons

(I) Running*Terrain	(J) Running*Terrain	95% Wald Confidence ... Upper
[Running=1.00]*[Terrain=1.000]	[Running=1.00]*[Terrain=2.000]	.284
	[Running=2.00]*[Terrain=1.000]	3.140
	[Running=2.00]*[Terrain=2.000]	-.200
[Running=1.00]*[Terrain=2.000]	[Running=1.00]*[Terrain=1.000]	8.857
	[Running=2.00]*[Terrain=1.000]	8.525
	[Running=2.00]*[Terrain=2.000]	2.719
[Running=2.00]*[Terrain=1.000]	[Running=1.00]*[Terrain=1.000]	3.188
	[Running=1.00]*[Terrain=2.000]	.000
	[Running=2.00]*[Terrain=2.000]	-.745
[Running=2.00]*[Terrain=2.000]	[Running=1.00]*[Terrain=1.000]	8.256
	[Running=1.00]*[Terrain=2.000]	2.602
	[Running=2.00]*[Terrain=1.000]	7.664

Pairwise comparisons of estimated marginal means based on the original scale of dependent variable HipFlexionRT

a. The mean difference is significant at the .05 level.

Overall Test Results

Wald Chi-Square	df	Sig.
11.177	3	.011

The Wald chi-square tests the effect of Running*Terrain. This test is based on the linearly independent pairwise comparisons among the estimated marginal means.

```
GENLIN KneeFlexionRT BY Terrain Running (ORDER=ASCENDING)
```

```
  /MODEL Terrain Running Terrain*Running INTERCEPT=YES
  DISTRIBUTION=GAMMA LINK=LOG
  /CRITERIA SCALE=MLE PCONVERGE=1E-006(ABSOLUTE) SINGULAR=1E-012 ANALYSISTY
PE=3(WALD) CILEVEL=95
    LIKELIHOOD=FULL
  /EMMEANS TABLES=Terrain SCALE=ORIGINAL COMPARE=Terrain CONTRAST=PAIRWISE
PADJUST=BONFERRONI
  /EMMEANS TABLES=Running SCALE=ORIGINAL COMPARE=Running CONTRAST=PAIRWISE
PADJUST=BONFERRONI
  /EMMEANS TABLES=Terrain*Running SCALE=ORIGINAL COMPARE=Running*Terrain CO
NTRAST=PAIRWISE
    PADJUST=BONFERRONI
  /REPEATED SUBJECT=Subject SORT=YES CORRTYPE=INDEPENDENT ADJUSTCORR=YES CO
VB=ROBUST
  /MISSING CLASSMISSING=EXCLUDE
  /PRINT CPS DESCRIPTIVES MODELINFO FIT SUMMARY SOLUTION.
```

Generalized Linear Models

Notes

Output Created		31-OCT-2023 12:50:46
Comments		
Input	Data	C:\Users\andre\Documents\Andre\Pesquisa\Artigos para Publicar\Nordic Running\Data\Statistics\SPSS\Angular_Parameters_Range_Contact.sav
	Active Dataset	DataSet11
	Filter	<none>
	Weight	<none>
	Split File	<none>
	N of Rows in Working Data File	48
Missing Value Handling	Definition of Missing	User-defined missing values for factor, subject and within-subject variables are treated as missing.
	Cases Used	Statistics are based on cases with valid data for all variables in the model.
Weight Handling		not applicable

Notes

Syntax		GENLIN KneeFlexionRT BY Terrain Running (ORDER=ASCENDING)<br>/MODEL Terrain Running Terrain*Running INTERCEPT=YES<br><br>DISTRIBUTION=GAMMA LINK=LOG<br>/CRITERIA SCALE=MLE PCONVERGE=1E-006 (ABSOLUTE) SINGULAR=1E-012 ANALYSISTYPE=3 (WALD) CILEVEL=95<br>LIKELIHOOD=FULL<br>/EMMEANS TABLES=Terrain SCALE=ORIGINAL COMPARE=Terrain CONTRAST=PAIRWISE PADJUST=BONFERRONI<br>/EMMEANS TABLES=Running SCALE=ORIGINAL COMPARE=Running CONTRAST=PAIRWISE PADJUST=BONFERRONI<br>/EMMEANS TABLES=Terrain*Running SCALE=ORIGINAL COMPARE=Running*Terrain CONTRAST=PAIRWISE<br><br>PADJUST=BONFERRONI<br>/REPEATED SUBJECT=Subject SORT=YES CORRTYPE=INDEPENDENT ADJUSTCORR=YES COVB=ROBUST<br>/MISSING CLASSMISSING=EXCLUDE<br>/PRINT CPS DESCRIPTIVES MODELINFO FIT ...
Resources	Processor Time	00:00:00.06
	Elapsed Time	00:00:00.06

Model Information

Dependent Variable	KneeFlexionRT
Probability Distribution	Gamma
Link Function	Log
Subject Effect 1	Subject
Working Correlation Matrix Structure	Independent

Case Processing Summary

	N	Percent
Included	48	100.0%
Excluded	0	0.0%
Total	48	100.0%

Correlated Data Summary

Number of Levels	Subject Effect	Subject	13
Number of Subjects			13
Number of Measurements per Subject	Minimum		2
	Maximum		4
Correlation Matrix Dimension			4

Categorical Variable Information

			N	Percent
Factor	Terrain	Regular	26	54.2%
		Irregular	22	45.8%
		Total	48	100.0%
	Running	Free	24	50.0%
		Nordic	24	50.0%
		Total	48	100.0%

Continuous Variable Information

		N	Minimum	Maximum	Mean
Dependent Variable	KneeFlexionRT	48	20.6	37.7	29.379

Continuous Variable Information

		Std. Deviation
Dependent Variable	KneeFlexionRT	3.9128

Goodness of Fit[a]

	Value
Quasi Likelihood under Independence Model Criterion (QIC)[b]	8.570
Corrected Quasi Likelihood under Independence Model Criterion (QICC)[b]	8.688

Dependent Variable: KneeFlexionRT
Model: (Intercept), Terrain, Running, Terrain * Running

a. Information criteria are in smaller-is-better form.

b. Computed using the full log quasi-likelihood function.

Tests of Model Effects

Source	Type III Wald Chi-Square	df	Sig.
(Intercept)	13547.117	1	.000
Terrain	13.076	1	.000
Running	5.004	1	.025
Terrain * Running	.021	1	.885

Dependent Variable: KneeFlexionRT
Model: (Intercept), Terrain, Running, Terrain * Running

Parameter Estimates

Parameter	B	Std. Error	95% Wald Confidence Interval Lower	Upper	Hypothesis Test Wald Chi-Square
(Intercept)	3.454	.0316	3.392	3.516	11928.944
[Terrain=1.000]	-.109	.0288	-.165	-.052	14.239
[Terrain=2.000]	0[a]	.	.	.	.
[Running=1.00]	-.032	.0216	-.074	.011	2.131
[Running=2.00]	0[a]	.	.	.	.
[Terrain=1.000] * [Running=1.00]	-.004	.0279	-.059	.051	.021
[Terrain=1.000] * [Running=2.00]	0[a]	.	.	.	.
[Terrain=2.000] * [Running=1.00]	0[a]	.	.	.	.
[Terrain=2.000] * [Running=2.00]	0[a]	.	.	.	.
(Scale)	.015				

Parameter Estimates

Parameter	Hypothesis Test df	Sig.
(Intercept)	1	.000
[Terrain=1.000]	1	.000
[Terrain=2.000]	.	.
[Running=1.00]	1	.144
[Running=2.00]	.	.
[Terrain=1.000] * [Running=1.00]	1	.885
[Terrain=1.000] * [Running=2.00]	.	.
[Terrain=2.000] * [Running=1.00]	.	.
[Terrain=2.000] * [Running=2.00]	.	.
(Scale)		

Dependent Variable: KneeFlexionRT
Model: (Intercept), Terrain, Running, Terrain * Running

a. Set to zero because this parameter is redundant.

Estimated Marginal Means 1: Terrain

Estimates

Terrain	Mean	Std. Error	95% Wald Confidence Interval Lower	95% Wald Confidence Interval Upper
Regular	27.881	.8704	26.226	29.640
Irregular	31.140	1.0709	29.110	33.312

Pairwise Comparisons

(I) Terrain	(J) Terrain	Mean Difference (I-J)	Std. Error	df	Bonferroni Sig.	95% Wald Confidence ... Lower
Regular	Irregular	-3.259[a]	.9181	1	.000	-5.059
Irregular	Regular	3.259[a]	.9181	1	.000	1.460

Pairwise Comparisons

(I) Terrain	(J) Terrain	95% Wald Confidence ... Upper
Regular	Irregular	-1.460
Irregular	Regular	5.059

Pairwise comparisons of estimated marginal means based on the original scale of dependent variable KneeFlexionRT

a. The mean difference is significant at the .05 level.

Overall Test Results

Wald Chi-Square	df	Sig.
12.600	1	.000

The Wald chi-square tests the effect of Terrain. This test is based on the linearly independent pairwise comparisons among the estimated marginal means.

Estimated Marginal Means 2: Running

Estimates

Running	Mean	Std. Error	95% Wald Confidence Interval Lower	95% Wald Confidence Interval Upper
Free	28.975	.9610	27.151	30.921
Nordic	29.965	.7942	28.448	31.562

Pairwise Comparisons

(I) Running	(J) Running	Mean Difference (I-J)	Std. Error	df	Bonferroni Sig.	95% Wald Confidence ... Lower
Free	Nordic	-.990[a]	.4302	1	.021	-1.833
Nordic	Free	.990[a]	.4302	1	.021	.147

Pairwise Comparisons

(I) Running	(J) Running	95% Wald Confidence ... Upper
Free	Nordic	-.147
Nordic	Free	1.833

Pairwise comparisons of estimated marginal means based on the original scale of dependent variable KneeFlexionRT

a. The mean difference is significant at the .05 level.

Overall Test Results

Wald Chi-Square	df	Sig.
5.294	1	.021

The Wald chi-square tests the effect of Running. This test is based on the linearly independent pairwise comparisons among the estimated marginal means.

Estimated Marginal Means 3: Terrain* Running

Estimates

Terrain	Running	Mean	Std. Error	95% Wald Confidence Interval Lower	95% Wald Confidence Interval Upper
Regular	Free	27.389	.9940	25.509	29.408
	Nordic	28.382	.8120	26.834	30.019
Irregular	Free	30.653	1.2257	28.342	33.152
	Nordic	31.636	1.0005	29.734	33.659

Pairwise Comparisons

(I) Running*Terrain	(J) Running*Terrain	Mean Difference (I-J)	Std. Error	df
[Running=1.00]*[Terrain=1.000]	[Running=1.00]*[Terrain=2.000]	-3.263[a]	1.1155	1
	[Running=2.00]*[Terrain=1.000]	-.993	.5266	1
	[Running=2.00]*[Terrain=2.000]	-4.247[a]	.8774	1
[Running=1.00]*[Terrain=2.000]	[Running=1.00]*[Terrain=1.000]	3.263[a]	1.1155	1
	[Running=2.00]*[Terrain=1.000]	2.271	1.1337	1
	[Running=2.00]*[Terrain=2.000]	-.983	.6606	1
[Running=2.00]*[Terrain=1.000]	[Running=1.00]*[Terrain=1.000]	.993	.5266	1
	[Running=1.00]*[Terrain=2.000]	-2.271	1.1337	1
	[Running=2.00]*[Terrain=2.000]	-3.254[a]	.8776	1
[Running=2.00]*[Terrain=2.000]	[Running=1.00]*[Terrain=1.000]	4.247[a]	.8774	1
	[Running=1.00]*[Terrain=2.000]	.983	.6606	1
	[Running=2.00]*[Terrain=1.000]	3.254[a]	.8776	1

Pairwise Comparisons

(I) Running*Terrain	(J) Running*Terrain	Bonferroni Sig.	95% Wald Confidence ... Lower
[Running=1.00]*[Terrain=1.000]	[Running=1.00]*[Terrain=2.000]	.021	-6.206
	[Running=2.00]*[Terrain=1.000]	.356	-2.382
	[Running=2.00]*[Terrain=2.000]	.000	-6.561
[Running=1.00]*[Terrain=2.000]	[Running=1.00]*[Terrain=1.000]	.021	.320
	[Running=2.00]*[Terrain=1.000]	.271	-.720
	[Running=2.00]*[Terrain=2.000]	.820	-2.726
[Running=2.00]*[Terrain=1.000]	[Running=1.00]*[Terrain=1.000]	.356	-.396
	[Running=1.00]*[Terrain=2.000]	.271	-5.262
	[Running=2.00]*[Terrain=2.000]	.001	-5.569
[Running=2.00]*[Terrain=2.000]	[Running=1.00]*[Terrain=1.000]	.000	1.932
	[Running=1.00]*[Terrain=2.000]	.820	-.760
	[Running=2.00]*[Terrain=1.000]	.001	.938

Pairwise Comparisons

(I) Running*Terrain	(J) Running*Terrain	95% Wald Confidence ... Upper
[Running=1.00]*[Terrain=1.000]	[Running=1.00]*[Terrain=2.000]	-.320
	[Running=2.00]*[Terrain=1.000]	.396
	[Running=2.00]*[Terrain=2.000]	-1.932
[Running=1.00]*[Terrain=2.000]	[Running=1.00]*[Terrain=1.000]	6.206
	[Running=2.00]*[Terrain=1.000]	5.262
	[Running=2.00]*[Terrain=2.000]	.760
[Running=2.00]*[Terrain=1.000]	[Running=1.00]*[Terrain=1.000]	2.382
	[Running=1.00]*[Terrain=2.000]	.720
	[Running=2.00]*[Terrain=2.000]	-.938
[Running=2.00]*[Terrain=2.000]	[Running=1.00]*[Terrain=1.000]	6.561
	[Running=1.00]*[Terrain=2.000]	2.726
	[Running=2.00]*[Terrain=1.000]	5.569

Pairwise comparisons of estimated marginal means based on the original scale of dependent variable KneeFlexionRT

a. The mean difference is significant at the .05 level.

Overall Test Results

Wald Chi-Square	df	Sig.
26.256	3	.000

The Wald chi-square tests the effect of Running*Terrain. This test is based on the linearly independent pairwise comparisons among the estimated marginal means.

```
GENLIN AnkleDorsiflexionRTBY Terrain Running (ORDER=ASCENDING)
```

```
  /MODEL Terrain Running Terrain*Running INTERCEPT=YES
 DISTRIBUTION=GAMMA LINK=LOG
  /CRITERIA SCALE=MLE PCONVERGE=1E-006(ABSOLUTE) SINGULAR=1E-012 ANALYSISTY
PE=3(WALD) CILEVEL=95
    LIKELIHOOD=FULL
  /EMMEANS TABLES=Terrain SCALE=ORIGINAL COMPARE=Terrain CONTRAST=PAIRWISE
PADJUST=BONFERRONI
  /EMMEANS TABLES=Running SCALE=ORIGINAL COMPARE=Running CONTRAST=PAIRWISE
PADJUST=BONFERRONI
  /EMMEANS TABLES=Terrain*Running SCALE=ORIGINAL COMPARE=Running CONTRAST=P
AIRWISE
    PADJUST=BONFERRONI
 /EMMEANS TABLES=Terrain*Running SCALE=ORIGINAL COMPARE=Terrain CONTRAST=PA
IRWISE
    PADJUST=BONFERRONI
  /REPEATED SUBJECT=Subject SORT=YES CORRTYPE=INDEPENDENT ADJUSTCORR=YES CO
VB=ROBUST
  /MISSING CLASSMISSING=EXCLUDE
  /PRINT CPS DESCRIPTIVES MODELINFO FIT SUMMARY SOLUTION.
```

Generalized Linear Models

Notes

Output Created		31-OCT-2023 12:50:46
Comments		
Input	Data	C:\Users\andre\Documents\Andre\Pesquisa\Artigos para Publicar\Nordic Running\Data\Statistics\SPSS\Angular_Parameters_Range_Contact.sav
	Active Dataset	DataSet11
	Filter	<none>
	Weight	<none>
	Split File	<none>
	N of Rows in Working Data File	48
Missing Value Handling	Definition of Missing	User-defined missing values for factor, subject and within-subject variables are treated as missing.
	Cases Used	Statistics are based on cases with valid data for all variables in the model.
Weight Handling		not applicable

Notes

Syntax	GENLIN AnkleDorsiflexionRT BY Terrain Running (ORDER=ASCENDING)<br>/MODEL Terrain Running Terrain*Running INTERCEPT=YES<br>DISTRIBUTION=GAMMA LINK=LOG<br>/CRITERIA SCALE=MLE PCONVERGE=1E-006 (ABSOLUTE) SINGULAR=1E-012 ANALYSISTYPE=3 (WALD) CILEVEL=95<br>LIKELIHOOD=FULL<br>/EMMEANS TABLES=Terrain SCALE=ORIGINAL COMPARE=Terrain CONTRAST=PAIRWISE PADJUST=BONFERRONI<br>/EMMEANS TABLES=Running SCALE=ORIGINAL COMPARE=Running CONTRAST=PAIRWISE PADJUST=BONFERRONI<br>/EMMEANS TABLES=Terrain*Running SCALE=ORIGINAL COMPARE=Running CONTRAST=PAIRWISE<br><br>PADJUST=BONFERRONI<br>/EMMEANS TABLES=Terrain*Running SCALE=ORIGINAL COMPARE=Terrain CONTRAST=PAIRWISE<br><br>PADJUST=BONFERRONI<br>/REPEATED SUBJECT=Subject SORT=YES CORRTYPE=INDEPENDENT ADJUSTCORR=YES COVB=ROBUST<br>/MISSING CLASSMISSING=EXCLUDE<br>/PRINT CPS DESCRIPTIVES MODELINFO FIT SUMMARY SOLUTION.

Notes

Resources	Processor Time	00:00:00.05
	Elapsed Time	00:00:00.05

Model Information

Dependent Variable		AnkleDorsiflexionRT
Probability Distribution		Gamma
Link Function		Log
Subject Effect	1	Subject
Working Correlation Matrix Structure		Independent

Case Processing Summary

	N	Percent
Included	48	100.0%
Excluded	0	0.0%
Total	48	100.0%

Correlated Data Summary

Number of Levels	Subject Effect	Subject	13
Number of Subjects			13
Number of Measurements per Subject	Minimum		2
	Maximum		4
Correlation Matrix Dimension			4

Categorical Variable Information

			N	Percent
Factor	Terrain	Regular	26	54.2%
		Irregular	22	45.8%
		Total	48	100.0%
	Running	Free	24	50.0%
		Nordic	24	50.0%
		Total	48	100.0%

Continuous Variable Information

		N	Minimum	Maximum	Mean
Dependent Variable	AnkleDorsiflexionRT	48	23.9	48.1	39.749

Continuous Variable Information

		Std. Deviation
Dependent Variable	AnkleDorsiflexionRT	4.9350

Goodness of Fit[a]

	Value
Quasi Likelihood under Independence Model Criterion (QIC)[b]	7.692
Corrected Quasi Likelihood under Independence Model Criterion (QICC)[b]	8.745

Dependent Variable: AnkleDorsiflexionRT
Model: (Intercept), Terrain, Running, Terrain * Running

a. Information criteria are in smaller-is-better form.

b. Computed using the full log quasi-likelihood function.

Tests of Model Effects

Source	Type III		
	Wald Chi-Square	df	Sig.
(Intercept)	16235.371	1	.000
Terrain	5.114	1	.024
Running	.061	1	.806
Terrain * Running	16.959	1	.000

Dependent Variable: AnkleDorsiflexionRT
Model: (Intercept), Terrain, Running, Terrain * Running

Parameter Estimates

Parameter	B	Std. Error	95% Wald Confidence Interval Lower	Upper	Hypothesis Test Wald Chi-Square
(Intercept)	3.686	.0289	3.630	3.743	16263.768
[Terrain=1.000]	.000	.0332	-.065	.065	.000
[Terrain=2.000]	0[a]	.	.	.	.
[Running=1.00]	.051	.0234	.005	.097	4.738
[Running=2.00]	0[a]	.	.	.	.
[Terrain=1.000] * [Running=1.00]	-.111	.0269	-.163	-.058	16.959
[Terrain=1.000] * [Running=2.00]	0[a]	.	.	.	.
[Terrain=2.000] * [Running=1.00]	0[a]	.	.	.	.
[Terrain=2.000] * [Running=2.00]	0[a]	.	.	.	.
(Scale)	.015				

Parameter Estimates

Parameter	Hypothesis Test df	Sig.
(Intercept)	1	.000
[Terrain=1.000]	1	.997
[Terrain=2.000]	.	.
[Running=1.00]	1	.030
[Running=2.00]	.	.
[Terrain=1.000] * [Running=1.00]	1	.000
[Terrain=1.000] * [Running=2.00]	.	.
[Terrain=2.000] * [Running=1.00]	.	.
[Terrain=2.000] * [Running=2.00]	.	.
(Scale)		

Dependent Variable: AnkleDorsiflexionRT
Model: (Intercept), Terrain, Running, Terrain * Running

a. Set to zero because this parameter is redundant.

Estimated Marginal Means 1: Terrain

Estimates

Terrain	Mean	Std. Error	95% Wald Confidence Interval Lower	Upper
Regular	38.723	1.3233	36.214	41.405
Irregular	40.929	1.1609	38.716	43.269

Pairwise Comparisons

(I) Terrain	(J) Terrain	Mean Difference (I-J)	Std. Error	df	Bonferroni Sig.	95% Wald Confidence ... Lower
Regular	Irregular	-2.207[a]	.9617	1	.022	-4.092
Irregular	Regular	2.207[a]	.9617	1	.022	.322

Pairwise Comparisons

(I) Terrain	(J) Terrain	95% Wald Confidence ... Upper
Regular	Irregular	-.322
Irregular	Regular	4.092

Pairwise comparisons of estimated marginal means based on the original scale of dependent variable AnkleDorsiflexionRT

a. The mean difference is significant at the .05 level.

Overall Test Results

Wald Chi-Square	df	Sig.
5.265	1	.022

The Wald chi-square tests the effect of Terrain. This test is based on the linearly independent pairwise comparisons among the estimated marginal means.

Estimated Marginal Means 2: Running

Estimates

Running	Mean	Std. Error	95% Wald Confidence Interval Lower	Upper
Free	39.725	1.3039	37.250	42.365
Nordic	39.896	1.0895	37.817	42.089

Pairwise Comparisons

(I) Running	(J) Running	Mean Difference (I-J)	Std. Error	df	Bonferroni Sig.	95% Wald Confidence ... Lower
Free	Nordic	-.170	.6903	1	.805	-1.523
Nordic	Free	.170	.6903	1	.805	-1.183

Pairwise Comparisons

(I) Running	(J) Running	95% Wald Confidence ... Upper
Free	Nordic	1.183
Nordic	Free	1.523

Pairwise comparisons of estimated marginal means based on the original scale of dependent variable AnkleDorsiflexionRT

Overall Test Results

Wald Chi-Square	df	Sig.
.061	1	.805

The Wald chi-square tests the effect of Running. This test is based on the linearly independent pairwise comparisons among the estimated marginal means.

Estimated Marginal Means 3: Terrain* Running

Estimates

Terrain	Running	Mean	Std. Error	95% Wald Confidence Interval	
				Lower	Upper
Regular	Free	37.586	1.3738	34.987	40.377
	Nordic	39.894	1.3861	37.267	42.705
Irregular	Free	41.987	1.3595	39.405	44.738
	Nordic	39.898	1.1533	37.701	42.224

Pairwise Comparisons

Terrain	(I) Running	(J) Running	Mean Difference (I-J)	Std. Error	df	Bonferroni Sig.
Regular	Free	Nordic	-2.308[a]	.7861	1	.003
	Nordic	Free	2.308[a]	.7861	1	.003
Irregular	Free	Nordic	2.089[a]	.9711	1	.031
	Nordic	Free	-2.089[a]	.9711	1	.031

Pairwise Comparisons

Terrain	(I) Running	(J) Running	95% Wald Confidence Interval for Difference	
			Lower	Upper
Regular	Free	Nordic	-3.848	-.767
	Nordic	Free	.767	3.848
Irregular	Free	Nordic	.185	3.992
	Nordic	Free	-3.992	-.185

Pairwise comparisons of estimated marginal means based on the original scale of dependent variable AnkleDorsiflexionRT

a. The mean difference is significant at the .05 level.

Overall Test Results

Terrain	Wald Chi-Square	df	Sig.
Regular	8.618	1	.003
Irregular	4.627	1	.031

Each Wald chi-square tests the simple effects of Running within each level combination of the other factors shown. These tests are based on the linearly independent pairwise comparisons among the estimated marginal means.

Estimated Marginal Means 4: Terrain* Running

Estimates

Terrain	Running	Mean	Std. Error	95% Wald Confidence Interval	
				Lower	Upper
Regular	Free	37.586	1.3738	34.987	40.377
	Nordic	39.894	1.3861	37.267	42.705
Irregular	Free	41.987	1.3595	39.405	44.738
	Nordic	39.898	1.1533	37.701	42.224

Pairwise Comparisons

Running	(I) Terrain	(J) Terrain	Mean Difference (I-J)	Std. Error	df	Bonferroni Sig.
Free	Regular	Irregular	-4.401[a]	.8354	1	.000
	Irregular	Regular	4.401[a]	.8354	1	.000
Nordic	Regular	Irregular	-.005	1.3247	1	.997
	Irregular	Regular	.005	1.3247	1	.997

Pairwise Comparisons

Running	(I) Terrain	(J) Terrain	95% Wald Confidence Interval for Difference	
			Lower	Upper
Free	Regular	Irregular	-6.039	-2.764
	Irregular	Regular	2.764	6.039
Nordic	Regular	Irregular	-2.601	2.592
	Irregular	Regular	-2.592	2.601

Pairwise comparisons of estimated marginal means based on the original scale of dependent variable AnkleDorsiflexionRT

a. The mean difference is significant at the .05 level.

Overall Test Results

Running	Wald Chi-Square	df	Sig.
Free	27.753	1	.000
Nordic	.000	1	.997

Each Wald chi-square tests the simple effects of Terrain within each level combination of the other factors shown. These tests are based on the linearly independent pairwise comparisons among the estimated marginal means.

```
DATASET ACTIVATE DataSet10.
GENLIN HipFlexionRT BY Terrain Running (ORDER=ASCENDING)
  /MODEL Terrain Running Terrain*Running INTERCEPT=YES
 DISTRIBUTION=GAMMA LINK=LOG
  /CRITERIA SCALE=MLE PCONVERGE=1E-006(ABSOLUTE) SINGULAR=1E-012 ANALYSISTY
PE=3(WALD) CILEVEL=95
    LIKELIHOOD=FULL
  /EMMEANS TABLES=Terrain SCALE=ORIGINAL COMPARE=Terrain CONTRAST=PAIRWISE
PADJUST=BONFERRONI
```

```
  /EMMEANS TABLES=Running SCALE=ORIGINAL COMPARE=Running CONTRAST=PAIRWISE
PADJUST=BONFERRONI
  /EMMEANS TABLES=Terrain*Running SCALE=ORIGINAL COMPARE=Running*Terrain CO
NTRAST=PAIRWISE
    PADJUST=BONFERRONI
  /REPEATED SUBJECT=Subject SORT=YES CORRTYPE=INDEPENDENT ADJUSTCORR=YES CO
VB=ROBUST
  /MISSING CLASSMISSING=EXCLUDE
  /PRINT CPS DESCRIPTIVES MODELINFO FIT SUMMARY SOLUTION.
```

JOINT'S ROM - AERIAL PHASE

Generalized Linear Models

Notes

Output Created		31-OCT-2023 12:51:48
Comments		
Input	Data	C:\Users\andre\Documents\Andre\Pesquisa\Artigos para Publicar\Nordic Running\Data\Statistics\SPSS\Angular_Parameters_Range_Swing.sav
	Active Dataset	DataSet10
	Filter	<none>
	Weight	<none>
	Split File	<none>
	N of Rows in Working Data File	48
Missing Value Handling	Definition of Missing	User-defined missing values for factor, subject and within-subject variables are treated as missing.
	Cases Used	Statistics are based on cases with valid data for all variables in the model.
Weight Handling		not applicable

Notes

Syntax		GENLIN HipFlexionRT BY Terrain Running (ORDER=ASCENDING)<br>/MODEL Terrain Running Terrain*Running INTERCEPT=YES<br>DISTRIBUTION=GAMMA LINK=LOG<br>/CRITERIA SCALE=MLE PCONVERGE=1E-006 (ABSOLUTE) SINGULAR=1E-012 ANALYSISTYPE=3 (WALD) CILEVEL=95<br>LIKELIHOOD=FULL<br>/EMMEANS TABLES=Terrain SCALE=ORIGINAL COMPARE=Terrain CONTRAST=PAIRWISE PADJUST=BONFERRONI<br>/EMMEANS TABLES=Running SCALE=ORIGINAL COMPARE=Running CONTRAST=PAIRWISE PADJUST=BONFERRONI<br>/EMMEANS TABLES=Terrain*Running SCALE=ORIGINAL COMPARE=Running*Terrain CONTRAST=PAIRWISE<br><br>PADJUST=BONFERRONI<br>/REPEATED SUBJECT=Subject SORT=YES CORRTYPE=INDEPENDENT ADJUSTCORR=YES COVB=ROBUST<br>/MISSING CLASSMISSING=EXCLUDE<br>/PRINT CPS DESCRIPTIVES MODELINFO FIT SUMMARY SOLUTION.
Resources	Processor Time	00:00:00.08
	Elapsed Time	00:00:00.07

```
[DataSet10] C:\Users\andre\Documents\Andre\Pesquisa\Artigos para Publicar\Nordic Running\Data\Statistics\SPSS\Angular_Parameters_Range_Swing.sav
```

Model Information

Dependent Variable		HipFlexionRT
Probability Distribution		Gamma
Link Function		Log
Subject Effect	1	Subject
Working Correlation Matrix Structure		Independent

Case Processing Summary

	N	Percent
Included	48	100.0%
Excluded	0	0.0%
Total	48	100.0%

Correlated Data Summary

Number of Levels	Subject Effect	Subject	13
Number of Subjects			13
Number of Measurements per Subject	Minimum		2
	Maximum		4
Correlation Matrix Dimension			4

Categorical Variable Information

			N	Percent
Factor	Terrain	Regular	26	54.2%
		Irregular	22	45.8%
		Total	48	100.0%
	Running	Free	24	50.0%
		Nordic	24	50.0%
		Total	48	100.0%

Continuous Variable Information

		N	Minimum	Maximum	Mean	Std. Deviation
Dependent Variable	HipFlexionRT	48	48.3	85.1	67.902	10.9066

Goodness of Fit[a]

	Value
Quasi Likelihood under Independence Model Criterion (QIC)[b]	8.426
Corrected Quasi Likelihood under Independence Model Criterion (QICC)[b]	8.890

Dependent Variable: HipFlexionRT
Model: (Intercept), Terrain, Running, Terrain * Running

a. Information criteria are in smaller-is-better form.

b. Computed using the full log quasi-likelihood function.

Tests of Model Effects

Source	Type III		
	Wald Chi-Square	df	Sig.
(Intercept)	18546.813	1	.000
Terrain	35.982	1	.000
Running	.546	1	.460
Terrain * Running	.596	1	.440

Dependent Variable: HipFlexionRT
Model: (Intercept), Terrain, Running, Terrain * Running

Parameter Estimates

Parameter	B	Std. Error	95% Wald Confidence Interval		Hypothesis Test
			Lower	Upper	Wald Chi-Square
(Intercept)	4.310	.0270	4.257	4.363	25522.516
[Terrain=1.00]	-.195	.0332	-.260	-.130	34.493
[Terrain=2.00]	0[a]	.	.	.	.
[Running=1.00]	.005	.0161	-.027	.036	.088
[Running=2.00]	0[a]	.	.	.	.
[Terrain=1.00] * [Running=1.00]	.026	.0338	-.040	.092	.596
[Terrain=1.00] * [Running=2.00]	0[a]	.	.	.	.
[Terrain=2.00] * [Running=1.00]	0[a]	.	.	.	.
[Terrain=2.00] * [Running=2.00]	0[a]	.	.	.	.
(Scale)	.020				

Parameter Estimates

Parameter	Hypothesis Test	
	df	Sig.
(Intercept)	1	.000
[Terrain=1.00]	1	.000
[Terrain=2.00]	.	.
[Running=1.00]	1	.767
[Running=2.00]	.	.
[Terrain=1.00] * [Running=1.00]	1	.440
[Terrain=1.00] * [Running=2.00]	.	.
[Terrain=2.00] * [Running=1.00]	.	.
[Terrain=2.00] * [Running=2.00]	.	.
(Scale)		

Dependent Variable: HipFlexionRT
Model: (Intercept), Terrain, Running, Terrain * Running

a. Set to zero because this parameter is redundant.

Estimated Marginal Means 1: Terrain

Estimates

Terrain	Mean	Std. Error	95% Wald Confidence Interval Lower	Upper
Regular	62.212	2.4671	57.560	67.241
Irregular	74.618	2.1217	70.573	78.894

Pairwise Comparisons

(I) Terrain	(J) Terrain	Mean Difference (I-J)	Std. Error	df	Bonferroni Sig.	95% Wald Confidence ... Lower
Regular	Irregular	-12.405[a]	1.9493	1	.000	-16.226
Irregular	Regular	12.405[a]	1.9493	1	.000	8.585

Pairwise Comparisons

(I) Terrain	(J) Terrain	95% Wald Confidence ... Upper
Regular	Irregular	-8.585
Irregular	Regular	16.226

Pairwise comparisons of estimated marginal means based on the original scale of dependent variable HipFlexionRT

a. The mean difference is significant at the .05 level.

Overall Test Results

Wald Chi-Square	df	Sig.
40.500	1	.000

The Wald chi-square tests the effect of Terrain. This test is based on the linearly independent pairwise comparisons among the estimated marginal means.

Estimated Marginal Means 2: Running

Estimates

Running	Mean	Std. Error	95% Wald Confidence Interval Lower	Upper
Free	68.743	2.5381	63.944	73.902
Nordic	67.529	1.9672	63.782	71.497

Pairwise Comparisons

(I) Running	(J) Running	Mean Difference (I-J)	Std. Error	df	Bonferroni Sig.	95% Wald Confidence ... Lower
Free	Nordic	1.213	1.6547	1	.463	-2.030
Nordic	Free	-1.213	1.6547	1	.463	-4.456

Pairwise Comparisons

(I) Running	(J) Running	95% Wald Confidence ... Upper
Free	Nordic	4.456
Nordic	Free	2.030

Pairwise comparisons of estimated marginal means based on the original scale of dependent variable HipFlexionRT

Overall Test Results

Wald Chi-Square	df	Sig.
.538	1	.463

The Wald chi-square tests the effect of Running. This test is based on the linearly independent pairwise comparisons among the estimated marginal means.

Estimated Marginal Means 3: Terrain* Running

Estimates

Terrain	Running	Mean	Std. Error	95% Wald Confidence Interval Lower	95% Wald Confidence Interval Upper
Regular	Free	63.179	3.0697	57.440	69.492
	Nordic	61.260	2.3882	56.754	66.125
Irregular	Free	74.796	2.3879	70.259	79.626
	Nordic	74.440	2.0083	70.606	78.482

Pairwise Comparisons

(I) Running*Terrain	(J) Running*Terrain	Mean Difference (I-J)	Std. Error	df
[Running=1.00]*[Terrain=1.00]	[Running=1.00]*[Terrain=2.00]	-11.617[a]	2.3086	1
	[Running=2.00]*[Terrain=1.00]	1.919	2.4083	1
	[Running=2.00]*[Terrain=2.00]	-11.261[a]	2.3664	1
[Running=1.00]*[Terrain=2.00]	[Running=1.00]*[Terrain=1.00]	11.617[a]	2.3086	1
	[Running=2.00]*[Terrain=1.00]	13.536[a]	2.6479	1
	[Running=2.00]*[Terrain=2.00]	.356	1.2066	1
[Running=2.00]*[Terrain=1.00]	[Running=1.00]*[Terrain=1.00]	-1.919	2.4083	1
	[Running=1.00]*[Terrain=2.00]	-13.536[a]	2.6479	1
	[Running=2.00]*[Terrain=2.00]	-13.180[a]	2.1230	1
[Running=2.00]*[Terrain=2.00]	[Running=1.00]*[Terrain=1.00]	11.261[a]	2.3664	1
	[Running=1.00]*[Terrain=2.00]	-.356	1.2066	1
	[Running=2.00]*[Terrain=1.00]	13.180[a]	2.1230	1

Pairwise Comparisons

(I) Running*Terrain	(J) Running*Terrain	Bonferroni Sig.	95% Wald Confidence ... Lower
[Running=1.00]*[Terrain=1.00]	[Running=1.00]*[Terrain=2.00]	.000	-17.707
	[Running=2.00]*[Terrain=1.00]	1.000	-4.435
	[Running=2.00]*[Terrain=2.00]	.000	-17.504
[Running=1.00]*[Terrain=2.00]	[Running=1.00]*[Terrain=1.00]	.000	5.526
	[Running=2.00]*[Terrain=1.00]	.000	6.550
	[Running=2.00]*[Terrain=2.00]	1.000	-2.827
[Running=2.00]*[Terrain=1.00]	[Running=1.00]*[Terrain=1.00]	1.000	-8.272
	[Running=1.00]*[Terrain=2.00]	.000	-20.522
	[Running=2.00]*[Terrain=2.00]	.000	-18.780
[Running=2.00]*[Terrain=2.00]	[Running=1.00]*[Terrain=1.00]	.000	5.018
	[Running=1.00]*[Terrain=2.00]	1.000	-3.540
	[Running=2.00]*[Terrain=1.00]	.000	7.579

Pairwise Comparisons

(I) Running*Terrain	(J) Running*Terrain	95% Wald Confidence ... Upper
[Running=1.00]*[Terrain=1.00]	[Running=1.00]*[Terrain=2.00]	-5.526
	[Running=2.00]*[Terrain=1.00]	8.272
	[Running=2.00]*[Terrain=2.00]	-5.018
[Running=1.00]*[Terrain=2.00]	[Running=1.00]*[Terrain=1.00]	17.707
	[Running=2.00]*[Terrain=1.00]	20.522
	[Running=2.00]*[Terrain=2.00]	3.540
[Running=2.00]*[Terrain=1.00]	[Running=1.00]*[Terrain=1.00]	4.435
	[Running=1.00]*[Terrain=2.00]	-6.550
	[Running=2.00]*[Terrain=2.00]	-7.579
[Running=2.00]*[Terrain=2.00]	[Running=1.00]*[Terrain=1.00]	17.504
	[Running=1.00]*[Terrain=2.00]	2.827
	[Running=2.00]*[Terrain=1.00]	18.780

Pairwise comparisons of estimated marginal means based on the original scale of dependent variable HipFlexionRT

a. The mean difference is significant at the .05 level.

Overall Test Results

Wald Chi-Square	df	Sig.
43.931	3	.000

The Wald chi-square tests the effect of Running*Terrain. This test is based on the linearly independent pairwise comparisons among the estimated marginal means.

```
GENLIN KneeFlexionRT BY Terrain Running (ORDER=ASCENDING)
```

```
  /MODEL Terrain Running Terrain*Running INTERCEPT=YES
  DISTRIBUTION=GAMMA LINK=LOG
  /CRITERIA SCALE=MLE PCONVERGE=1E-006(ABSOLUTE) SINGULAR=1E-012 ANALYSISTY
PE=3(WALD) CILEVEL=95
    LIKELIHOOD=FULL
  /EMMEANS TABLES=Terrain SCALE=ORIGINAL COMPARE=Terrain CONTRAST=PAIRWISE
PADJUST=BONFERRONI
  /EMMEANS TABLES=Running SCALE=ORIGINAL COMPARE=Running CONTRAST=PAIRWISE
PADJUST=BONFERRONI
  /EMMEANS TABLES=Terrain*Running SCALE=ORIGINAL COMPARE=Running*Terrain CO
NTRAST=PAIRWISE
    PADJUST=BONFERRONI
  /REPEATED SUBJECT=Subject SORT=YES CORRTYPE=INDEPENDENT ADJUSTCORR=YES CO
VB=ROBUST
  /MISSING CLASSMISSING=EXCLUDE
  /PRINT CPS DESCRIPTIVES MODELINFO FIT SUMMARY SOLUTION.
```

Generalized Linear Models

Notes

Output Created		31-OCT-2023 12:51:48
Comments		
Input	Data	C:\Users\andre\Documents\Andre\Pesquisa\Artigos para Publicar\Nordic Running\Data\Statistics\SPSS\Angular_Parameters_Range_Swing.sav
	Active Dataset	DataSet10
	Filter	<none>
	Weight	<none>
	Split File	<none>
	N of Rows in Working Data File	48
Missing Value Handling	Definition of Missing	User-defined missing values for factor, subject and within-subject variables are treated as missing.
	Cases Used	Statistics are based on cases with valid data for all variables in the model.
Weight Handling		not applicable

Notes

Syntax		GENLIN KneeFlexionRT BY Terrain Running (ORDER=ASCENDING)<br>/MODEL Terrain Running Terrain*Running INTERCEPT=YES<br><br>DISTRIBUTION=GAMMA LINK=LOG<br>/CRITERIA SCALE=MLE PCONVERGE=1E-006 (ABSOLUTE) SINGULAR=1E-012 ANALYSISTYPE=3 (WALD) CILEVEL=95<br>LIKELIHOOD=FULL<br>/EMMEANS TABLES=Terrain SCALE=ORIGINAL COMPARE=Terrain CONTRAST=PAIRWISE PADJUST=BONFERRONI<br>/EMMEANS TABLES=Running SCALE=ORIGINAL COMPARE=Running CONTRAST=PAIRWISE PADJUST=BONFERRONI<br>/EMMEANS TABLES=Terrain*Running SCALE=ORIGINAL COMPARE=Running*Terrain CONTRAST=PAIRWISE<br><br>PADJUST=BONFERRONI<br>/REPEATED SUBJECT=Subject SORT=YES CORRTYPE=INDEPENDENT ADJUSTCORR=YES COVB=ROBUST<br>/MISSING CLASSMISSING=EXCLUDE<br>/PRINT CPS DESCRIPTIVES MODELINFO FIT ...
Resources	Processor Time	00:00:00.05
	Elapsed Time	00:00:00.06

Model Information

Dependent Variable	KneeFlexionRT
Probability Distribution	Gamma
Link Function	Log
Subject Effect 1	Subject
Working Correlation Matrix Structure	Independent

Case Processing Summary

	N	Percent
Included	48	100.0%
Excluded	0	0.0%
Total	48	100.0%

Correlated Data Summary

Number of Levels	Subject Effect	Subject	13
Number of Subjects			13
Number of Measurements per Subject	Minimum		2
	Maximum		4
Correlation Matrix Dimension			4

Categorical Variable Information

			N	Percent
Factor	Terrain	Regular	26	54.2%
		Irregular	22	45.8%
		Total	48	100.0%
	Running	Free	24	50.0%
		Nordic	24	50.0%
		Total	48	100.0%

Continuous Variable Information

		N	Minimum	Maximum	Mean
Dependent Variable	KneeFlexionRT	48	62.3	118.6	87.140

Continuous Variable Information

		Std. Deviation
Dependent Variable	KneeFlexionRT	13.1869

Goodness of Fit[a]

	Value
Quasi Likelihood under Independence Model Criterion (QIC)[b]	8.331
Corrected Quasi Likelihood under Independence Model Criterion (QICC)[b]	8.744

Dependent Variable: KneeFlexionRT
Model: (Intercept), Terrain, Running, Terrain * Running

a. Information criteria are in smaller-is-better form.

b. Computed using the full log quasi-likelihood function.

Tests of Model Effects

Source	Type III Wald Chi-Square	df	Sig.
(Intercept)	26304.698	1	.000
Terrain	26.875	1	.000
Running	6.079	1	.014
Terrain * Running	.381	1	.537

Dependent Variable: KneeFlexionRT
Model: (Intercept), Terrain, Running, Terrain * Running

Parameter Estimates

Parameter	B	Std. Error	95% Wald Confidence Interval Lower	Upper	Hypothesis Test Wald Chi-Square
(Intercept)	4.530	.0330	4.466	4.595	18833.029
[Terrain=1.00]	-.176	.0313	-.237	-.114	31.472
[Terrain=2.00]	0[a]	.	.	.	.
[Running=1.00]	.047	.0205	.007	.087	5.209
[Running=2.00]	0[a]	.	.	.	.
[Terrain=1.00] * [Running=1.00]	.019	.0302	-.041	.078	.381
[Terrain=1.00] * [Running=2.00]	0[a]	.	.	.	.
[Terrain=2.00] * [Running=1.00]	0[a]	.	.	.	.
[Terrain=2.00] * [Running=2.00]	0[a]	.	.	.	.
(Scale)	.016				

Parameter Estimates

Parameter	Hypothesis Test df	Sig.
(Intercept)	1	.000
[Terrain=1.00]	1	.000
[Terrain=2.00]	.	.
[Running=1.00]	1	.022
[Running=2.00]	.	.
[Terrain=1.00] * [Running=1.00]	1	.537
[Terrain=1.00] * [Running=2.00]	.	.
[Terrain=2.00] * [Running=1.00]	.	.
[Terrain=2.00] * [Running=2.00]	.	.
(Scale)		

Dependent Variable: KneeFlexionRT
Model: (Intercept), Terrain, Running, Terrain * Running

a. Set to zero because this parameter is redundant.

Estimated Marginal Means 1: Terrain

Estimates

Terrain	Mean	Std. Error	95% Wald Confidence Interval Lower	Upper
Regular	80.429	2.7292	75.254	85.960
Irregular	94.995	2.8237	89.618	100.693

Pairwise Comparisons

(I) Terrain	(J) Terrain	Mean Difference (I-J)	Std. Error	df	Bonferroni Sig.	95% Wald Confidence ... Lower	Upper
Regular	Irregular	-14.566[a]	2.7839	1	.000	-20.022	-9.110
Irregular	Regular	14.566[a]	2.7839	1	.000	9.110	20.022

Pairwise comparisons of estimated marginal means based on the original scale of dependent variable KneeFlexionRT

a. The mean difference is significant at the .05 level.

Overall Test Results

Wald Chi-Square	df	Sig.
27.377	1	.000

The Wald chi-square tests the effect of Terrain. This test is based on the linearly independent pairwise comparisons among the estimated marginal means.

Estimated Marginal Means 2: Running

Estimates

Running	Mean	Std. Error	95% Wald Confidence Interval Lower	Upper
Free	89.899	2.9999	84.207	95.975
Nordic	84.988	2.1925	80.798	89.396

Pairwise Comparisons

(I) Running	(J) Running	Mean Difference (I-J)	Std. Error	df	Bonferroni Sig.	95% Wald Confidence ... Lower
Free	Nordic	4.911[a]	2.0443	1	.016	.904
Nordic	Free	-4.911[a]	2.0443	1	.016	-8.917

Pairwise Comparisons

(I) Running	(J) Running	95% Wald Confidence ... Upper
Free	Nordic	8.917
Nordic	Free	-.904

Pairwise comparisons of estimated marginal means based on the original scale of dependent variable KneeFlexionRT

a. The mean difference is significant at the .05 level.

Overall Test Results

Wald Chi-Square	df	Sig.
5.770	1	.016

The Wald chi-square tests the effect of Running. This test is based on the linearly independent pairwise comparisons among the estimated marginal means.

Estimated Marginal Means 3: Terrain* Running

Estimates

Terrain	Running	Mean	Std. Error	95% Wald Confidence Interval Lower	95% Wald Confidence Interval Upper
Regular	Free	83.106	3.8153	75.955	90.931
	Nordic	77.837	2.1061	73.817	82.077
Irregular	Free	97.246	2.8978	91.729	103.095
	Nordic	92.795	3.0634	86.981	98.998

Pairwise Comparisons

(I) Running*Terrain	(J) Running*Terrain	Mean Difference (I-J)	Std. Error	df
[Running=1.00]*[Terrain=1.00]	[Running=1.00]*[Terrain=2.00]	-14.140[a]	3.3414	1
	[Running=2.00]*[Terrain=1.00]	5.269	2.7508	1
	[Running=2.00]*[Terrain=2.00]	-9.689	4.0818	1
[Running=1.00]*[Terrain=2.00]	[Running=1.00]*[Terrain=1.00]	14.140[a]	3.3414	1
	[Running=2.00]*[Terrain=1.00]	19.409[a]	2.6413	1
	[Running=2.00]*[Terrain=2.00]	4.450	1.9329	1
[Running=2.00]*[Terrain=1.00]	[Running=1.00]*[Terrain=1.00]	-5.269	2.7508	1
	[Running=1.00]*[Terrain=2.00]	-19.409[a]	2.6413	1
	[Running=2.00]*[Terrain=2.00]	-14.958[a]	2.7841	1
[Running=2.00]*[Terrain=2.00]	[Running=1.00]*[Terrain=1.00]	9.689	4.0818	1
	[Running=1.00]*[Terrain=2.00]	-4.450	1.9329	1
	[Running=2.00]*[Terrain=1.00]	14.958[a]	2.7841	1

Pairwise Comparisons

(I) Running*Terrain	(J) Running*Terrain	Bonferroni Sig.	95% Wald Confidence ...
			Lower
[Running=1.00]*[Terrain=1.00]	[Running=1.00]*[Terrain=2.00]	.000	-22.955
	[Running=2.00]*[Terrain=1.00]	.333	-1.989
	[Running=2.00]*[Terrain=2.00]	.106	-20.458
[Running=1.00]*[Terrain=2.00]	[Running=1.00]*[Terrain=1.00]	.000	5.324
	[Running=2.00]*[Terrain=1.00]	.000	12.440
	[Running=2.00]*[Terrain=2.00]	.128	-.649
[Running=2.00]*[Terrain=1.00]	[Running=1.00]*[Terrain=1.00]	.333	-12.526
	[Running=1.00]*[Terrain=2.00]	.000	-26.377
	[Running=2.00]*[Terrain=2.00]	.000	-22.303
[Running=2.00]*[Terrain=2.00]	[Running=1.00]*[Terrain=1.00]	.106	-1.080
	[Running=1.00]*[Terrain=2.00]	.128	-9.550
	[Running=2.00]*[Terrain=1.00]	.000	7.613

Pairwise Comparisons

(I) Running*Terrain	(J) Running*Terrain	95% Wald Confidence ... Upper
[Running=1.00]*[Terrain=1.00]	[Running=1.00]*[Terrain=2.00]	-5.324
	[Running=2.00]*[Terrain=1.00]	12.526
	[Running=2.00]*[Terrain=2.00]	1.080
[Running=1.00]*[Terrain=2.00]	[Running=1.00]*[Terrain=1.00]	22.955
	[Running=2.00]*[Terrain=1.00]	26.377
	[Running=2.00]*[Terrain=2.00]	9.550
[Running=2.00]*[Terrain=1.00]	[Running=1.00]*[Terrain=1.00]	1.989
	[Running=1.00]*[Terrain=2.00]	-12.440
	[Running=2.00]*[Terrain=2.00]	-7.613
[Running=2.00]*[Terrain=2.00]	[Running=1.00]*[Terrain=1.00]	20.458
	[Running=1.00]*[Terrain=2.00]	.649
	[Running=2.00]*[Terrain=1.00]	22.303

Pairwise comparisons of estimated marginal means based on the original scale of dependent variable KneeFlexionRT

a. The mean difference is significant at the .05 level.

Overall Test Results

Wald Chi-Square	df	Sig.
54.049	3	.000

The Wald chi-square tests the effect of Running*Terrain. This test is based on the linearly independent pairwise comparisons among the estimated marginal means.

```
GENLIN AnkleDorsiflexionRTBY Terrain Running (ORDER=ASCENDING)
```

```
  /MODEL Terrain Running Terrain*Running INTERCEPT=YES
 DISTRIBUTION=GAMMA LINK=LOG
  /CRITERIA SCALE=MLE PCONVERGE=1E-006(ABSOLUTE) SINGULAR=1E-012 ANALYSISTY
PE=3(WALD) CILEVEL=95
    LIKELIHOOD=FULL
  /EMMEANS TABLES=Terrain SCALE=ORIGINAL COMPARE=Terrain CONTRAST=PAIRWISE
PADJUST=BONFERRONI
  /EMMEANS TABLES=Running SCALE=ORIGINAL COMPARE=Running CONTRAST=PAIRWISE
PADJUST=BONFERRONI
  /EMMEANS TABLES=Terrain*Running SCALE=ORIGINAL COMPARE=Running CONTRAST=P
AIRWISE
    PADJUST=BONFERRONI
 /EMMEANS TABLES=Terrain*Running SCALE=ORIGINAL COMPARE=Terrain CONTRAST=PA
IRWISE
    PADJUST=BONFERRONI
  /REPEATED SUBJECT=Subject SORT=YES CORRTYPE=INDEPENDENT ADJUSTCORR=YES CO
VB=ROBUST
  /MISSING CLASSMISSING=EXCLUDE
  /PRINT CPS DESCRIPTIVES MODELINFO FIT SUMMARY SOLUTION.
```

Generalized Linear Models

Notes

Output Created		31-OCT-2023 12:51:48
Comments		
Input	Data	C:\Users\andre\Documents\Andre\Pesquisa\Artigos para Publicar\Nordic Running\Data\Statistics\SPSS\Angular_Parameters_Range_Swing.sav
	Active Dataset	DataSet10
	Filter	<none>
	Weight	<none>
	Split File	<none>
	N of Rows in Working Data File	48
Missing Value Handling	Definition of Missing	User-defined missing values for factor, subject and within-subject variables are treated as missing.
	Cases Used	Statistics are based on cases with valid data for all variables in the model.
Weight Handling		not applicable

Notes

Syntax	GENLIN AnkleDorsiflexionRT BY Terrain Running (ORDER=ASCENDING)<br>/MODEL Terrain Running Terrain*Running INTERCEPT=YES<br>DISTRIBUTION=GAMMA LINK=LOG<br>/CRITERIA SCALE=MLE PCONVERGE=1E-006 (ABSOLUTE) SINGULAR=1E-012 ANALYSISTYPE=3 (WALD) CILEVEL=95<br>LIKELIHOOD=FULL<br>/EMMEANS TABLES=Terrain SCALE=ORIGINAL COMPARE=Terrain CONTRAST=PAIRWISE PADJUST=BONFERRONI<br>/EMMEANS TABLES=Running SCALE=ORIGINAL COMPARE=Running CONTRAST=PAIRWISE PADJUST=BONFERRONI<br>/EMMEANS TABLES=Terrain*Running SCALE=ORIGINAL COMPARE=Running CONTRAST=PAIRWISE<br><br>PADJUST=BONFERRONI<br>/EMMEANS TABLES=Terrain*Running SCALE=ORIGINAL COMPARE=Terrain CONTRAST=PAIRWISE<br><br>PADJUST=BONFERRONI<br>/REPEATED SUBJECT=Subject SORT=YES CORRTYPE=INDEPENDENT ADJUSTCORR=YES COVB=ROBUST<br>/MISSING CLASSMISSING=EXCLUDE<br>/PRINT CPS DESCRIPTIVES MODELINFO FIT SUMMARY SOLUTION.

Notes

Resources	Processor Time	00:00:00.05
	Elapsed Time	00:00:00.05

Model Information

Dependent Variable		AnkleDorsiflexionRT
Probability Distribution		Gamma
Link Function		Log
Subject Effect	1	Subject
Working Correlation Matrix Structure		Independent

Case Processing Summary

	N	Percent
Included	48	100.0%
Excluded	0	0.0%
Total	48	100.0%

Correlated Data Summary

Number of Levels	Subject Effect	Subject	13
Number of Subjects			13
Number of Measurements per Subject	Minimum		2
	Maximum		4
Correlation Matrix Dimension			4

Categorical Variable Information

			N	Percent
Factor	Terrain	Regular	26	54.2%
		Irregular	22	45.8%
		Total	48	100.0%
	Running	Free	24	50.0%
		Nordic	24	50.0%
		Total	48	100.0%

Continuous Variable Information

		N	Minimum	Maximum	Mean
Dependent Variable	AnkleDorsiflexionRT	48	14.7	43.7	34.231

Continuous Variable Information

		Std. Deviation
Dependent Variable	AnkleDorsiflexionRT	5.7179

Goodness of Fit[a]

	Value
Quasi Likelihood under Independence Model Criterion (QIC)[b]	8.135
Corrected Quasi Likelihood under Independence Model Criterion (QICC)[b]	9.557

Dependent Variable: AnkleDorsiflexionRT
Model: (Intercept), Terrain, Running, Terrain * Running

a. Information criteria are in smaller-is-better form.

b. Computed using the full log quasi-likelihood function.

Tests of Model Effects

	Type III		
Source	Wald Chi-Square	df	Sig.
(Intercept)	6604.921	1	.000
Terrain	.053	1	.818
Running	4.195	1	.041
Terrain * Running	4.583	1	.032

Dependent Variable: AnkleDorsiflexionRT
Model: (Intercept), Terrain, Running, Terrain * Running

Parameter Estimates

Parameter	B	Std. Error	95% Wald Confidence Interval		Hypothesis Test
			Lower	Upper	Wald Chi-Square
(Intercept)	3.529	.0459	3.439	3.619	5919.618
[Terrain=1.00]	.045	.0261	-.006	.096	2.984
[Terrain=2.00]	0[a]	.	.	.	.
[Running=1.00]	.001	.0288	-.056	.057	.001
[Running=2.00]	0[a]	.	.	.	.
[Terrain=1.00] * [Running=1.00]	-.080	.0373	-.153	-.007	4.583
[Terrain=1.00] * [Running=2.00]	0[a]	.	.	.	.
[Terrain=2.00] * [Running=1.00]	0[a]	.	.	.	.
[Terrain=2.00] * [Running=2.00]	0[a]	.	.	.	.
(Scale)	.029				

Parameter Estimates

Parameter	Hypothesis Test	
	df	Sig.
(Intercept)	1	.000
[Terrain=1.00]	1	.084
[Terrain=2.00]	.	.
[Running=1.00]	1	.975
[Running=2.00]	.	.
[Terrain=1.00] * [Running=1.00]	1	.032
[Terrain=1.00] * [Running=2.00]	.	.
[Terrain=2.00] * [Running=1.00]	.	.
[Terrain=2.00] * [Running=2.00]	.	.
(Scale)		

Dependent Variable: AnkleDorsiflexionRT
Model: (Intercept), Terrain, Running, Terrain * Running

a. Set to zero because this parameter is redundant.

Estimated Marginal Means 1: Terrain

Estimates

Terrain	Mean	Std. Error	95% Wald Confidence Interval Lower	Upper
Regular	34.298	1.6156	31.273	37.616
Irregular	34.119	1.4550	31.383	37.093

Pairwise Comparisons

(I) Terrain	(J) Terrain	Mean Difference (I-J)	Std. Error	df	Bonferroni Sig.	95% Wald Confidence ... Lower
Regular	Irregular	.179	.7801	1	.818	-1.350
Irregular	Regular	-.179	.7801	1	.818	-1.708

Pairwise Comparisons

(I) Terrain	(J) Terrain	95% Wald Confidence ... Upper
Regular	Irregular	1.708
Irregular	Regular	1.350

Pairwise comparisons of estimated marginal means based on the original scale of dependent variable AnkleDorsiflexionRT

Overall Test Results

Wald Chi-Square	df	Sig.
.053	1	.818

The Wald chi-square tests the effect of Terrain. This test is based on the linearly independent pairwise comparisons among the estimated marginal means.

Estimated Marginal Means 2: Running

Estimates

Running	Mean	Std. Error	95% Wald Confidence Interval Lower	Upper
Free	33.548	1.6134	30.530	36.864
Nordic	34.882	1.4157	32.215	37.771

Pairwise Comparisons

(I) Running	(J) Running	Mean Difference (I-J)	Std. Error	df	Bonferroni Sig.	95% Wald Confidence ... Lower
Free	Nordic	-1.335[a]	.6307	1	.034	-2.571
Nordic	Free	1.335[a]	.6307	1	.034	.099

Pairwise Comparisons

(I) Running	(J) Running	95% Wald Confidence ... Upper
Free	Nordic	-.099
Nordic	Free	2.571

Pairwise comparisons of estimated marginal means based on the original scale of dependent variable AnkleDorsiflexionRT

a. The mean difference is significant at the .05 level.

Overall Test Results

Wald Chi-Square	df	Sig.
4.479	1	.034

The Wald chi-square tests the effect of Running. This test is based on the linearly independent pairwise comparisons among the estimated marginal means.

Estimated Marginal Means 3: Terrain* Running

Estimates

Terrain	Running	Mean	Std. Error	95% Wald Confidence Interval Lower	95% Wald Confidence Interval Upper
Regular	Free	32.971	1.8656	29.510	36.838
	Nordic	35.679	1.3963	33.045	38.523
Irregular	Free	34.134	1.5065	31.306	37.218
	Nordic	34.104	1.5644	31.171	37.312

Pairwise Comparisons

Terrain	(I) Running	(J) Running	Mean Difference (I-J)	Std. Error	df	Bonferroni Sig.
Regular	Free	Nordic	-2.708[a]	.7465	1	.000
	Nordic	Free	2.708[a]	.7465	1	.000
Irregular	Free	Nordic	.031	.9830	1	.975
	Nordic	Free	-.031	.9830	1	.975

Pairwise Comparisons

Terrain	(I) Running	(J) Running	95% Wald Confidence Interval for Difference	
			Lower	Upper
Regular	Free	Nordic	-4.171	-1.245
	Nordic	Free	1.245	4.171
Irregular	Free	Nordic	-1.896	1.957
	Nordic	Free	-1.957	1.896

Pairwise comparisons of estimated marginal means based on the original scale of dependent variable AnkleDorsiflexionRT

a. The mean difference is significant at the .05 level.

Overall Test Results

Terrain	Wald Chi-Square	df	Sig.
Regular	13.160	1	.000
Irregular	.001	1	.975

Each Wald chi-square tests the simple effects of Running within each level combination of the other factors shown. These tests are based on the linearly independent pairwise comparisons among the estimated marginal means.

Estimated Marginal Means 4: Terrain* Running

Estimates

Terrain	Running	Mean	Std. Error	95% Wald Confidence Interval	
				Lower	Upper
Regular	Free	32.971	1.8656	29.510	36.838
	Nordic	35.679	1.3963	33.045	38.523
Irregular	Free	34.134	1.5065	31.306	37.218
	Nordic	34.104	1.5644	31.171	37.312

Pairwise Comparisons

Running	(I) Terrain	(J) Terrain	Mean Difference (I-J)	Std. Error	df	Bonferroni Sig.
Free	Regular	Irregular	-1.163	1.0648	1	.275
	Irregular	Regular	1.163	1.0648	1	.275
Nordic	Regular	Irregular	1.575	.8969	1	.079
	Irregular	Regular	-1.575	.8969	1	.079

Pairwise Comparisons

Running	(I) Terrain	(J) Terrain	95% Wald Confidence Interval for Difference	
			Lower	Upper
Free	Regular	Irregular	-3.250	.924
	Irregular	Regular	-.924	3.250
Nordic	Regular	Irregular	-.183	3.333
	Irregular	Regular	-3.333	.183

Pairwise comparisons of estimated marginal means based on the original scale of dependent variable AnkleDorsiflexionRT

Overall Test Results

Running	Wald Chi-Square	df	Sig.
Free	1.194	1	.275
Nordic	3.084	1	.079

Each Wald chi-square tests the simple effects of Terrain within each level combination of the other factors shown. These tests are based on the linearly independent pairwise comparisons among the estimated marginal means.
